# Effectiveness of a Health Coaching Intervention for Patient-Family Dyads to Improve Outcomes Among Adults With Diabetes: A Randomized Clinical Trial

**DOI:** 10.1001/jamanetworkopen.2022.37960

**Published:** 2022-11-14

**Authors:** Ann-Marie Rosland, John D. Piette, Ranak Trivedi, Aaron Lee, Shelley Stoll, Ada O. Youk, D. Scott Obrosky, Denise Deverts, Eve A. Kerr, Michele Heisler

**Affiliations:** 1 Center for Health Equity Research and Promotion, VA Pittsburgh Healthcare System, Pittsburgh, Pennsylvania; 2 Department of Internal Medicine, University of Pittsburgh School of Medicine, Pittsburgh, Pennsylvania; 3 Department of Health Behavior and Health Education, School of Public Health, University of Michigan, Ann Arbor; 4 Center for Clinical Management Research, VA Ann Arbor Healthcare System, Ann Arbor, Michigan; 5 Department of Psychiatry and Behavioral Sciences, Stanford University, Stanford, California; 6 Center for Innovation to Implementation, VA Palo Alto Healthcare System, Menlo Park, California; 7 Department of Psychology, University of Mississippi, University; 8 Department of Internal Medicine and the Institute for Healthcare Policy and Innovation, University of Michigan, Ann Arbor; 9 Department of Biostatistics, University of Pittsburgh Graduate School of Public Health, Pittsburgh, Pennsylvania

## Abstract

**Importance:**

More than 75% of US adults with diabetes do not meet treatment goals. More effective support from family and friends (“supporters”) may improve diabetes management and outcomes.

**Objective:**

To determine if the Caring Others Increasing Engagement in Patient Aligned Care Teams (CO-IMPACT) intervention improves patient activation, diabetes management, and outcomes compared with standard care.

**Design, Setting, and Participants:**

This randomized clinical trial was conducted from November 2016 to August 2019 among participants recruited from 2 Veterans Health Administration primary care sites. All patient participants were adults aged 30 to 70 years with diabetes who had hemoglobin A_1c_ (HbA_1c_) levels greater than 8% of total hemoglobin (to convert to proportion of total hemoglobin, multiply by 0.01) or systolic blood pressure (SBP) higher than 150 mm Hg; each participating patient had an adult supporter. Of 1119 recruited, 239 patient-supporter dyads were enrolled between November 2016 and May 2018, randomized 1:1 to receive the CO-IMPACT intervention or standard care, and followed up for 12 to 15 months. Investigators and analysts were blinded to group assignment.

**Interventions:**

Patient-supporter dyads received a health coaching session focused on dyadic information sharing and positive support techniques, then 12 months of biweekly automated monitoring telephone calls to prompt dyadic actions to meet diabetes goals, coaching calls to help dyads prepare for primary care visits, and after-visit summaries. Standard-care dyads received general diabetes education materials only.

**Main Outcomes and Measures:**

Intent-to-treat analyses were conducted according to baseline dyad assignment. Primary prespecified outcomes were 12-month changes in Patient Activation Measure–13 (PAM-13) and UK Prospective Diabetes Study (UKPDS) 5-year diabetes-specific cardiac event risk scores. Secondary outcomes included 12-month changes in HbA_1c_ levels, SBP, diabetes self-management behaviors, diabetes distress, diabetes management self-efficacy, and satisfaction with health system support for the involvement of family supporters. Changes in outcome measures between baseline and 12 months were analyzed using linear regression models.

**Results:**

A total of 239 dyads enrolled; among patient participants, the mean (SD) age was 60 (8.9) years, and 231 (96.7%) were male. The mean (SD) baseline HbA_1c_ level was 8.5% (1.6%) and SBP was 140.2 mm Hg (18.4 mm Hg). A total of 168 patients (70.3%) lived with their enrolled supporter; 229 patients (95.8%) had complete 12-month outcome data. In intention-to-treat analyses vs standard care, CO-IMPACT patients had greater 12-month improvements in PAM-13 scores (intervention effect, 2.60 points; 95% CI, 0.02-5.18 points; *P* = .048) but nonsignificant differences in UKPDS 5-year cardiac risk (intervention effect, 1.01 points; 95% CI, −0.74 to 2.77 points; *P* = .26). Patients in the CO-IMPACT arm also had greater 12-month improvements in healthy eating (intervention effect, 0.71 d/wk; 95% CI, 0.20-1.22 d/wk; *P* = .007), diabetes self-efficacy (intervention effect, 0.40 points; 95% CI, 0.09-0.71 points; *P* = .01), and satisfaction with health system support for the family supporter participants’ involvement (intervention effect, 0.28 points; 95% CI, 0.07-0.49 points; *P* = .009); however, the 2 arms had similar improvements in HbA_1c_ levels and in other measures.

**Conclusions and Relevance:**

In this randomized clinical trial, the CO-IMPACT intervention successfully engaged patient-supporter dyads and led to improved patient activation and self-efficacy. Physiological outcomes improved similarly in both arms. More intensive direct coaching of supporters, or targeting patients with less preexisting support or fewer diabetes management resources, may have greater impact.

**Trial Registration:**

ClinicalTrials.gov Identifier: NCT02328326

## Key Points

**Question:**

Does the Caring Others Increasing Engagement in Patient Aligned Care Teams (CO-IMPACT) intervention, which provides health coaching and automated monitoring telephone calls to dyads of adults with diabetes and their family supporters, improve patient activation, diabetes management, and outcomes compared with standard care?

**Findings:**

In this randomized clinical trial with 239 patient-supporter dyads, CO-IMPACT improved patient activation, diabetes self-efficacy, and healthy eating. There were no between-group differences in diabetes-specific cardiac risk or glycemic control.

**Meaning:**

Engaging family supporters in a low-intensity coaching and monitoring program improved patients’ capacity to manage diabetes, but a higher-intensity program may be necessary to improve outcomes.

## Teaser

This randomized clinical trial investigates whether an intervention to provide health coaching, training, and tools to dyads consisting of 1 adult patient with diabetes and 1 adult supporter improves patient activation and diabetes management compared with standard diabetes care.

## Introduction

More than 75% of US adults with diabetes do not meet all glycemic, blood pressure, and lipid treatment targets.^1^ These patients often need more intensive monitoring and support than health systems can provide. One potentially powerful source of support is patients’ family and friends (family supporters). Among adults with diabetes and without significant functional limitations, 50% to 75% have a family supporter who is regularly involved in their diabetes management.^2,3^ Support for diabetes care from family and friends is associated with better self-management behaviors and risk factor control and is associated with decreased risk of hospitalization and death.^4,5,6,7,8^ Recent Diabetes Self-management Education and Support (DSMES) guidelines call for health care professionals to actively include family supporters in diabetes education and support programs.^9^

Interventions that provide general diabetes information to family supporters have not improved patient outcomes.^10,11^ Studies on specific aspects of dyadic diabetes management have identified more promising approaches to increasing effective family support,^12^ including training family supporters in techniques to facilitate behavior change (eg, goal-setting,^13^ action planning,^14,15^ and use of autonomy-supportive communication styles^15,16,17,18^), enhancing supporter access to information about the patient’s diabetes regimen and test results,^19^ prompting supporters to routinely discuss diabetes management with the patient, and training supporters in techniques to support patient engagement in health care visits.^20^ However, health care teams do not have structured programs or tools to deliver these promising approaches to patient-supporter pairs.

To address this gap, we designed the Caring Others Increasing Engagement in Patient Aligned Care Teams (CO-IMPACT) intervention as a structured approach to providing training and tools to adult patient-supporter dyads. In this article, we report the main patient health, behavioral, and satisfaction outcomes of a 12-month randomized clinical trial of the CO-IMPACT intervention vs standard, comprehensive diabetes care. Our primary hypotheses were that patients’ activation, defined as “having the knowledge, skills, and confidence to manage one’s health,”^21^ would increase and that diabetes-specific cardiac risk would decrease more among patients receiving the CO-IMPACT intervention than among those receiving standard care.

## Methods

### Study Design and Setting

This randomized clinical trial was conducted from November 2016 to August 2019. Dyads consisting of 1 adult with diabetes plus 1 family supporter were recruited from 1 hospital-based and 1 community-based US Veterans Health Administration primary care clinic. Both clinics offered comprehensive diabetes care, including assignment to a patient-centered medical home team^22^; access to nurse care management, a clinical pharmacist, and dietitian services; and diabetes education and weight management classes. A detailed study protocol and rationale were previously published.^23^ The full initial and final study protocol and statistical analysis plans were approved by the Veterans Affairs (VA) Ann Arbor Institutional Review Board and are included as Supplement 1. All patients provided written informed consent, and supporters provided oral informed consent. Analyses and reporting followed the Consolidated Standards of Reporting Trials (CONSORT) reporting guideline.^24^

### Participants and Randomization

Potentially eligible patients were identified via administrative databases. Patient inclusion criteria included an age of 30 to 70 years; type 2 diabetes, based on the encounter diagnoses or a chronic diabetes medication prescription other than metformin; poor glycemic control (most recent hemoglobin A_1c_ [HbA_1c_] level in the past 9 months >8% of total hemoglobin) (to convert to proportion of total hemoglobin, multiply by 0.01) or poor blood pressure (BP) control (most recent systolic BP [SBP] >150 mm Hg and mean SBP during the past 9 months >150 mm Hg); 2 or more visits to the VA primary care clinic in the previous 12 months; English speaker; and able to communicate via touch-tone telephone. Exclusion criteria included diabetes managed by a specialist or non-VA physician, living in a nursing home or assisted living facility, requiring help with 2 or more basic activities of daily living, life-limiting severe illness, being pregnant or planning pregnancy, serious mental illness, moderate to severe cognitive impairment, or active substance use disorder.

Supporters were nominated by the patient participant. Supporter eligibility criteria included age 21 years or older, talking with the patient about their health at least twice per month, helping the patient regularly with 1 or more aspects of their health care (eg, filling medications, conducting home testing), not receiving pay to care for the patient, and no severe mental illness, dementia, life-limiting severe illness, or need for help with basic activities of daily living.

Participants were enrolled between November 2016 and May 2018 and were observed for up to 15 months after enrollment (until August 2019). Dyads were randomized using sequential treatment assignment with minimization in a 1:1 ratio between arms, initially balanced across strata of supporter-patient cohabitation (yes or no), using a level of determinism of 5 on a scale of 1 to 10.^25^ The randomization protocol was amended to add a second stratification criterion (Patient Activation Measure–13 [PAM-13^26^] score of ≥40 vs <40 [score range, 0-100, with higher scores indicating greater activation]) after interim analyses revealed that the mean baseline PAM-13 scores differed significantly between arms. Investigators and analysts were blinded to group assignment.

### Interventions

Dyads assigned to the CO-IMPACT arm received 4 program components over 12 months: an initial coaching session, biweekly automated interactive voice response (IVR) telephone calls, primary care visit preparation calls, and primary care visit summaries. Details on the intervention-component delivery mode and topics are included in Table 1 and the eAppendix in Supplement 2. Each coach-delivered component was designed to be usable within typical physician, diabetes educator, or nurse care management patient encounters. Study coaches had social work or public health degrees and received training in diabetes care, health behavior change principles, and dyadic communication. Coaches delivered the intervention using manualized scripts. Specific IVR call topics and information on accessing intervention materials are included in the eFigure in Supplement 2. Dyads assigned to either CO-IMPACT or standard care (termed *enhanced usual care* in initial protocols) were offered home glucose and BP monitoring equipment and were provided general diabetes management information via a study handbook and website. Patients in both arms could receive standard clinic diabetes care, which followed national VA clinical practice guidelines.^27^ Supporters in both arms were not limited from participating in any patient clinic visits or programs. Fidelity to intervention delivery was assessed via investigator (A.R., S.S., M.H.) review of all coach call and mailing records and of fidelity rating forms for recordings of 10% of the initial sessions and visit preparation calls.

**Table 1.** Intervention Component Details of the CO-IMPACT and Standard Care Arms^a^

Timeline	Component	Participants	Deliverer, mode	Content and topics addressed
Enrollment^b^	Self-monitoring equipment and general diabetes information	P, S	RA, in person	Patients in both arms were offered glucometers, blood pressure monitors, and a handbook with general diabetes information
Within 1 mo of enrollment	Initial 1-1.5 h coaching session	P, S	Dyad coach, synchronous in person	Patient’s current diabetes complications risk status and diabetes management regimen, including medications Positive communication techniques, emphasizing regular weekly talks about diabetes; open and nonjudgmental communication; and autonomy-supportive communication Dyadic approaches to action planning and engaging in medical visits; “who’s who” on the primary care team
	CO-IMPACT handbook and website	P, S	NA	Information on each topic discussed during the initial session Information on each topic covered in the IVR calls
From the date of the initial coaching session to a date 12 mo after the patient baseline survey (approximately 11 mo)	IVR calls every 2 wk	P	Automated phone call	Automated voice-response inquiries asked patient to (1) indicate whether any of several events occurred during the past 2 wk (high or low blood glucose or blood pressure, problems with home monitors, medication access or adherence concerns, sick days, new foot problems, and readiness to quit smoking) and (2) rate how important they felt identified issues were to address Tips on how to address important issues, including a prompt to make an action plan Prompts to engage in self-monitoring Selected issues flagged as urgent resulted in an automated fax to the patient’s primary care team
	IVR summary with follow-up tips	S	Automated email	Summary of patient’s responses to IVR calls as well as identified health issues and their importance to the patient Advice on how supporters could help patients address identified issues, with links to relevant content in the study handbook and website Reminders to use positive, autonomy-supportive communication with the patient
	Visit reminder 2-5 d before each scheduled clinic visit	S	Dyad coach, email	Reminder from the coach that the patient has an upcoming clinic appointment and to use the visit preparation worksheet to discuss the appointment with the patient Invitation to supporter to participate in the CO-IMPACT clinic-visit preparation call with the coach
	Visit preparation calls 2-5 d before each scheduled clinic visit	P and/or S	Dyad coach, synchronous telephone discussion	Prompts from the coach to list issues the patient wanted to discuss and information they wanted to bring to the clinic visit (such as home glucose logs) Suggestion from the coach that the patient indicate how they wanted their supporter to help them prepare for the visit or participate in the visit with them Request for supporter to suggest any additional issues they felt the patient should include in the visit
	Visit summaries	P, S	Dyad coach, mailed or posted to website automatically after a completed visit	Customized summary describing diabetes-related details of the patient’s recent clinic visit

Abbreviations: IVR, interactive voice response; NA, not applicable; P, patient participant; RA, research assistant; S, supporter participant.

^a^ Termed *enhanced usual care* in initial protocols.

^b^ Received by participants in both arms (CO-IMPACT and standard care).

### Data Sources and Measures

Patients and supporters were surveyed in person or via telephone at baseline and 12 months. Patient BP and laboratory measurements were collected by study staff at baseline and 12 months. Patient medical record data were obtained from central VA databases. Structured qualitative interviews were conducted with participants in the CO-IMPACT arm at the end of the 12-month intervention. Interviews were transcribed, then analyzed for thematic content using editing analysis style.^28^

### Outcomes

Prespecified primary outcomes were changes from baseline to 12 months in patients’ (1) activation, measured by the PAM-13,^26^ and (2) diabetes-specific cardiac event risk, measured by the UK Prospective Diabetes Study (UKPDS) 5-year risk engine.^29^ The 13 items on the PAM-13 include “I am confident that I can follow through on medical treatments I may need to do at home” and “I am confident I can figure out solutions when new problems arise with my health,” with item response options ranging from “strongly disagree” (1) to “strongly agree” (4). The item response total is transformed into an activation score ranging from 0 to 100 by using the creators’ established algorithm.^26^ Diabetes-specific cardiac risk was chosen as the main physiological outcome to provide a summary measure of multiple risk factors for diabetes complications that may have been modified by improved self-management, including HbA_1c_ levels, SBP, lipid levels, and smoking status. Other components included age, sex, race and ethnicity (ascertained by self-report and collected because the official calculation of the UKPDS score includes a variable for race and ethnicity), and years since diabetes diagnosis.

Prespecified secondary patient outcomes included 12-month changes in glycemic, BP, and cholesterol control; diabetes self-management behaviors (assessed by the Summary of Diabetes Self-care Activities^30^ as the number of days of self-management adherence in the past week; range 0-7 days); diabetes distress (measured by the Problem Areas in Diabetes Scale [PAID]^31^; score range 0-20, with higher scores indicating greater distress); diabetes management self-efficacy (measured by the Stanford Self-efficacy for Diabetes Scale^32^; score range 0-10, with higher scores indicating greater self-efficacy); efficacy in health care participation (measured by the Perceived Efficacy in Patient-Physician Interactions Questionnaire^33^; score range 5-25, with higher scores indicating greater participation in interactions between patients and health care professionals); and satisfaction with health care system support for the supporter participants (assessed by a survey item with response options ranging from 1, “strongly disagree,” to 5, “strongly agree”). eTable 1 in Supplement 2 contains details for the measures used.

### Statistical Analysis

Given 80% power and a 2-tailed α of .05, we estimated we needed data from 204 dyads to detect a 4-point difference in PAM-13 score changes between groups and 198 dyads to detect a 2% difference in UKPDS score changes. These estimates assumed a baseline mean (SD) PAM-13 score of 70 (13), with correlation of 0.70 in 2 measurements in 1 year, and a baseline mean (SD) UKPDS score of 18% (12%).

Intent-to-treat analyses were conducted according to baseline dyad assignment. If a supporter withdrew from the study, the patient could continue participating in their assigned arm. If a patient withdrew, their supporter was also withdrawn. Descriptive patient and supporter data at baseline and changes between baseline and 12 months were analyzed for each group. Analyses of outcomes were initially conducted using hierarchical linear models; however, identical results were obtained using linear regression models with continuous change from baseline to 12 months as the dependent variable, adjusted for the baseline value of the outcome, and these are reported for ease of interpretation. Outcomes models were adjusted for variables determining randomization strata and baseline patient use of insulin, known to be independently correlated with patient diabetes outcomes.^34^ Models included only patients with complete data for the relevant outcome. No data from variables included in the outcome regression models was missing from among patients with complete outcome data. Statistical analyses were performed using Stata, version 17 (StataCorp LLC). Two-sided *P* < .05 was considered significant.

## Results

Among the 239 enrolled patients, the mean (SD) age was 60 (8.9) years; 8 (3.3%) were female and 231 (96.7%) were male. Thirty patients (12.6%) were Black or African American, 7 (2.9%) were Native American, 182 (76.2%) were White, 6 (2.5%) identified as more than 1 race, 14 (5.9%) identified as other race (specific races within this category were not provided) or did not answer, and 14 (5.9%) were Latino or Latina ethnicity. Fifty-six patients (23.4%) had completed college (Table 2). The mean (SD) time since diabetes diagnosis was 12.5 (9.0) years, and 142 patients (59.4%) used insulin at baseline. The mean (SD) baseline HbA_1c_ level was 8.5% (1.6%), and the mean (SD) SBP was 140.2 mm Hg (18.4 mm Hg). At baseline, patient activation (mean [SD] PAM-13 score, 61.3 [23.0]) and diabetes self-efficacy (mean [SD] Stanford Self-efficacy for Diabetes Scale score, 8.2 [1.7]) were comparatively high, and diabetes distress was comparatively low (mean [SD] PAID score, 5.9 [5.2]).

**Table 2.** Participant Baseline Characteristics

Characteristic	Participants, No. (%)		
	CO-IMPACT intervention (n = 123)	Standard care (n = 116)	Overall (n = 239)
**Patient**			
Age at baseline, mean (SD), y	60 (8.4)	60 (9.4)	60 (8.9)
Sex			
Female	6 (4.9)	2 (1.7)	8 (3.3)
Male	117 (95.1)	114 (98.3)	231 (96.7)
Race			
Black or African American	12 (9.8)	18 (15.5)	30 (12.6)
Native American^a^	6 (4.9)	1 (0.9)	7 (2.9)
White	90 (73.2)	92 (79.3)	182 (76.2)
More than 1 race	6 (4.9)	0	6 (2.5)
Other or not reported^b^	9 (7.3)	5 (4.3)	14 (5.9)
Latino or Latina ethnicity	10 (8.1)	4 (3.4)	14 (5.9)
Completed college	29 (23.6)	27 (23.3)	56 (23.4)
Annual income, $^c^			
<30 000	35 (29.2)	39 (34.2)	74 (31.6)
30 000 to <50 000	31 (25.8)	34 (29.8)	65 (27.8)
50 000 to <75 000	31 (25.8)	17 (14.9)	48 (20.5)
≥75 000	23 (19.2)	24 (21.1)	47 (20.1)
Low health literacy^d^	57 (46.3)	52 (44.8)	109 (45.6)
Used insulin at baseline	78 (63.4)	64 (55.2)	142 (59.4)
Time since diabetes diagnosis, mean (SD), y	13.7 (10.0)	11.2 (7.7)	12.5 (9.0)
Study site was hospital-based, vs community clinic	78 (63.4)	87 (75.0)	165 (69.0)
PAM-13 score, mean (SD)^e^	62.8 (11.2)	59.8 (12.6)	61.3 (12.0)
UKPDS 5-y cardiac event risk, mean (SD), %	14.7 (10.0)	13.6 (9.9)	14.2 (9.9)
HbA_1c_ level, mean (SD), %	8.4 (1.5)	8.6 (1.8)	8.5 (1.6)
Systolic blood pressure, mean (SD), mm Hg	141.0 (18.3)	139.3 (18.5)	140.2 (18.4)
Total cholesterol to HDL ratio, mean (SD)	4.7 (1.6)	4.5 (1.5)	4.6 (1.5)
Current smoker	20 (16.3)	16 (13.8)	36 (15.1)
Diabetes distress (PAID), mean (SD)^f^	5.6 (5.2)	6.2 (5.2)	5.9 (5.2)
Diabetes self-efficacy, mean (SD)^g^	8.5 (1.4)	7.9 (1.9)	8.2 (1.7)
Healthcare participation (PEPPI), mean (SD)^h^	21.7 (4.1)	21.2 (4.3)	21.5 (4.2)
Self-management adherence in the past week (SDSCA), mean (SD), d^i^			
Healthy eating	4.1 (2.4)	4.1 (2.3)	4.1 (2.4)
Physical activity	2.9 (2.2)	2.3 (2.0)	2.6 (2.2)
Blood glucose home testing^j^	5.2 (2.2)	4.6 (2.3)	4.9 (2.3)
Blood pressure home testing^k^	2.7 (2.7)	2.1 (2.4)	2.4 (2.5)
Check feet	3.7 (2.3)	3.2 (2.3)	3.5 (2.3)
Take oral medications as prescribed^l^	6.3 (1.3)	6.3 (1.3)	6.3 (1.3)
Take insulin as prescribed^m^	6.4 (1.1)	6.0 (2.0)	6.2 (1.6)
Satisfaction with health care system supporter engagement, mean (SD)^n^	3.6 (1.0)	3.5 (1.0)	3.6 (1.0)
**Supporter**			
Relationship to patient			
Spouse or partner	75 (61.0)	70 (60.3)	145 (60.7)
Friend	25 (20.3)	16 (13.8)	41 (17.2)
Adult child	9 (7.3)	18 (15.5)	27 (11.3)
Other relative	14 (11.4)	12 (10.3)	26 (10.9)
Supporter lives in patient household	86 (69.9)	82 (70.7)	168 (70.3)
Sex			
Female	109 (88.6)	106 (91.4)	215 (90.0)
Male	14 (11.4)	10 (8.6)	24 (10.0)
Race			
Asian	0	1 (0.9)	1 (0.4)
Black or African American	10 (8.1)	16 (13.8)	26 (10.9)
Native American^a^	1 (0.8)	0	1 (0.4)
White	103 (83.7)	92 (79.3)	195 (81.6)
More than 1 race	5 (4.1)	2 (1.7)	7 (2.9)
Other or not reported^b^	4 (3.3)	5 (4.3)	9 (3.8)
Latino or Latina ethnicity	6 (4.9)	0	6 (2.5)
Completed college	25 (20.3)	30 (25.9)	55 (23.0)
Annual income, $^o^			
<30 000	33 (28.7)	34 (31.5)	67 (30.0)
30 000 to <50 000	31 (27.0)	31 (28.7)	62 (27.8)
50 000 to <75 000	29 (25.2)	21 (19.4)	50 (22.4)
≥75 000	22 (19.1)	22 (20.4)	44 (19.7)
Supporter has diabetes^p^	22 (18.2)	24 (20.7)	46 (19.4)

Abbreviations: HbA_1c_, hemoglobin A_1c_; HDL, high-density lipoprotein; PAID, Problem Areas in Diabetes Scale; PAM-13, Patient Activation Measure–13; PEPPI, Perceived Efficacy in Patient-Physician Interactions Questionnaire; SDSCA, Summary of Diabetes Self-care Activities measure; UKPDS, UK Prospective Diabetes Study.

SI conversion factor: To convert percentage of total hemoglobin to proportion of total hemoglobin, multiply by 0.01.

^a^ The Native American category includes participants who identified as Native American, Alaskan Native, Native Hawaiian, or Pacific Islander.

^b^ Participant chose the “other” category (races included in this category were not provided) or did not answer. No patient participants indicated Asian race.

^c^ All but 3 patients in the intervention arm and 2 in the standard-care arm reported income data.

^d^ Assessment of health literacy is explained in eTable 1 in Supplement 2.

^e^ Score range 0-100, with higher scores indicating greater activation.

^f^ Score range 0-20, with higher scores indicating greater distress.

^g^ Stanford Self-efficacy for Diabetes Scale; score range 0-10, with higher scores indicating greater self-efficacy.

^h^ Score range 5-25, with higher scores indicating greater participation in interactions between patients and health care professionals.

^i^ Range, 0-7 days, with higher scores indicating more frequent adherence.

^j^ All but 4 participants in the intervention arm and 6 in the standard-care arm.

^k^ All but 14 participants in the intervention arm and 22 in the standard-care arm (patients who reported not being advised by their clinicians to test glucose or blood pressures at home were not asked self-testing questions).

^l^ All but 14 participants in the intervention arm and 16 in the standard-care arm.

^m^ All but 45 participants in the intervention arm and 51 in the standard-care arm (patients who did not take oral diabetes medications or insulin were not asked these questions).

^n^ All but 3 participants in the intervention arm and 1 in the standard-care arm; score range 1-5, with 1 indicating “strongly disagree” and 5, “strongly agree.”

^o^ All but 8 supporter participants in the intervention arm and 8 in the standard-care arm reported income data.

^p^ All but 2 supporter participants in the intervention arm and 0 in the standard-care arm.

Among the 239 enrolled supporters, 215 were (90.0%) were female and 24 (10.0%) were male. One supporter (0.4%) was Asian, 26 (10.9%) were Black or African American, 1 (0.4%) was Native American, 195 (81.6%) were White, 7 (2.9%) identified as more than 1 race, 9 (3.8%) identified as other race or did not answer, and 6 (2.5%) were Latino or Latina ethnicity. A total of 168 supporters (70.3%) resided with the patient, and 145 (60.7%) were spouses or partners. Forty-six supporters (19.4%) had diabetes.

**Figure 1.**
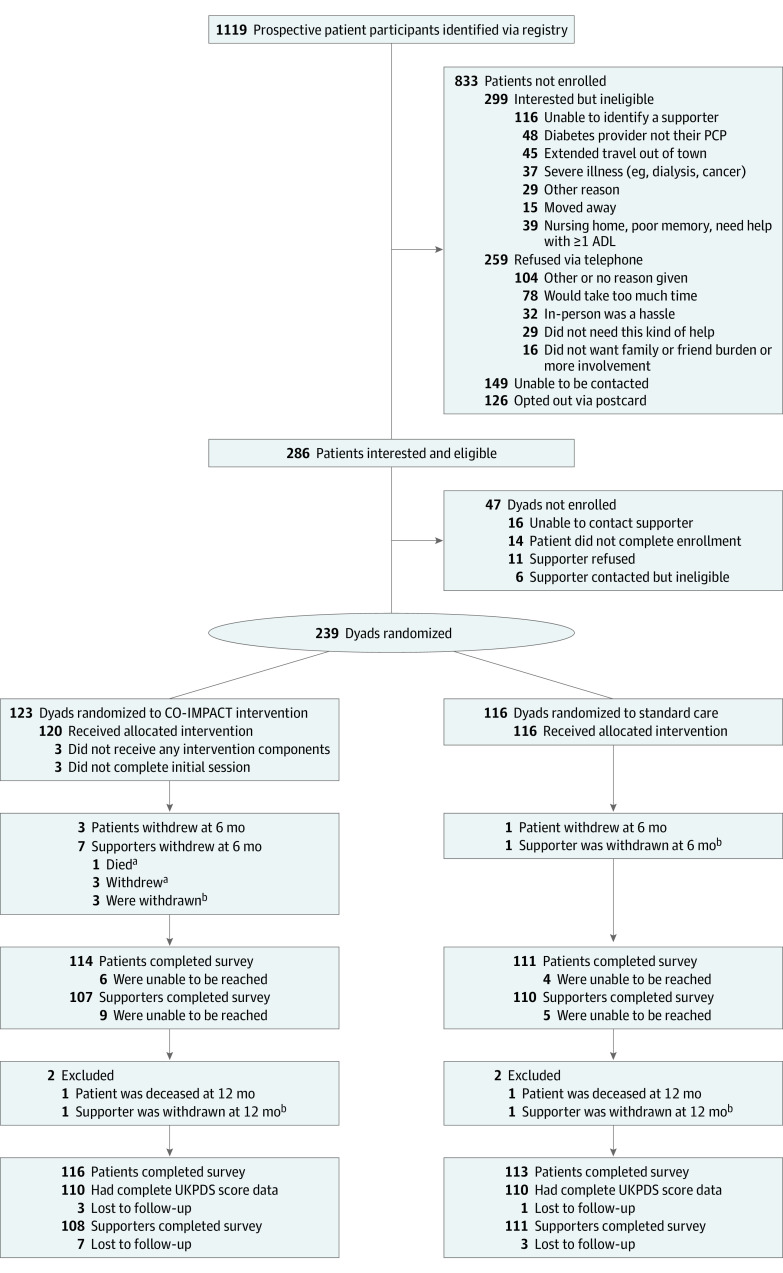
Study Participant Flow Diagram ADL indicates activities of daily living; CO-IMPACT, Caring Others Increasing Engagement in Patient Aligned Care Teams; PCP, primary care physician; and UKPDS, UK Prospective Diabetes Study. ^a^If a supporter died or withdrew, the patient was allowed to continue in the patient-focused parts of their originally assigned intervention. ^b^The supporter was automatically withdrawn if the patient died or withdrew from the study.

Figure 1 shows the study flow diagram, with 1119 recruitment letters resulting in 239 enrolled dyads. Among the 259 recruited individuals who declined to participate and provided reasons, the most common refusal reason was too much time or hassle (110 [42.5%]); only 16 (6.2%) refused due to not wanting increased supporter involvement or burden. However, among the 299 individuals who were interested but ineligible, inability to identify a supporter to participate with them was the most common reason for ineligibility (116 [38.8%]). Among those identified via the registry (eTable 2 in Supplement 2), enrolled patients were 3 years younger on average than those who did not enroll (mean [SD] age, 60 [9.0] vs 63 [7.4] years). Those qualifying via both a high HbA_1c_ level and SBP were more likely to enroll than were those meeting just 1 of these criteria. Four patients (1.7%) withdrew and 2 (0.8%) died during the study period. A total of 229 patients (95.8%) completed 12-month survey assessments, and 220 (92.1%) had complete vital sign and laboratory data.

**Figure 2.**
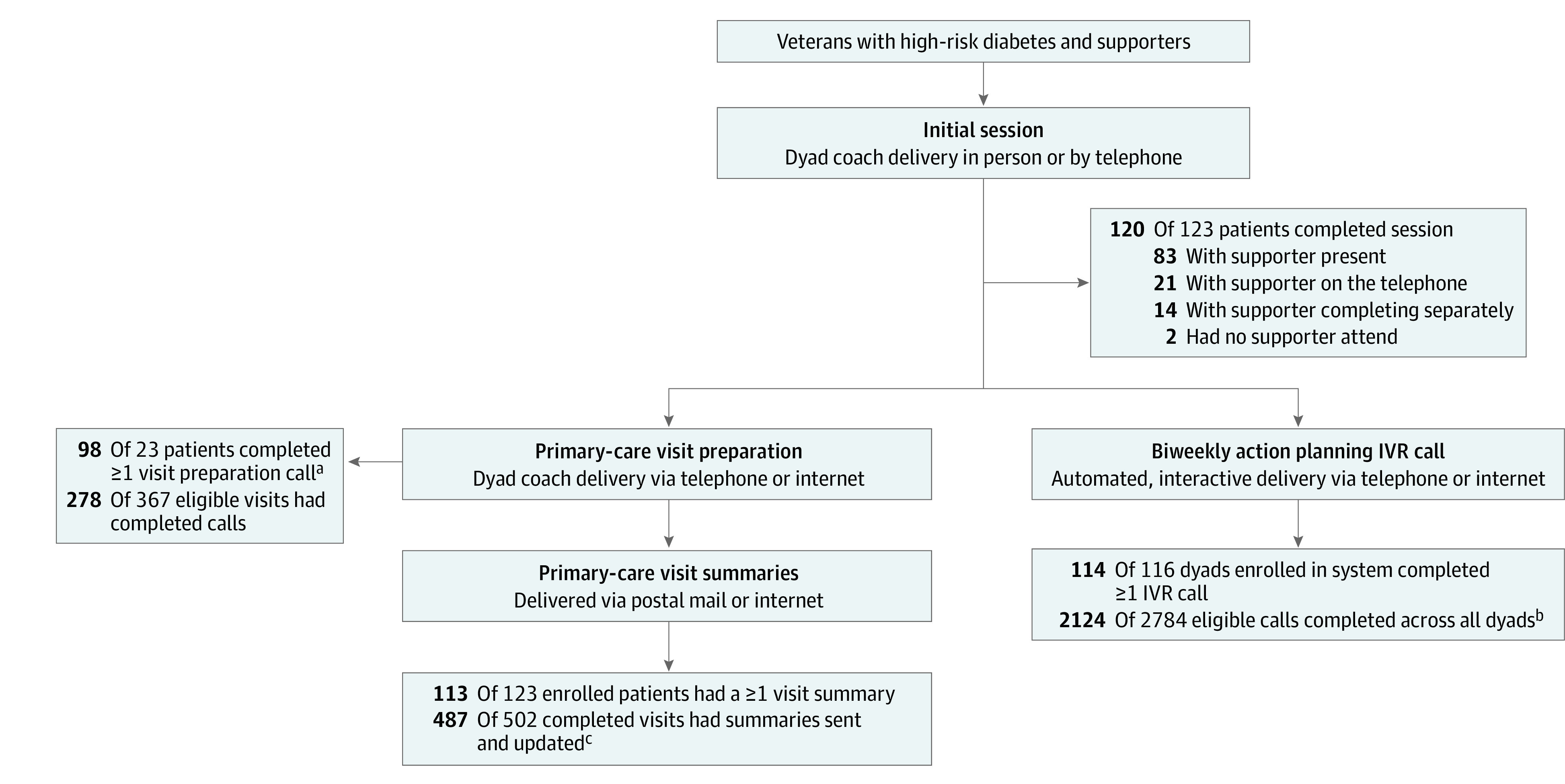
Participation in CO-IMPACT Intervention Components IVR indicates interactive voice response. ^a^Mean (SD) calls per patient over 12 months was 2.4 (1.9). ^b^Mean (SD) calls per dyad over 12 months was 18.6 (6.9). ^c^Mean (SD) summaries per patient over 12 months was 4.2 (2.6).

With regard to CO-IMPACT intervention participation (Figure 2), 120 of 123 CO-IMPACT arm dyads (97.6%) completed the initial session and were enrolled in the IVR system. Of those, 114 (95.0%) completed at least 1 IVR call, with a mean (SD) of 19 (7) completed calls per patient. Ninety-eight dyads (79.7%) completed 1 or more visit preparation calls (mean [SD], 2.4 [1.9] calls per dyad), and 113 (91.9%) received 1 or more visit summaries (mean [SD], 4.2 [2.6] summaries per dyad).

### Patient Outcomes

Unadjusted mean (SD) 12-month PAM-13 score changes (eTable 3 in Supplement 2) were greater for the CO-IMPACT arm than for the standard-care arm (2.9 [9.9] points vs 1.8 [10.8] points). The UKPDS 5-year cardiac event risk score increased slightly on average in the CO-IMPACT group (mean [SD], 0.1% [6.8%]) and decreased slightly in the standard-care group (mean [SD], −0.7% [6.5%]). The HbA_1c_ level and SBP decreased on average in both arms, but HbA_1c_ levels decreased more in the standard-care group, and SBP decreased more in the CO-IMPACT group.

Fully adjusted models (Table 3; eTable 4 in Supplement 2) showed that PAM-13 scores improved more in the CO-IMPACT arm than the standard-care arm (intervention effect, 2.60 points; 95% CI, 0.02-5.18 points; *P* = .048). The UKPDS risk score changes were not statistically significantly different by arm (intervention effect, 1.01 points; 95% CI, −0.74 to 2.77 points; *P* = .26). Self-reported healthy eating (intervention effect, 0.71 d/wk; 95% CI, 0.20-1.22 d/wk; *P* = .007) and diabetes self-efficacy (intervention effect, 0.40 points; 95% CI, 0.09-0.71; *P* = .01) improved more over 12 months in those receiving the intervention compared with standard care. Hemoglobin A_1c_ levels, SBP, diabetes distress, efficacy in health care participation, and other self-management behavior changes did not differ significantly by study arm.

**Table 3.** Adjusted Intervention Effect on 12-Month Changes in Patient Primary and Secondary Outcomes From Multivariable Linear Regression Models^a^

	Adjusted coefficient (95% CI) for intervention from baseline to 12-mo change, vs standard care	Patients included in the model, No.	*P* value
**Prespecified primary outcomes**			
PAM-13 score	2.60 (0.02 to 5.18)	229	.048
UKPDS 5-y cardiac event risk score	1.01 (−0.74 to 2.77)	220	.26
**Prespecified secondary outcomes**			
HbA_1c_ level, %	0.17 (−0.17 to 0.51)	227	.33
Systolic blood pressure, mm Hg	−2.82 (−7.00 to 1.35)	220	.18
Total cholesterol to HDL-C ratio	0.15 (−0.09 to 0.40)	227	.29
Diabetes distress (PAID)	0.12 (−0.95 to 1.19)	225	.83
Diabetes self-efficacy^b^	0.40 (0.09 to 0.71)	228	.01
Efficacy in health care participation (PEPPI)	0.11 (−0.71 to 0.93)	228	.79
Self-management adherence (SDSCA)			
Healthy eating	0.71 (0.20 to 1.22)	229	.007
Physical activity	−0.04 (−0.56 to 0.47)	228	.87
Blood glucose home testing	0.23 (−0.22 to 0.68)	195	.31
Blood pressure home testing	0.47 (−0.25 to 1.19)	137	.20
Check feet	0.26 (−0.22 to 0.75)	228	.29
Medication adherence			
Oral medications	−0.10 (−0.42 to 0.23)	195	.56
Insulin	0.07 (−0.26 to 0.41)	134	.67
Satisfaction with health care system supporter engagement	0.28 (0.07 to 0.49)	218	.009

Abbreviations: HbA_1c_, hemoglobin A_1c_; HDL-C, high-density lipoprotein cholesterol; PAID, Problem Areas in Diabetes Scale; PAM-13, Patient Activation Measure–13; PEPPI, Perceived Efficacy in Patient-Physician Interactions Questionnaire; SDSCA, Summary of Diabetes Self-care Activities Measure; UKPDS, UK Prospective Diabetes Study.

^a^ Each model adjusted for whether the patient and supporter lived together (yes or no), a baseline PAM-13 score higher than 40 vs lower than 40 (total score range, 0-100, with higher scores indicating greater activation), whether the patient used insulin at baseline (yes or no), and the baseline value of the outcome measure. Full model results are included in eTable 4 in Supplement 2.

^b^ Stanford Self-efficacy for Diabetes Scale.

### Satisfaction

Patient satisfaction with health system support for the involvement of supporters improved more in the CO-IMPACT arm (intervention effect, 0.28 points; 95% CI, 0.07-0.49; *P* = .009). Most patients and supporters in the CO-IMPACT arm rated each component as helping a great deal or somewhat (eTable 5 in Supplement 2); the initial coaching session received the highest rating (189 of 215 patients and supporters [87.9%] said it helped a great deal or somewhat) and visit preparation calls received the lowest (only patients were asked this question; 76 of 109 [69.7%] said the calls helped a great deal or somewhat). Eighteen of 105 supporters (17.1%) did not recall receiving email summaries of patient IVR entries. Most participants (108 of 111 patients [97.3%]; 98 of 105 supporters [93.3%]) said they would definitely or probably recommend CO-IMPACT to others like them, and 87 of 105 supporters (82.9%) felt they and the patient improved how they worked together to manage diabetes.

### Qualitative Analyses

Thematic analysis of CO-IMPACT participant interview transcripts indicated ways that patients and supporters worked together to increase goal setting and other activated patient behaviors (eTable 6 in Supplement 2). Participants gave multiple examples of supporters learning better ways to help patients, of the dyad increasing how often they discussed the patient’s diabetes, and of ways the dyad used team-based approaches, sometimes identified via “we-talk.”^35,36^ Although there were many positive comments about the IVR system, there were also reasons for dissatisfaction, including repetitiveness, patients not knowing that supporters received call summaries, and wariness to reveal “bad” results. Some patient and supporter participants desired more interaction with human coaches.

## Discussion

In this randomized clinical trial of a dyadic intervention to engage adults with diabetes along with a family supporter in diabetes care, patients receiving the CO-IMPACT intervention had a greater improvement in activation (PAM-13 score), 1 of 2 prespecified primary outcomes. The clinical significance of the PAM-13 score change of the magnitude we observed (a 2.6-point improvement) is supported by multiple studies showing associations between this level of PAM-13 score change and significant, positive changes in patient behaviors and health outcomes.^37,38,39^ The CO-IMPACT patients’ diabetes self-efficacy and healthy eating behavior also improved compared with those receiving standard care. Improvements in self-efficacy of the magnitude we observed have been associated with mediating improvements in diabetes clinical outcomes in patient-only interventions.^40,41^ However, in this trial, improvements in cardiac risk score and individual physiological outcomes were similar in both arms.

Increases in patient activation and the related concept of self-efficacy are key patient-centered diabetes outcomes. Moreover, each provides an essential foundation for patients to make healthy behavior changes and engage in medical care. In other studies, these measures have been associated with improvements in health behaviors, glycemic control, and hospitalization rates among adults with diabetes.^38,42,43^ The difference in PAM-13 score changes we observed between study arms had a *P* value slightly less than .05 and are unlikely to be spurious given the supporting finding of significant differences in self-efficacy change. These improvements were achieved despite using no in-person clinician time and only brief phone or digital interactions with participants. In separate analyses from this trial, supporters in the CO-IMPACT arm experienced significantly increased involvement in several patient diabetes-management tasks and increased use of positive, autonomy-supportive communication compared with those receiving standard care.^44^

Several factors may explain the lack of significant impact of CO-IMPACT on physiological measures beyond improvements observed in the standard care group. Our intervention was primarily delivered via automated telephone calls, and supporters received less direct interaction than did patients after the initial coaching session. Some supporters indicated in qualitative interviews that they did not receive automated messages and desired more direct contact with coaches. Future studies should test more intensive and direct follow-up with supporters. Our qualitative data indicate that studies using automated systems with patients and supporters may be more engaging by varying message content and intensity, providing periodic notifications that the other member of the dyad is receiving summary messages, and reminders reminding participants of the benefits of discussing supportive follow-up plans, particularly when diabetes care is not going well. In addition, patients received health care in VA settings with high documented quality of primary and diabetes care^45,46^ and extensive resources to support diabetes management. Interventions to increase family support may benefit patients more in settings with fewer diabetes support resources.

### Strengths and Limitations

This study has strengths. It was highly successful in recruiting, engaging, and retaining adult patient-supporter dyads, which has been challenging in prior research.^47,48^ A key strategy was accommodating supporters who lived with or apart from the patient and those who were related to the patient. Many health care systems are trying to increase their engagement of patients’ caregivers, as promoted by recent legislation.^49,50^ Concurrent with the time frame of this study, the VA has prioritized expanding programs to engage and support patients’ caregivers.^51,52^ Among CO-IMPACT patients, satisfaction with health care system support for their supporter’s involvement in care increased significantly more than among patients in standard care, and most supporters felt CO-IMPACT improved their ability to help patients. Possible factors in this success, supported by our qualitative findings, include the intervention’s focus on a positive, patient-supporter teamwork approach, increased sharing of health care information with supporters, and supporter encouragement of patients’ engagement in health care.

This study also has limitations. The generalizability of findings is limited because most participants were non-Latino White men. We only enrolled patients who could identify a family or friend supporter who was willing and able to be involved in patient care. However, this mirrors the selection process for existing clinical programs that engage patients’ existing informal supporters or caregivers. For patients without family supporters, peer support or more intensive nurse care management may be more appropriate. The study was conducted in the VA health care system, with integrated payment and widespread implementation of care based on patient-centered medical home teams. However, patient-centered medical home teams are being increasingly implemented, and our intervention models how supporters could be included in team-based care.^53^ Interactive voice response systems are not yet commonly available in health care systems. However, automated monitoring programs, by text, app, or telephone, are increasingly common and provide unique opportunities for family supporters to monitor and respond to patient health information asynchronously or from a distance. Patients in our trial started with high baseline activation and self-efficacy scores; patients with lower levels of activation and efficacy may experience different outcomes. We evaluated 2 primary outcomes and several prespecified secondary outcomes without adjusting *P* value thresholds for multiple testing, as per our prespecified statistical analysis plan. The clinical significance of results with confidence intervals should be interpreted with this in mind. In addition, using both HbA_1c_ levels and SBP for eligibility may have limited our ability to detect change in either separate outcome. However, our intervention sessions and choice of complication risk as the primary physiological outcome reflect that both glycemic and BP control are associated with lower risk of diabetes complications.^54,55^ Our success in recruiting patients who met both criteria suggests that diabetes programs may be more appealing to participants if multiple risks for complications are addressed.

## Conclusions

The CO-IMPACT dyadic intervention successfully engaged family supporters in adults’ diabetes care in ways participants found valuable and led to improved patient activation and self-efficacy in managing diabetes. Our findings from this randomized clinical trial indicate that increasing family supporters’ engagement in the care of adults with diabetes is feasible and may improve key behavioral determinants. Future studies should investigate whether interacting more directly with patients’ supporters and targeting patients with higher needs for support would help translate the observed benefits into physiological improvements.

## Notes

Supplement 1.
Trial Protocol

Supplement 2. **eTable 1.** Key Study Measure Details
eReferences
**eTable 2.** Predictors of Enrollment Among the 1119 Patients With Diabetes Sent a Recruitment Letter **eTable 3.** Unadjusted Changes in Patient Outcomes by Study Arm, Baseline to 12 Months **eTable 4.** Intervention Effect on Baseline–to–12-Month Changes in Patient Outcomes From Multivariable Linear Regression, Full Model Results **eTable 5.** Patient and Supporter Ratings of Experience With CO-IMPACT Intervention Components **eTable 6.** Qualitative Themes Among CO-IMPACT Intervention Arm Patient and Supporter Participant Comments **eFigure.** CO-IMPACT IVR Topic Flow Chart **eAppendix.** CO-IMPACT Intervention Materials Toolkit Location

Supplement 3.
Data Sharing Statement
