# Supplementary material for: Effectiveness of a Health Coaching Intervention for Patient-Family Dyads to Improve Outcomes Among Adults With Diabetes: A Randomized Clinical Trial
Source: JAMA Netw Open. 2022 Nov 14;5(11):e2237960. doi: 10.1001/jamanetworkopen.2022.37960 (PMC9664266; doi:10.1001/jamanetworkopen.2022.37960)
Supplement: Supplement 1. — Trial Protocol [file jamanetwopen-e2237960-s001.pdf]

Study Protocol and Analysis Plan

Effects of Engaging Family Supporters of Adult Patients with Diabetes on Patient Activation, Self-Management, and Clinical Outcomes: A Randomized Clinical Trial

Rosland AM, Piette JD, Trivedi R, Lee AA, Stoll S, Youk A, Obrosky DS, Deverts D, Kerr EA, Heisler ME

Initial IRB-Approved Protocol……………………………………………………..........................……….....Page 2

Initial Statistical Analysis Plan…………………………………………………………………………Page 20

Final IRB-Approved Protocol…………………………………….………..........................…..…....Page 32

Final Statistical Analysis Plan…………………………………….……………………………….Page 54

STUDY PROTOCOL

Title: Engaging Veterans and Family Supporters in PACT to Improve Diabetes Management

Funding Source: VA Health Services Research and Development

Principal Investigator: Ann-Marie Rosland, MD MS

Protocol Version: IRB Approved July 18, 2014

XI. List of Terms and Abbreviations

[REDACTED]VA-[redacted] VA
ADL – Activities of Daily Living
AE- Adverse Events
BMI – Body Mass Index
BP – Blood pressure
Care Partner – Unpaid family member or friend who is involved in the patient's health care
CBOC – Community Based Outpatient Clinic
CO-IMPACT - Caring Others Increasing EngageMent in PACT intervention
CPRS – Computerized Patient Record System
DEC – Dyad Engagement Coach
ED – Emergency Department
HbA1C – Glycated hemoglobin (hemoglobin A1c)
HIPAA - Health Insurance Portability and Accountability Act
IVR- Interactive Voice Response
LDL – low-density lipoprotein
PACE - Patient and Caregiver Experiences with Diabetes Study
PACT- Patient Aligned Care Teams
Patient Partner – Patient participants who are paired with a Care Partner
PCP – Primary Care Physician
PHI - Protected Health Information
RCT – Randomized Controlled Trial
SAS - Statistical Analysis Software
SBP – systolic blood pressure
SD - Standard Deviation
UKPDS – United Kingdom Prospective Diabetes Study
VA – Veterans Affairs
[REDACTED]HS - VA [redacted] Healthcare System
VHA – Veterans Health Administration

1. Abstract

BACKGROUND: Veterans with diabetes must control cardiovascular risk factors in order to prevent disabling and life-threatening complications. The VA PACT initiative seeks to provide patients comprehensive support for following diabetes care regimens, but Veterans but effectively engage in and navigate care to obtain the most benefit from PACT. One relatively untapped resource for supporting engagement in PACT is patients' family and friends ("Care Partners").

OBJECTIVES: The *overall objective* of this randomized trial is to test a strategy to strengthen the capacity of supporters to help patients with high-risk diabetes engage in PACT care and successfully enact care plans. The *central hypothesis* is that providing health care engagement tools to both Care Partners and patients will increase patient activation and improve management of diabetes complication risks.
RESEARCH PLAN: This will be a randomized controlled trial evaluating an intervention (Caring Others Increasing EngageMent in PACT, or CO-IMPACT) designed to structure and facilitate Care Partner involvement in PACT so that patients can become more actively engaged in PACT care, and improve their diabetes treatment processes and outcomes.

METHODS: 220 patients with diabetes receiving PACT primary care who 1) are at high risk for diabetes complications due to hyperglycemia OR high blood pressure and 2) have a Care Partner involved in their care will be recruited along with their Care Partner. Patient-supporter dyads will be randomized to the CO-IMPACT intervention or usual PACT care for high-risk diabetes, for 12 months. The CO-IMPACT protocol provides patient-supporter dyads: one coaching session on action planning, communicating with providers, navigation skills and support skills; preparation by phone before patients' primary care visits; after-visit summaries by mail; and biweekly automated phone calls to prompt action on new patient health concerns. CO-IMPACT builds on medical record-integrated patient activation tools in the PACT toolkit and is designed to be implementable within existing PACT nurse encounters. Primary outcomes for this study include a validated measure of patient activation (Patient Activation Measure-13) and a cardiac event 5-year risk score designed for patients with diabetes (UKPDS Risk Engine). Secondary outcomes include patients' self-efficacy for diabetes self-care; diabetes self-management behaviors including medication adherence; diabetes distress; and glycemic and blood pressure control. Measures among supporters will include supporter activation, use of effective support techniques, distress about patient's diabetes care, and Care Partner burden. We will also measure patient-supporter and patient-provider relationship quality, patient safety (e.g. hypoglycemia), and utilization. We will measure potential moderators of intervention effect, such as patient health literacy level, and facilitators and barriers to wider implementation among participants and staff.

2. Study Personnel

Principal Investigator/Study Chair:
Ann-Marie Rosland, MD, MS
Research Investigator, VA Ann Arbor Healthcare System
Address: NCRC (152), 2800 Plymouth Rd., Bldg 16, 3rd Floor, Ann Arbor, MI 48105
Phone: (734) 845-3504
Email: ann-marie.rosland@va.gov

Co-Investigators:
Michele Heisler, MD, MPA
Research Investigator, VA Ann Arbor Healthcare System
Address: NCRC (152), 2800 Plymouth Rd., Bldg 16, 3rd Floor, Ann Arbor, MI 48105
Email: mheisler@umich.edu

Eve Kerr, MD, MPH
Research Investigator, VA Ann Arbor Healthcare System;
Address: NCRC (152), 2800 Plymouth Rd., Bldg 16, 3rd Floor, Ann Arbor, MI 48105
Email: Eve.Kerr@va.gov

John Piette, PhD, MSc
Research Investigator, VA Ann Arbor Healthcare System
Address: NCRC (152), 2800 Plymouth Rd., Bldg 16, 3rd Floor, Ann Arbor, MI 48105
Email: jpiette@umich.edu

Ranak Trivedi, PhD, MA, MS
Research Investigator, VA Palo Alto Healthcare System
Address: VA Palo Alto Health Care System, Menlo Park Division, 795 Willow Road, Menlo Park, CA 94025
Email: Ranak.Trivedi@va.gov

Study Staff:
Fatima Makki
Project Manager, Center for Clinical Management Research
Address: NCRC (152), 2800 Plymouth Rd., Bldg 16, 3rd Floor, Ann Arbor, MI 48105
Email: Fatima.makki@va.gov

Jenny Davis
Data Analyst, Center for Clinical Management Research
Address: NCRC (152), 2800 Plymouth Rd., Bldg 16, 3rd Floor, Ann Arbor, MI 48105
Email: Jennifer.davis@va.gov

Wyndy Wiitala
Biostatistician, Center for Clinical Management Research
Address: NCRC (152), 2800 Plymouth Rd., Bldg 16, 3rd Floor, Ann Arbor, MI 48105
Email: wyndy.wiitala@va.gov

3. Rationale

Despite system wide advances in diabetes quality of care, over 30% of VHA patients with diabetes have high blood pressure, hyperglycemia, and hyperlipidemia and thus are at high-risk for disabling diabetes complications. To reduce diabetes complications, these 'high-risk' veterans are advised to follow treatment regimens that are complicated and often difficult to follow. PACT (Patient-Aligned Care Teams) seeks to provide patients comprehensive, team-based support for following diabetes care regimens. PACT's success, however, hinges on its ability to effectively engage patients in care. It is widely appreciated that patients who are more engaged in their health care have better health outcomes.[1] To fully engage in PACT, veterans must effectively communicate with multiple PACT team members and proficiently navigate the health care system. High-risk patients, with more complex care needs, often need more support to engage in what PACT has to offer.

One relatively untapped resource for this support is patients' family and friends. Three out of four adults with diabetes reach out to an unpaid family member or friend (a Care Partner) for ongoing help with diabetes management.[2,3] Half regularly bring a Care Partner to their medical appointments.[4,5] Chronically ill patients with Care Partners have better self-management and long-term health outcomes.[6–9] However, while PACT emphasizes the importance of family members as part of the care team, <u>PACT does not have formal mechanisms to involve Care Partners in PACT care</u>. This is unfortunate as these supporters could play a crucial role in helping patients effectively engage in PACT care. Our preliminary work shows that 25-50% of Care Partners already regularly talk with PACT providers on the phone, try to help patients prepare for PACT appointments, and try to help patients carry out plans made at their medical visit. However, studies indicate that Care Partners are currently less effective at influencing patients' medical self-management tasks (e.g., medication adherence or blood glucose monitoring) than healthy lifestyles (e.g., healthy eating).[10] Care Partners tell us they need more information on patient's medical care plans, clear channels for communicating with PACT team members, and information on navigating PACT resources.

4. Research Problem or Question

Our <u>*long-term goal*</u> is to provide VA clinical teams with evidence-based structured approaches to communicating with Care Partners that improve patient health outcomes and satisfaction with care. The <u>*objective*</u> of this randomized trial is to test a strategy to strengthen the capacity of supporters to help patients with high-risk diabetes engage in PACT care and successfully enact care plans. Our <u>*central hypothesis*</u> is that providing health care engagement tools to both Care Partners and patients will increase patient activation and improve management of diabetes complication risks.

5. Specific Aims and Primary Measures

The study will address the following <u>*specific aims*</u>:

1) Determine the effect of the CO-IMPACT intervention on engagement in treatment and health behaviors among patients at high-risk for diabetes complications. *We hypothesize that CO-IMPACT will significantly increase patient activation, as measured by the PAM-13, compared to usual PACT care.*

2) Determine the effect of the CO-IMPACT intervention on health risks among patients at high-risk for diabetes complications**.** *We hypothesize that CO-IMPACT will significantly decrease patients' 5-year cardiovascular event risk, as measured by the UKPDS cardiac risk score (which includes HbA1C, lipid levels and blood pressure), compared to usual PACT care.*

3) Evaluate how the characteristics of patients, family supporters, and their relationships mediate and moderate the effects of CO-IMPACT. *We hypothesize that higher levels of family supporter participation in CO-IMPACT will lead to greater improvements in patient activation and patient cardiac risk.*

6. Background

<u>Many VHA Patients With Diabetes Are At High Risk For Diabetes Complications:</u>

Twenty-five percent of VHA patients have diabetes, representing about 1.5 million Veterans. While the quality of diabetes care is high when measured by processes such as HbA1c monitoring and lipid testing, 20-30% of VHA

diabetes patients have poor glycemic control (HbA1c >8%), poor blood pressure control (>140/90), or high lipid levels (LDL >130 mg/dl).

Increasing Patient Activation Can Improve Diabetes Management:

"Activated" patients are those who have the "skills and confidence to become actively engaged in their health and healthcare."[11] Activation includes the ability to share in decision-making with health care providers, monitor and self-manage symptoms, and access care in an appropriate and timely way. Patient activation encompasses several more specific health behavior concepts, including locus of control and self-efficacy for executing self-managing behaviors.[12] The main result of patient activation is patient engagement, or "actions that people take for their health and to benefit from care"[13]

Highly activated patients have better health behaviors (including adherence to medications, regular self-monitoring at home, physical activity, and healthy eating) and health outcomes (including lower BMI, HbA1C, blood pressure, and cholesterol).[1] Increases in activation over time are linked to improvements in similar health behaviors and outcomes.[14] Less activated patients are unengaged in their care, delay care seeking and have poorly coordinated outpatient follow-up, including in the VHA.[15–17] Less activated patients also have higher rates of hospitalizations and ED visits,[1] and higher costs of care.[11]

Intervention studies have shown that coaching or prompting patients to ask questions immediately prior to a medical visit can increase patient activation during and just after the visit.[18,19] Most of these studies have been done in oncology clinics, but one RCT of pre-visit coaching to increase diabetes patients' information gathering led to improved glycemic control and less reported functional limitations compared to control patients.[20] How best to increase global patient activation among patients with chronic illness is still uncertain.

Full Patient Engagement in PACT Diabetes Management Requires High Activation:

The VA Patient Aligned Care Teams (PACT) model for primary care is designed to provide multiple, coordinated mechanisms for supporting patients with complex chronic disease. These include visits with nurse care managers and clinical pharmacists that complement primary care provider (PCP) visits, telephone visits between in-person visits, health psychology programs to support self-management behavior change, group diabetes education, the MOVE! weight management program, and telehealth monitoring. To make the most of this complex array of new services, patients must identify resources that can best meet their needs, make appointments or enroll in programs, actively participate, implement care plan changes, and maintain ongoing communication with clinical teams. Uptake of PACT chronic disease management programs has been slow,[21] and it is uncertain what approach is best to help complex patients obtain the full benefit of PACT chronic disease care. *In our CO-IMPACT (Caring Others Increasing EngageMent in PACT) intervention, we will mobilize patients' family members and friends to help increase patient engagement in PACT care.*

Most VA Patients with Diabetes Have Family Members or Friends Who Are Involved in Their Health Care:

As many as 75% of VA patients with diabetes have a family member or friend who is regularly involved in their diabetes care (a 'Care Partner').[2,3] These supporters assist patients in engaging in activities directly related to successful diabetes management, including medication management and adherence, tracking home glucose and blood pressure measurements, maintaining a healthy eating plan, and being physically active.[2,3,22] Care Partners often help patients make key decisions about their diabetes management, such as how to address medication side effects.[23] Typically, 50-60% of Care Partners are spouses, and most of the rest are family members who do not live with the patient (such as adult children).[2,24,25]

Care Partners Can Affect Chronic Disease Management and Outcomes:

Family and friend support can lead to better glycemic control and lower mortality among patients with diabetes.[6] In other chronic conditions that require significant self-management, such as cardiac disease and heart failure, social support is linked to lower rates of recurrent cardiac events and hospitalizations.[7,8] There is strong evidence that social support acts on chronic disease outcomes largely through improved patient self-management behaviors.[10]

There is little direct evidence that Care Partner engagement can increase overall patient activation, but we have several reasons to hypothesize that this is the case. There are very strong links between social support and improved patient self-efficacy for self-care,[27–30] a concept closely related to patient activation. Higher social support is linked to activated self-management behaviors, such as increased self-monitoring.[31,32] When supporters accompany patients

to medical visits, patients exhibit more activated behavior, including increased participation in decision making with providers.[4,5,25] In one pre-visit preparation intervention delivered to patients with cancer and their visit companions, both patient and companion question asking increased.[33] We found in prior work that patients participating with a Care Partner in an interactive voice response self-management intervention were more engaged in the intervention than those who participated alone.[34,35]

Previous interventions aiming to leverage family support to improve disease management have generally engaged supporters in patients' day-to-day health management through counseling or coaching.[36,37] Such interventions have demonstrated improvements in dietary behavior among heart failure patients,[38] and physical activity among obese patients.[39] However no published interventions or known clinical programs have focused on helping Care Partners boost chronically ill patients' engagement in clinical care and medical self-care (i.e. medication adherence).

Significance of Proposed Research:

Veterans with high-risk diabetes are highly vulnerable to disabling complications and death. These patients often remain at high risk for poor outcomes and frequently use emergency care. Innovative and sustainable approaches that increase engagement of patients' and their supporters in primary care could improve patients' risk factor control, and reduce emergency utilization and morbidity.

The VA has recently expanded its commitment to engaging family caregivers in medical care. The Caregivers and Veterans Health Services Act of 2010 provides Veterans' caregivers with substantial support through several means, including increased training and financial support; a telephone support line; a VA website with caregiving tools and resources, and full-time Caregiving Program Coordinators at each VA facility nationwide.

An overarching goal of the nationwide VA PACT initiative is to engage the patient, and all those helping to care for the patient, in a coordinated, team-based approach.[40] The PACT model specifically includes family members as part of the care team. Structured and implementable approaches to identifying Care Partners and including them in the flow of health care information are needed for PACT to achieve this goal. If successful, this study will produce a scalable protocol that can be used by VA PACT teams across the VHA to engage Veterans' Care Partners and caregivers in diabetes care. The lessons learned in this study can be used to evaluate and enhance Care Partner and caregiver engagement for patients with other complex conditions, or patients in vulnerable situations such as transitions from hospital to home.

7. Preliminary Data

[redacted] VA Patients with High-Risk Diabetes Have Involved Care Partners: In our prior studies, including our preliminary VA observational studies of Patient and Caregiver Experiences (PACE) with Veterans with high-risk diabetes and their Care Partners, we found that 40% of out-of-home supporters live within 20 miles of the patient's home, and 78% talk with the patient by phone at least weekly.[24] Prior research, including our own, has shown that chronically ill patients with low health literacy, multiple comorbidities, and comorbid depression involve Care Partners in their care more often.[4,5,26]

Our prior studies also indicate that Care Partners are highly involved in patients' interactions with the health care system. About half of patients with diabetes are regularly accompanied by a supporter into the exam room for primary care visits,[4,5] and 25% have had a supporter talk on the phone with their clinician in the last year.[4] Importantly, (Table 1) we found that Care Partners often help patients prepare questions before visits, assist patients in processing visit information and plans ('debriefing'), and help patients navigate VA services such as pharmacy fills and diabetes class enrollment. *Thus, there is significant potential to increase patient engagement in PACT care by enhancing the effectiveness of these interactions among Care Partners, patients, and PACT team members at key points in medical care.*

Care Partners are Limited By Lack of Patient-Specific and VA-Specific Information, and Structured Support Opportunities: In our national survey of 760 family supporters of patients with chronic disease,[23] and in our 12 VA PACE supporter interviews, supporters reported feeling limited by a lack of patient-specific information, such as changes in medication regimens or test results, as well as a lack of health system-specific information, such as the roles of PACT teamlet members or available diabetes programs. Supporters also face significant challenges when helping patients prepare for, and debrief after, clinical visits. For example, as shown in Table 1, patients often do not bring written questions for the doctor, and many are not confident they are reporting accurate visit information back

to their supporter. Twenty-eight percent of supporters reported that their patient-partner regularly discusses being confused about health care provider instructions.[23] In PACE interviews, many supporters requested printed summaries after patient visits with a clear way to follow-up with questions. Importantly, few patients (9%) in PACE surveys felt that privacy should be a barrier to information sharing between supporters and patients' clinicians. However in interviews, several supporters reported feeling intimidated by perceived complexity in VA primary care clinic structure and privacy rules.
Supporter effectiveness could also be boosted through more structured and action-oriented between-visit discussions with patients. In our national Care Partner survey, we found that supporters discuss health with their patient-partners almost every time they talk, but approximately 30% were unsure what questions to ask or what advice to give about diabetes. Supporters can make the most of these discussions when they have patient-specific information and when they use evidence-based support techniques,[41] such as positive and autonomy-supportive statements, and collaborative action planning and coping.

Pilot of CO-IMPACT Shows Feasibility and Perceived Benefit: In preparation for the current trial, we developed the CO-IMPACT intervention protocol, patient and supporter materials, and assessment instruments, and delivered CO-IMPACT (see Section 8, Table 1) to 19 patient-supporter dyads over a 4-month period. Patient participants were recruited from a [REDACTED]HS registry of patients with high-risk diabetes with similar criteria to section D3. 18/19 patients were men, with a mean age of 66 years (range 47-89). Patients chose a Care Partner to participate with them, who was assessed for eligibility as described in D4. Eighteen of 19 supporters were women; mean age of supporters was 54 years (range 22-71). Most supporters were spouses (N 11) and lived with the patient. The other 8 supporters did not live with the patient (7/8 lived ≤20 miles apart) and included 3 daughters, 1 son, 3 friends, and 1 other family member. All patient-supporter dyads completed an initial session with a Dyad Engagement Coach(DEC). Among the 19 patients, 21 pre-visit preparation phone calls were completed (out of 25 eligible visits), and 25 after-visit summaries were mailed to dyads. Patients completed 82% of attempted weekly automated IVR telephone assessments, with 18/19 patients continuing to complete IVR calls for the entire 4 months. At follow-up, 95% of patients and 89% of supporters said they were satisfied with the program. 84% of patients felt CO-IMPACT helped them more effectively manage diabetes, and 84% of supporters felt the program helped them more effectively support the patient's health care. 100% of patients would recommend the program to another Veteran. In post-intervention interviews, both patients and supporters reported that CO-IMPACT was helpful in promoting patient engagement and changing patient-supporter communication about care: "She [supporter] reminded me [patient] to call my doctor with problems", "He [patient] brought our questions with him to his appointment and asked them to the doctor and nurse", "I [supporter] am more aware of what questions to ask him [patient] and what to say about diabetes". Extensive interviews with participants and their PACT teamlets have informed intervention refinement.

8. Overview of Study Design:

This will be a randomized controlled trial evaluating an intervention designed to activate dyads of Care Partners and Veterans with diabetes (CO-IMPACT). 220 patients with diabetes receiving PACT primary care who are at high risk for diabetes complications and who have a Care Partner involved in their care will be recruited along with their Care Partner.

The overarching goal of this intervention is to structure and facilitate Care Partner involvement in PACT so that patients can become more actively engaged in PACT care, and improve their diabetes treatment processes and outcomes. CO-IMPACT will address key limitations to supporter effectiveness by providing supporters with information about their patient-partner's health status and treatment plan, ways to effectively identify and engage in PACT-related services, structured pre, post, and between visit information that can improve supporter-patient discussions about diabetes plans, and guidance to supporters on evidence-based communication techniques. Patient-supporter dyads will be identified via a [REDACTED]HS registry of patients with high-risk diabetes and randomized to CO-IMPACT or usual PACT care. Outcomes will be measured at baseline and 12 months post-enrollment via patient and supporter surveys, and patient laboratory tests, vital signs, and medical records. Implementation barriers and facilitators will be assessed in qualitative interviews with patients, supporters, and PACT staff. The below chart is a depiction of the overall study design.

Table 1: Study Design

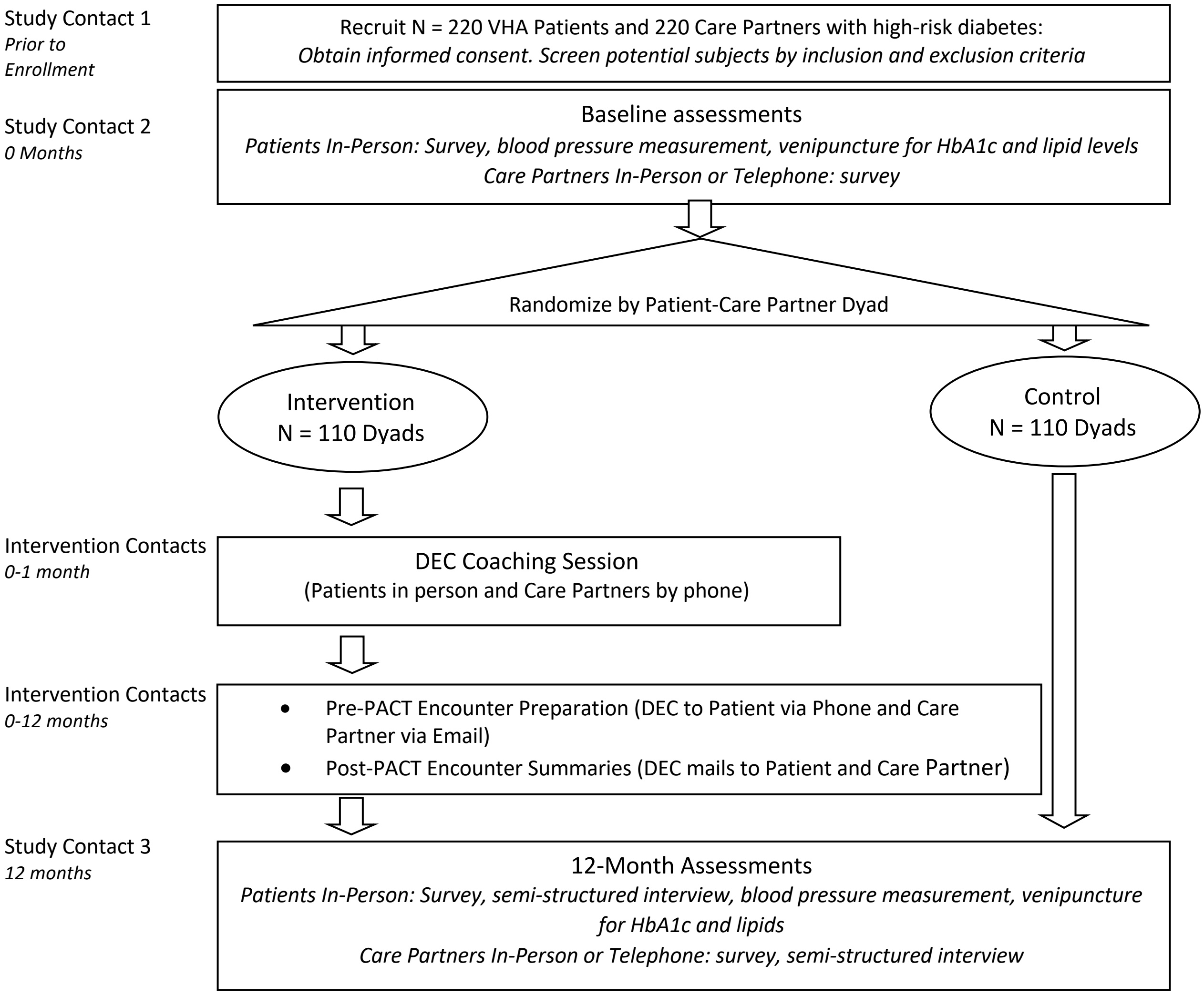

9. Experimental Plan

9.1 Study Inclusion and Exclusion Criteria

9.1.1 Participant Inclusion Criteria

Patient Inclusion criteria:

- Provide signed and dated informed consent form
- Willing to comply with all study procedures and plan to be be available for the duration of the study
- Validated though completion of patient study screener:
  - Male or female, age 25-70 years old
  - Plan to get most diabetes care at [clinic] over the subsequent 12 months
  - Able to use telephone to respond to weekly automated IVR calls
  - Be able to identify an adult family member or friend who is regularly involved in their health management or health care (involved with medications, managing sugars, coming to appointments, etc)
- Validated through patient medical record:
  - Have a diagnosis of diabetes and be at high-risk for diabetes complications, defined as: (1) a diagnosis of diabetes based on encounter diagnoses from 1 inpatient or 2 outpatient encounters (ICD9 code of 250.xx, 357.2x, 362.xx, 366.41, 962.3 or E932.3) OR a diabetes

medication (at least one >3 month prescription from VA drug classes HS501 (insulin) or HS502, other than metformin), (2) have an assigned [REDACTED]HS primary care provider and at least 2 visits to [REDACTED]HS primary care in the previous 12 months, (3) poor glycemic control (last HbA1C >9 or HbA1C >8 among patients <55 years old) OR poor blood pressure control (last BP 160/100 or mean 6 month BP >150/90)
  - Active primary care patients - at least 2 visits to qualifying clinic in last 12 months

Care Partner Inclusion Criteria:

- Validated through completion of Care Partner study screener:
  - Between 21 and 75 years old
  - Fluent in English
  - Have continuous phone service (land line or mobile) or internet access
    - (Note: computer/internet access <u>not</u> required)
  - Live in the United States

<u>9.1.2 Participant Exclusion Criteria</u>

Patient Exclusion Criteria:

- Validated through completion of patient study screener:
  - Expect to have >1 month gap in [REDACTED]HS care in the 12 months following enrollment (e.g. snowbird travel).
  - Plan to receive the majority of their care for diabetes mainly from a non-PACT provider in the 12 months following enrollment
  - Have a VA resident/trainee as their main primary care provider
  - Live in a nursing home OR assisted living
  - Have significant cognitive impairment as measured by an EMR diagnosis of Alzheimer's disease or dementia, or a score of 20 or less (which corresponds to moderate to severe cognitive impairment) on the Telephone Interview for Cognitive Status (TICS)
  - Need help with <u>basic</u> ADLs as measured by the Katz Basic Activities of Daily Living Scale
  - Do not speak English
  - Have a life-limiting severe illness (such as ESRD requiring dialysis, COPD requiring oxygen, cancer undergoing active treatment, receiving palliative/hospice care)
  - Are concurrently enrolled in another research study, at time of enrollment, that could conflict with CO-IMPACT's protocol (e.g. another diabetes management research intervention)
  - Do not have a working phone or are not able to use a telephone to respond to automated IVR calls
  - Currently Pregnant

- Validated through patient medical record:
  - Have a serious mental illness or active substance abuse issue as determined by a diagnosis of a mental illness matching any of the following ICD9 Codes (Schizophrenia: 295.0x, 295.1x, 295.2x, 295.3x, 295.4x, 295.6x,295.7x,295.8x,295.9x, Bipolar: 296.0x, 296.1x, 296.4x, 296.5x, 296.6x, 296.7x, 296.8x, Delusional Disorders: 297.0x,297.1x,297.2x,297.3x,297.8x,297.9x, Other psychoses: 298.0x, 298.1x, 298.2x, 298.3x, 298.4x, 298.8x,298.9x, Substance abuse: 303.xx, 304.xx, 305.xx, 291.xx, 292.xx)

Care Partner Exclusion Criteria:

- Validated through completion of Care Partner study screener:
  - Receive pay for caring for the patient
  - Have significant cognitive impairment as measured by a score of 20 or less (which corresponds to moderate to severe cognitive impairment) on the Telephone Interview for Cognitive Status (TICS)
  - Need help with <u>basic</u> ADLs as measured by the Katz Basic Activities of Daily Living Scale
  - Have a life-limiting severe illness (such as end-stage renal disease requiring dialysis, chronic lung disease requiring oxygen, cancer undergoing active treatment, receiving palliative/hospice care)

9.2 Recruitment
We will recruit 220 pairs of patients and Care Partners, or dyads, receiving care for diabetes, who are also at high-risk for diabetes complications, in the one parent facility and three CBOCs affiliated with the VA [redacted] Healthcare System ([REDACTED]AHS). Dr. Ann-Marie Rosland has direct experience recruiting representative samples of VA patients with diabetes, and patients' Care Partners, to multiple studies, and has successfully engaged Veterans and their Care Partners in a pilot study of the proposed intervention.

9.2.1 Patient Recruitment
[redacted] VA patients between ages 25 and 70, with high-risk diabetes will be identified from the [redacted] VA PACT lab high-risk diabetes patient registries. Identified patients will be sent an introductory letter informing them about the study and inviting them to participate. Veterans will be told they can opt out of further contact by calling study staff via a toll-free number or returning a postage-paid response card. In the absence of such notification, 10 days after mailing the letter, study staff will call Veterans to explain the study in more detail, conduct initial screening, and solicit their involvement. Patients will be contacted a maximum of three times to initiate study contact. Repeat calls will be made once every 3 – 5 days. If patients are unreachable or do not respond to messages left by study staff they will be removed from the contact list.

During the initial contact call, the study will be described and patients will have ample and repeated opportunities to ask questions. Willing and eligible Veterans will be asked to identify a Care Partner to participate in the study with them. Veterans will be offered their choice of either contacting the potential Care Partner first themselves, followed by a study team call to the Care Partner after a 3 day waiting period; or having the study team contact the potential Care Partner by telephone first. For Patient/Care Partner pairs (dyads) meeting eligibility criteria, patients will be asked to come for in-person informed consent and baseline study assessment. Patients will also be invited to sign a HIPAA release to give permission for study staff and VA clinic staff to share personal health information with their Care Partner

9.2.2 Care Partner Recruitment
Care Partners will be identified during the initial screening phone call to the patient. Care Partners will then be contacted by phone to screen for interest and eligibility. Interested and eligible Care Partners will be mailed further information, then consented either in person or by phone.
Care Partner participants will be identified by patient participants by asking, "Do you have a family member or friend who gets involved with your health care in one of these ways..." followed by a list of specific support roles (help with medications, help with home glucose test results, tracking medical information, or coming to doctor's appointments).

9.3 Enrollment
9.3.1 Patient Participants
At in-person enrollment, study staff will describe the content of the study in detail, including that patients and Care Partners can decline participation at any time or decline Care Partner participation in care at any time. Patients will also be told in detail the type of clinical information that Care Partners may have access to should they be randomized to the intervention group. After informed consent is obtained, the RA will perform the pre-intervention baseline assessment, including survey and BP measurement. The RA will also enter the order for labwork, including an HbA1c test and lipid panel to be conducted at the VA clinical laboratory. If patients have had an HbA1C performed at a [REDACTED]HS facility as part of routine care within 4 weeks prior or 2 weeks after their study baseline date or 2 weeks before or 4 weeks after their 12-month assessment date, or if they had a lipid panel performed within 4 weeks before or after a study assessment date, that test result will be used for the study assessment and an additional study-associated venipuncture will not be performed. Based on the average time needed to complete baseline assessments in Dr. Rosland's CO-IMPACT pilot study, we anticipate this assessment will take about 45 minutes to complete, exclusive of time required to complete bloodwork at the VA Laboratory. Patient participants will receive $50 upon enrolling and completing the pre-intervention baseline assessment.

9.3.2 Care Partner Participants
After informed consent is obtained from Care Partners, the RA will conduct a pre-intervention baseline assessment. Based on the average time needed to complete baseline assessments in Dr. Rosland's CO-IMPACT

pilot study, we anticipate this assessment will take about 45 minutes to complete. Care Partner participants will receive $20 upon enrolling and completing the pre-intervention baseline assessment.

9.4 Randomization Procedures
After baseline assessment, dyads will be randomly assigned in equal numbers to the two study conditions. Allocation will be concealed, with the DEC randomizing participants to study arms using sealed opaque envelopes, and a computer-generated randomization series. Randomization will be done within blocks of patient-supporter dyads that live together versus those that live apart.

9.5 Single-Blinding Procedures
Study analysts and the study PIs and CO-Is will be blind to who in the study is assigned to the intervention versus control groups. RAs, who will be responsible for primary outcome assessment through conducting follow up assessments, will be blind at the time of both the baseline and 12 month survey and blood pressure measurement. The RA will then open a sealed envelope revealing the participants study assignment with instructions as to which semi-structured interview guide to use (intervention vs. control participant tailored). The assigned group for each dyad will not be detectable through study databases used for assessment data entry or data collection. All participants will be assigned sequential numbers at the time they are screened so Study IDs will be unrelated to which group participants are assigned to. The DEC will by necessity be aware of which participants are assigned to the intervention group, however DEC databases tracking intervention participation will be kept separate from study assessment databases until data collection has been completed.

9.6 Subject Withdrawal
9.6.1 Reasons for Withdrawal
Subjects may choose to stop participating for any reason at any time.

If a subject becomes newly ineligible during the study, according to eligibility/ineligibility criteria listed in section 5 above (i.e. develops a terminal illness) they will be notified that their eligibility has changed and withdrawn from the study by study staff. With their permission their study data up to that point will be retained for Intention to Treat analysis purposes.

9.6.2 Handling of Subject Withdrawals or Subject Discontinuation of Study Intervention
Subjects may choose to stop participating at any time. We will record the date and reason for withdrawal. We will seek permission to retain study data up to the point of withdrawal from the subject for the purposes of intention to treat analysis. For patient participants who wish to stop participating in the intervention or in primary data collection (surveys, study-related lab measurements) we will ask if they are willing to remain in the study for purposes of secondary data collection only (data available in the subjects' medical record collected per study protocol).

If a subject stops responding to study contacts (DEC calls, assessment calls, IVR contacts) study staff will contact them directly to determine whether they would like to continue with the study. Participants will be contacted no more than twice per month up to a total of 3 contacts. In the absence of any response participants will remain enrolled in the study. Patients who are unable to be reached for their 12 month follow up survey will be labeled as 'lost to follow up' for analysis purposes.

If a Care Partner becomes ineligible, or elects to withdraw from the study, patient-partners assigned to the intervention group will be offered the option to continue with patient-focused IVR calls and pre and post-appointment calls for the remaining duration of the study period. All patient participants who remain enrolled but whose Care Partners drop out will be contacted for the 12 month assessment and be included in intent-to-treat analyses.

9.7 Procedures to Maintain Study Enrollment
A one page study newsletter containing generic diabetes health information will be mailed to all participants, in both the intervention and control groups, every three months. The newsletter will contain contact information for study staff as well as general diabetes information consistent with usual VA PACT care.

10. Intervention

10.1 Preparation

10.1.1 Training of Dyad Engagement Coach

The Dyad Engagement Coach will be trained in all study protocols and extraction of clinical data for initial visit and post-visit summaries will be evaluated before the start of the trial.

10.2 Primary Care Staff Orientation

Primary care staff (primary care providers, nurses, and clerks) will be oriented to the intervention prior to intervention start at regularly scheduled provider & staff meetings. The study process for screening patients and Care Partners will be described in detail, and providers will be asked to notify study staff if they do not wish for their patients to participate in general. We will also share the clinically related forms that the Dyad Engagement Coach will use for pre and post visit contacts. We will also ask primary care staff to report to study staff any Care Partners that they feel are interfering with patient well-being or clinical care. In a separate training session clinical staff will be given a brief training and reference materials on positive and productive communication with patients' Care Partners.

10.3 Initial Visit with the Dyad Engagement Coach (DEC)

This visit will take place within 2 weeks of enrollment. The patient will meet in-person with the DEC at [REDACTED]AHS. The Care Partner will either be present in person or on speakerphone during the visit. Care Partners who participate by phone will be pre-mailed printed materials and guided to the intervention website during the session if possible. This visit is anticipated to take about 45 minutes.

10.3.1 Agenda of the Initial Visit

- Assess the supporter's current role(s) in patients health care
- Review the patient's diabetes complication risk status: last HBA1C, blood pressure, lipid levels, smoking status, and calculated UKPDS 5-year cardiac risk score
- Review the patient's latest diabetes plan based on medical record progress notes and prescriptions
- Ensure that each patient has a glucometer, glucometer supplies, and home blood pressure cuff
- Educate the dyad about members of the patient's PACT teamlet, their roles, and how to reach them
- Educate the dyad about diabetes risk reduction programs available in PACT
- Review guidelines to being an activated patient
- Review guidelines on evidence-based support skills including 1) positive and autonomy supportive communication, 2) action planning steps, 3) effective supporter communication with patients' medical providers(Both guidelines presented via web-based slide show)
- Review structured talking-points for biweekly patient-supporter discussions about diabetes action plans

A written summary of this session will be given to patients and Care Partners and placed in the patients' medical record for their PACT teamlet to view and co-sign. The DEC will also alert the patient's teamlet RN if patient expresses interest in a referral to a VA program.

10.3.2 Initial Visit Materials

After the initial session, patients and Care Partners will be able to review the guidelines and talking points discussed via a study website and a printed workbook. These materials will include general information about diabetes management that is identical to that which patients receive in VA diabetes and PACT education. Information about accessing PACT services, based on standard PACT orientation brochures will be included. Additionally, tips for patient-clinician communication and visit preparation will be based on VA brochures such as "TEAM UP For Your Care".

General Diabetes, VA, and Care Partners Information:

- General information about diabetes management
- General ways Care Partners/family can facilitate diabetes management in day-to-day life
- Steps in effective goal setting

- Care Partners program guidelines and limits of Care Partner roles
- How CG can provide autonomy-supportive encouragement
- Info on PACT teamlet members, their roles, and contact information
- Importance of patient/Care Partner activation between visits and active participation in encounter
- Tips on best Care Partner communication with veteran's doctors/nurses
- Info on obtaining medications and making appointments at the VA
- Info on VA diabetes programs available and on MyHealth*e*Vet (standard brochures available to patients at [REDACTED]VA)

Program Tools

- Worksheets to record glucose and blood pressure testing results
- Worksheets to record medication regimen, medications taken
- Weekly conversation guide
- Worksheets to record events – such as illnesses, ED visits
    - Steps to take if go to a non-VA facility
- Worksheets for primary care appointments
    - Questions for provider by type
    - Information to bring from home
    - Information to bring from outside providers
    - Current goal(s) and progress towards/barriers against them

10.4 IVR Component

Patients will receive automated IVR assessment calls once every two weeks. The goal of these calls will be to prompt continued action planning and Care Partner involvement between PACT visits.

Patient calls will consist of statements and queries recorded in a human voice, to which they can respond using their touch-tone pad. During each call, patients will be asked whether they are experiencing any diabetes management concerns for which taking action within the next week would be prudent. These include more than two fasting home glucose readings over 160 or one under 70, two home blood pressure readings over 150/95 or systolic blood pressures <90, bothersome medication side effects, or running short on medication supply. If any relevant health issues are noted in the responses, the patient will be asked at the end of the call to identify from a menu what action they plan to take. Options include making a plan with their Care Partner, contacting the PACT team by phone, contacting the VA pharmacy, or making a plan on their own. After a patient completes an IVR call, the Care Partner will receive an automated summary, via structured email or IVR call, with any identified action issues and the patient's plan. In this way, the supporter can follow-up to support the plan that the patient has chosen, consistent with the concept of 'need-responsive support', that support is more effective when it meets a need identified by the recipient. Supporter messages will include reminders to discuss diabetes care with the patient, using the talking-points and guidelines provided at the initial session. The patient's PACT nurse care manager will receive an automated fax alert when patients identify clinically urgent issues (such as ≥2 blood sugars <70 or over 400, or SBP >180 or <85).

10.5 Patient-Care Partner Weekly Discussions

Participant guidelines specify several parameters for Care Partner-patient interactions. These are to: (a) talk at least once weekly about DM for at least 5 minutes per occasion, (b) use supportive comments and avoid criticism, (c) review recent assessments and trends, (d) collaboratively approach problem solving and set action plans, (e) review progress and barriers to achieving past action plans, and (f) monitor concrete issues as needed (e.g., medication supplies, upcoming appointments). The patient will be encouraged to contact his or her clinical team in appropriate situations.

10.6 Pre Appointment Preparation

After the initial coaching session the study team will monitor VISTA appointment files for enrolled patients to identify upcoming PACT visits. A qualifying visit will be an in-person visit to a PACT PCP, nurse, or clinical pharmacist. Approximately one week before each qualifying visit the Dyad Engagement Coach will conduct a preparation session with the patient via telephone. During that call, the DEC will use a visit preparation template to help the patient identify any diabetes risk-related questions or concerns they would like to address

during their visit, as well as diabetes-related information, such as home monitoring logs, they will bring to the visit. The DEC will help patients role-play, asking one or two questions most important to them. Patients will be free to add non-diabetes related questions or information to their pre-visit plan, but these will not be specifically elicited by the DEC. If the enrolled Care Partner is present with the patient at the time of the call they will be invited to participate in the call. The DEC will email a website link for the visit preparation template to the patient's Care Partner, asking them to complete it with their questions and concerns for the patient's visit. Supporters who do not have access to email will be offered a telephone preparation call similar to the patient call. The coach will prompt both patients and supporters to share their questions/concerns with one another before the patient's visit.

The DEC will document this call in CPRS and add the teamlet LPN as co-signer. Thus, the teamlet LPN will not need to conduct their usual pre-visit reminder phone call for this patient.

10.7 Post Appointment
Within 1 week of a completed, qualifying PACT visit the Dyad Enagement Coach will mail, or post to the study secure website, an after-visit summary, based on the Ambulatory Daily Plan, to both the patient and their Care Partner. The Ambulatory Daily Plan is a template programmed into the VA electronic medical record (EMR) that, with one click, generates and prints a patient-friendly summary of visit care. The Ambulatory Daily Plan is available in the PACT toolkit, and is currently used in at least 6 VA facilities for over 15,000 outpatient encounters/month.

10.8 Intermittent Test Results and Changes in Medications
On medical record scans, the Dyad Engagement Coach will note if the patient's primary care team communicated diabetes-relevant test results or regimen changes to the patient. The DEC will then contact the Care Partner (mail or web site post) this information 5 days after the patient contact, if not already done by the care team. An addendum will be placed on the relevant chart note when this is done.

10.9 Fidelity
A predetermined sequence (the first 10, then 10% of the remaining by random number generation) of DEC initial and telephone sessions will be recorded for review by the study PI, along with coach-created documents. Patient appointment and IVR call records will be monitored monthly by study staff for level of missed contact opportunities.

Audiotaping of DEC sessions is not required for the intervention, and participants who decline to be taped will still be able to fully participate in sessions. These tapes will be not be labeled with participant IDs and will be destroyed immediately after review by the PI.

11. Control Condition

Patients assigned to the control condition will receive usual PACT care for diabetes at a facility that is at an advanced stage of PACT implementation. PACT care for diabetes is expected to follow VA/DoD diabetes management guidelines. These patients are then eligible for PACT services at the teamlet's discretion.

Study staff will also provide control group patients with a packet of general diabetes management information, and ensure they have home glucometers and blood pressure cuffs. Patients in the control condition will not be precluded from involving Care Partners in medical visits or VA health programs.

12. Data Collection Procedures

12.1 Patient
Baseline:
An assessment consisting of self-reported survey items and blood pressure measurement will be conducted at the time of in-person informed consent by study staff. HbA1C tests and Lipid Panels will be obtained at this time through the VA lab.

12 Months:

An assessment consisting of self-reported survey items, a short semi-structured interview on the feasibility and usefulness of each intervention component will be conducted in-person by study staff at 12 months after the baseline assessment. HbA1C tests and Lipid Panels will be obtained separately through the VA lab. If patients have had an HbA1C performed at a [REDACTED]HS facility as part of routine care 2 weeks before or 4 weeks after their 12-month assessment date, or if they had a lipid panel performed within 4 weeks before or after an assessment date, that test result will be used for the study assessment and an additional study-associated venipuncture will not be performed.

Patient pharmacy and encounter EMR data will be collected from periods 12 months prior to baseline and the 12 month intervention period.

12.2 Care Partner

Baseline:

A baseline assessment consisting of self-reported survey items will be conducted over the phone by study staff after CP consent.

12 months:

A 12 month assessment consisting of self-reported survey items and a short semi-structured interview on the feasibility and usefulness of each intervention component will be conducted over the phone by study staff at 12 months after the baseline assessment.

12.3 Dyads

Sixteen Dyads will be purposively sampled for semi-structured qualitative interviews at 12-15 months after baseline (see section 13.6 below)

12.4 PACT Staff

After all 220 dyads have completed the 12 month study period,14 PACT Staff ((4 PCPs, 4 RN care managers, 4 clinical associates and 2 clinical pharmacists) from teamlets with the most patient participants will be invited to participate in a semi-structured interview about their experiences with the intervention. Interviews will be scheduled during non-work hours (including lunch breaks), and staff participants will not receive an incentive.

12.5 Intervention Processes

The CO-IMPACT automated IVR system will capture patient participants' responses to questions about symptoms, medication adherence, and home glucose and blood pressure readings. The system will automatically track dates and times of all assessment attempts and whether they are completed. The CO-IMPACT website will automatically collect data on access to various parts of the website. Data from coach session logs will be also be captured.

12.6 DEC Experiences and Feedback

After all 220 patients have completed the study period, study staff will conduct a semi-structured interview with the DEC

13 Study Measures

13.1 Patient Outcomes

Health Behaviors and Behavioral Determinants: The study's main outcome measure will be the Patient Activation Measure-13 (PAM-13). The PAM-13 has been widely used to measure patient activation in longitudinal studies, and in clinical trials as a primary outcome measure, and scores have been responsive to intervention. The PAM-13 is reliable (Cronbach alpha 0.87), and improvement in PAM-13 scores has been linked to improvement in self-management behavior. A 4-6 point change in the PAM is considered clinically significant. We will also measure patient activation in medical visits with the Perceived Efficacy in Patient-Physician Interactions (PEPPI-5). Items include "I am confident in my ability…to get a doctor to answer all of my questions" and "to get a doctor to take my chief health concern seriously". The PEPPI-5 has been validated against other self-efficacy and patient satisfaction scales, and is reliable (Cronbach alpha 0.92).

Health Risks: To address the effect of CO-IMPACT on patient health risks, our main measure will be the 5-year UKPDS Risk Engine. This score estimates the risk of a coronary heart disease (CHD) event (fatal or non-fatal

MI, or sudden death) specifically among people with diabetes. The score components include factors we hypothesize could be improved by the intervention, including HbA1C, systolic blood pressure (SBP), total cholesterol/HDL cholesterol ratio, and smoking status. The score also includes age, sex, race/ethnicity, and length of time since diabetes diagnosis. The UKPDS Risk Engine has been validated in multiple populations. A 1-2% change in risk is considered clinically significant at a population level. In preparation for this study, we measured UKPDS risk among 434 [REDACTED]HS patients randomly selected from the high-risk diabetes registry (mean 5-year risk 18%, SD 12%), and simulated the changes in risk that would result from changes in individual score components. We found that an average 0.5% decrease in HbA1C over 1 year led to an average 1.3% decrease in UKPDS risk over that of the same population with no change in HbA1C. Similarly, a 10mmHg decrease in SBP led to a 1.3% risk decrease, and a 30% decrease in total cholesterol among those with total cholesterol >160mg/dL (to simulate new adherence to a statin) led to a 3% risk decrease. The combination of these changes in HbA1C, SBP, and cholesterol led to a 5% average risk decrease (IQR 2-7%).

HbA1C, lipid levels, blood pressure, and smoking status will be analyzed independently as secondary health outcomes. We will measure via survey patients' frequency of hypoglycemia, and diabetes distress. Patients' use of VA urgent care will be extracted from the EMR for the period 12 months prior to intervention start and during the 12 month study period, supplemented by patient report of non-VA urgent care.

Table 2: Details on Selected Patient Measures

Construct	Source	Instrument(s)	BL	12M
**Health Behaviors and Determinants**				
Activation	Survey	PAM-13	X	X
Activation in Health Encounters	Survey	PEPPI-5	X	X
Diabetes Self-Efficacy	Survey	Stanford Chronic Disease Self-Efficacy Scale [56]	X	X
Diabetes Self-Management Behavior (self-monitoring, healthy eating, physical activity)	Survey	Summary of Diabetes Self-Care Activities[57]	X	X
Diabetes Medication Adherence	EMR x12 months	Cumulative Medication Gaps <20%[58]	X	X
**Health Outcomes**				
5-Year Cardiac Event Risk		UKPDS 5 year cardiac risk score	X	X
Glycemic Control	Venous Sample	HbA1C	X	X
Blood Pressure	Direct measure	Systolic Blood Pressure, Mean Arterial Pressure	X	X
Lipid Levels	Venous Sample	Total Cholesterol/HDL	X	X
Smoking Status	Survey		X	X
Diabetes Distress	Survey	Problem Areas in Diabetes Scale[59]	X	X
**Patient-Supporter Relationship and Support Quality**				
Patient-Supporter Relationship Quality	Survey	Relationship Rating Form – Respect Subscale[60]	X	X
Patient Satisfaction with Diabetes Social Support	Survey	Diabtes Care Profile – Support Subscale[61]	X	X
Supporter use of Autonomy Supportive Communication	Survey	Important Other Climate Questionnaire[62]	X	X
**Patient-Provider Relationship**				
Patient-provider trust	Survey	Primary Care Assessment Survey-Trust Subscale[63]	X	X
Patient-provider shared decision making	Survey	Provider Participatory Decision-Making Style[64]	X	X

**Potential Moderators**				
Time with Diabetes	Survey		X	
Patient Comorbidities	EMR x12 months	Charlson Comorbidity Index[65]	X	
Health Literacy	Survey	Brief Health Literacy Screen[66]	X	
Depressive Symptoms	Survey	PHQ-8[67]	X	

Patient-Supporter Relationship and Support Quality: We will measure overall relationship quality for both patients and supporters (see Tables 2 and 3). Patient satisfaction with overall quality of diabetes support received and supporter use of autonomy-supportive communication will be assessed via patient survey. Supporters and patients will be surveyed about concerns about health privacy breaches.

Patient-Provider Relationship and *Patient Satisfaction with VA Health Care:* We will measure patient-provider communication, trust, and level of shared decision-making via patient survey (Table 2). We will measure patient satisfaction with PACT care using a question from the VA Consumer Assessment of Healthcare Providers and Systems (CAHPS)-PCMH, and patient satisfaction with PACT engagement of Care Partners using questions developed in our pilot.

13.2 Care Partner Outcomes

We will measures changes in Care Partner roles (e.g. help track patient medication use at home) via surveys at baseline and 12 months. Care Partners' self-efficacy for helping patients with diabetes, supporter distress about the patient's diabetes, and supporter distress about patient hypoglycemia, will be measured with adaptations from similar validated patient measures. These supporter-adapted measures were used in our pilot intervention assessment. In this study we will calculate psychometric properties of these measures, and associations with validated supporter measures, among our 220 Care Partners. Caregiving burden will be assessed with the reliable and validated Multidimensional Caregiver Strain Index.

**Table 3: Details on Selected Supporter Measures**				
Construct	Source	Instrument(s)	BL	12M
**Behaviors and Determinants**				
Supporter Self-Efficacy for Helping Patient With DM Care	Survey	Adapted Stanford Chronic Disease Self-Efficacy Scale[56]	X	X
**Health and Relationship Outcomes**				
Caregiver Burden	Survey	Caregiver Strain Index[83]	X	X
Supporter Distress About Patient's Diabetes	Survey	Adapted Problem Areas in Diabetes Scale[59]<br>Adapted Fear of Hypoglycemia – Worry Subscale[84]	X<br>X	X<br>X
Patient-Supporter Relationship Quality	Survey	Relationship Rating Form – Respect Subscale[60]	X	X
**Potential Moderators**				
Depressive Symptoms	Survey	PHQ-8[67]	X	

13.3 Patient and Supporter Moderators of Effect

Theoretical patient moderators of intervention effects include (also see Table 2): sociodemographics (sex, age, education), baseline diabetes medication regimen, distance from VA site, comorbidities, health literacy level, and co-morbid depressive symptoms. Additional moderators include: whether the patient and supporter live together, whether the supporter has diabetes, supporter depressive symptoms, baseline patient-supporter and patient-physician relationship quality, and whether Care Partners attend patient visits in person.

13.4 Provider Behavior and Impact on PACT Teamlet

A theoretical mediator of intervention effect, medication intensification, will be measured via EMR similarly to methods used by Dr. Kerr previously.[41, 42] In interviews with PACT clinical staff we will assess whether clinicians perceived changes in effectiveness or efficiency of patient-clinician communication, any unintended consequences on privacy or clinician comfort, clinician awareness of supporter roles at home and relationships with patient supporters.

13.5 Intervention and Control Processes

We will record the frequency of each type of DEC contact with intervention-assigned participants, and time spent in preparation and execution of each contact. We will automatically capture outcomes of all IVR call attempts, and number of downloads from the study website. For participants in both arms we will capture via the EMR the number of completed PACT PCP, nurse, and clinical pharmacist encounters, occurring in-person or by phone. We will ask participants via survey whether they received after-visit summaries after PACT in-person visits. We will tally consults entered by PACT teamlets to diabetes risk related programs, and patient (via EMR) and supporter (via survey) rate of attendance. Finally, we will ask all patients and supporters about the frequency of general discussions about diabetes, pre-visit preparation discussions, and post-visit debriefing.

13.6 Facilitators and Barriers to Future Implementation

We will ask eligible patients and Care Partners who decline participation to provide consent for a brief survey including reasons for not enrolling. We will conduct semi-structured interviews of selected participants and clinicians to evaluate facilitators and barriers to intervention implementation. Eight dyads will be purposely sampled from those with high vs. low engagement in the intervention (as measured by rate of pre-visit and IVR call completions) and eight from those with high vs. low level of improvement in cardiac risk score. We will also interview PACT staff (4 PCPs, 4 RN care managers, 4 clinical associates and 2 clinical pharmacists) from teamlets with the most patient participants.

14. Statistical Analysis Plan (Initial)

14.1 Overall Approach

We will follow international guidelines for analysis and reporting of clinical trials. We will examine baseline data for prognostically important differences across the two study groups, such as patients' age, race, comorbidities, and baseline use of services. Although we do not anticipate any imbalances, any baseline differences between experimental arms will be included as covariates in analyses comparing outcomes. Missing data will be imputed for non-outcome measures, using multiple imputation methods. If we find baseline variables to be associated with the loss to follow-up, we will include those baseline variables as covariates in models evaluating the intervention effect.

14.2 Unit of Analysis and Sample Size Calculation

Our main aims (1 and 2) are to evaluate effects at the patient level. Our sample size calculations are based on our primary outcome of patient activation, measured by the PAM-13. We calculated our sample size to provide a minimum of 80% power to detect between group differences in PAM-13 change of 5.0, with a standard deviation of change of 12.5, and a two-tailed alpha of 0.05. To achieve 80% power, a minimum of 99 patients is needed in each group, for a total sample size of 198. To allow for 10% attrition, we will enroll 110 patients in each group, for a total of 220 patients. This sample size will also be sufficient for detecting differences in the secondary outcome of change in UKPDS risk score. Our sample size of 99 per group will provide 80% power for detecting between group differences in predicted risk of 2.0% (SD=5.0). Based on our estimates a 2.0% or greater change in intervention patients is achievable and would be clinically significant.

14.3 Primary and Secondary Outcomes (Aims 1 and 2)

We will first evaluate bivariate associations between the study group condition (by intention to treat principles) and outcomes using two-sided, two-sample t–tests for continuous measures and Pearson's chi-square tests for categorical measures. We expect that key outcomes such as the PAM-13 will be normally distributed, but measures will be transformed if needed. We will then use multivariable regression models, taking into account baseline score of the outcome, to identify main effects.

14.4 Mediators and Moderators of Intervention Effect (Aim 3)

We will use multivariable regression models to examine potential mediators and moderators of intervention effects. We will introduce potential mediators to models linking intervention condition to outcomes, examining changes in the magnitude of the relationship between the intervention and the outcomes before and after the covariates are introduced. We will also use the Preacher and Hayes bootstrapping method to examine potential mediators to determine whether the mediation effect is significant. This is a non-parametric method that can be used when the outcome violates assumptions of normality. Potential mediators are specified in our theoretical model (Figure 1), and include an index of Care Partner engagement in the intervention, composed of measures of supporter participation in intervention sessions, and reported use of pre-visit preparation and debriefing tools. Analyses of potential moderators (as in section 13.3) will use standard approaches to evaluate interactions between these covariates and the intervention, which will include plotting regression lines for high and low values of the moderator variable using Stata routines.[90] Independent variables and moderators will be centered before testing interactions, so that multicollinearity between first order and higher-order terms will be minimized.

14.5 Qualitative Analysis

We will conduct a thematic analysis of interview transcripts using the "Editing Analysis Style,"[91] which contains both deductive and inductive elements. Following this approach, Drs. Rosland and Heisler will independently read interview transcripts, break down responses into individual segments that express a single idea or theme (e.g., ways participants found pre-visit calls useful or not useful) and label these phrases with appropriate codes. An iterative process will be used to compare results until agreement is reached on the codes and their definitions, after which we will apply the codes to the remaining transcripts. Emerging themes will be compared across patients and compared to patterns in survey responses.

14.6 Process Evaluation

We will use the RE-AIM framework[92] to guide this analysis. To analyze the potential reach of the intervention we will calculate the proportion of patients with diabetes who meet inclusion criteria and compare characteristics of eligible and non-eligible dyads. Effectiveness will be measured via our main outcomes and differences in outcomes among key patient groups. We will evaluate adoption by examining the characteristics of patients and supporters who decline enrollment and their reasons for declining. We will also examine retention/dropout from the study and reasons, length/frequency of DEC sessions, % of potential DEC sessions completed, and IVR call adherence (% attempted calls completed, # weeks adherent to calls). We will analyze facilitators and barriers to implementation among dyad and staff interview themes using the Consolidated Framework for Implementation Research (CFIR). The CFIR's five major domains are the intervention, inner and outer setting, the individuals involved and the implementation process.

15. Study Oversight, Quality Control

Study investigators and staff will be responsible for study oversight and maintaining the highest standards of intervention delivery throughout the study period. The principal investigator, Dr. Rosland, will maintain appropriate oversight of this research protocol and study staff, including recruitment, selection of study participants, study conduct, and delegation of research responsibilities. Bi-weekly meetings will be conducted throughout the study period to review all study activities. All study investigators will review study materials and protocols prior to the start of the intervention in order to provide input on best practices for managing patient safety and privacy. Additionally, the intervention will undergo annual and continuing review through the [redacted] IRB and comply with all yearly consent form audits as well as 3 year full regulatory audits. To remain prepared for regulatory audits the project will maintain a regulatory binder which meets all regulatory requirements and is kept up to date throughout the study period.

16. Timeline

	Year 1				Year 2				Year 3				Year 4			
	Q1	Q2	Q3	Q4	Q1	Q2	Q3	Q4	Q1	Q2	Q3	Q4	Q1	Q2	Q3	Q4
IRB Review																
Hire/Train Research Assistants																

Hire/Train Dyad Engagement Coach																
Refine and Finalize Study and Intervention Materials																
Participant Recruitment																
Participant Enrollment/Assessment																
12 Month Participant Assessment																
Qualitative Data Collection																
Data Analysis																
Write Reports/Manuscripts																
Dissemination Planning																

17. Ethics/Protection of Human Subjects

17.1 Risks to Subjects

17.1.1Patient and Care Partner Participants

Physical risks: Patient participants will undergo venipuncture for HbA1C and lipid panels twice over 12 months. If patients have had an HbA1C performed at a [REDACTED]HS facility as part of routine care within 4 weeks prior or 2 weeks after their baseline study assessment date or 2 weeks before or 4 week after their 12-month study assessment date, or if they had a lipid panel performed within 4 weeks before or after an assessment date, that test result will be used for the study assessment and an additional study-associated venipuncture will not be performed. However we estimate that at least 80% of patient participants will undergo a venipuncture that would not be required by routine clinical care. Risks of this minor procedure include brief pain, bruising, and minor bleeding. This study does not involve pharmacotherapy. Care Partner participants will not undergo venipuncture or blood pressure measurement.

Primary care physicians who do not opt out of receiving study updates for their patients will be notified of the results of all labwork conducted during the study. Usual care will be followed in accordance with the results of labwork. For patient's whose primary care phsyicians opt out of study related updates the PI, Dr. Rosland, will be listed as the provider for any labwork related to the study and she will follow up as necessary regarding any abnormal results. If the patient's blood pressure is <90/50 or >180/110 the RA will escort the patient to the (on-site) VA urgent care clinic. If the patient's blood pressure is >140/90 but <180/110 the RA will recommend that the patient call his or her primary care doctor and will also contact the patient's team RN the same day.

Psychological risks: Additionally, it is possible that some participants may find that being interviewed or audio-taped is stressful. Almost all of the survey questions (and all of the questions that are related to sensitive issues such as depressive symptoms) have been used in multiple prior studies conducted by our team, and our participants have not reported that the questions increase their burden or anxiety. Patients and Care Partners will be informed as part of their informed consent process and immediately prior to each interview that they can drop out of the study or have an audio recording stopped at any time and that they can refuse to answer any of the questions.

Patient-participants will receive biweekly automated tele-monitoring concerning their health. Although it is possible that automated calls would become burdensome or annoying, our preliminary studies suggest otherwise. This probability assessment is based upon the high rates of retention and call completion, very high user satisfaction, and the fact that most patients desire that the automated calls become part of their standard care.

It is possible that participation in the CO-IMPACT intervention may cause participant discomfort, strain patient-supporter relationships, strain patient-clinician relationships, or increase Care Partner burden. However the intent of the intervention is to better support patients and family members, decrease their diabetes and caregiving related stress, and improve the quality of diabetes-related communication between the patient and the supporter, and the patient and their clinicians. There was no indication in the CO-IMPACT pilot study, or in

multiple previous studies of patient-supporter IVR interventions, that burden was increased or relationships were strained.

There is a small risk that patients will regret sharing certain medical or personal information with their Care Partner. However we are stipulating that the supporter should be someone who is already regularly involved in the patient's health care. In our pilot studies with [REDACTED]HS patients with high-risk diabetes there was minimal concern about sharing health information with a close family member or friend who was already involved in the patient's health care.

Screened patients who are deemed ineligible due to depression will be referred to their primary care doctor. Project staff will also be trained to follow standard protocols if they detect a Veteran is a high suicide risk, including warm handoff to the VA Suicide Prevention Hotline.

Social and Legal: These risks include potential breach of confidentiality, inadvertent release of sensitive information, and the risk of participation due to potential coercion. Rigorous data security measures will be put in place to minimize the risk of breach of confidentiality and both stringent recruitment procedures and staff training will be employed to minimize risk of potential coercion. Those procedures are described in detail in subsequent sections.

<u>*17.1.2 PACT Staff*</u>
Physical risks: none
Psychological: Some staff could find that conducting interviews or being audio recorded is stressful or burdensome. Participation in these activities will be entirely voluntary in order to minimize these potential risks.
Social and Legal: The main risk is the potential breach of confidentiality from the interview data. This risk is extremely small and will be minimized using the confidentiality protections detailed below. A list of contacted and interviewed vs. declined PACT staff will be kept separately for tracking purposes and will not contain interview IDs.

17.2 Adequacy of protections against risks
Recruitment and Informed Consent
We will request a HIPAA informed consent waiver to perform the searches identifying potential participants based on: "38 CFR 16.116 (d) (2): This research presents no more than minimal risk of harm to the subjects, the waiver will not adversely affect the rights and welfare of the subjects, and this research could not be practicably carried out without a waiver." This search protocol involves no more than minimal risk and use of secondary data will not adversely affect patients' rights. The research could not be carried out without the waiver because without access to the electronic medical record data we will have no way of pre-identifying Veterans with high-risk diabetes that might benefit from participating in the trial. We have obtained waivers successfully for similar screening and recruitment approaches in several previous and ongoing studies. We will seek informed consent from any potential participants prior to baseline assessment and randomization.

Identified patients will be sent an introductory letter informing them about the study and inviting them to participate. Veterans will be told they can opt out of further contact by calling study staff via a toll-free number or returning a postage-paid response card. In the absence of such notification, 10 days after mailing the letter, study staff will call Veterans to explain the study in more detail, conduct initial screening, and solicit their involvement. During this call, the study will be described and patients will have ample and repeated opportunities to ask questions. If agreeable, Veterans will be screened by phone regarding key eligibility criteria. Patients who continue to express interest in the study after screening, will be invited to an in-person informed consent and enrollment visit at the [REDACTED]VA. At that time, patients will be invited to sign a HIPAA release to give permission for VA staff to share personal health information with their Care Partner.

Willing and eligible Veterans will be asked to identify a Care Partner to participate in the study with them. Veterans will be offered their choice of either contacting the potential Care Partner first themselves, followed by a study team call to the Care Partner after a 3 day waiting period; or having the study team contact the potential Care Partner by telephone first. Study staff will screen the Care Partner by phone for interest and eligibility. Interested and eligible Care Partners will be mailed further information, then consented either in-

person or by phone. We received IRB approval to verbally consent Care Partners in the CO-IMPACT pilot and other previous studies involving Veterans' Care Partners based on the following: 1) in the control arm, Care Partners will undergo only limited assessment, 2) in the intervention arm Care Partner involvement will be limited to receiving information about patients diabetes status and care plans, and guidelines to discussing diabetes care with patients. We have used this same process in prior studies and have found that it is an efficient and effective way to recruit large samples of Veterans and their Care Partners.
Because our outcome evaluation is guided by the RE-AIM framework, it is important for us to assess the intervention's "reach" among potentially eligible Veterans or Care Partners. Thus, Veterans and Care Partners who decline to participate will be asked to volunteer to answer 3-4 questions over the phone about their sociodemographics (age, race/ethnicity, distance from the VA) and reasons for not participating.

Study staff will provide in-person informed consent to clinical staff participating in study assessment interviews before the start of the interview. These staff will also be asked to sign a separate consent to audiotape form.

17.3 Protection against Risk

Venipuncture Risks: To minimize risk, this procedure will be performed by [REDACTED]HS trained laboratory phlebotomists in [REDACTED]HS facilities with access to on-site physician care if needed. Patient participants who have recently (see timeframe above) had the relevant laboratory test done as part of routine care will not be asked to repeat it for the study assessment.

Survey and Interview Assessment Burden:
Patients and Care Partners will be informed as part of their informed consent process and immediately prior to each interview that they can drop out of the study at any time and that they can refuse to answer any of the individual questions in the assessments.

Interviews with clinical staff will be conducted on a volunteer basis during non-duty hours (i.e. before/after shifts, on breaks), will be limited in length to 30 minutes or less, and staff will not be given any compensation or incentives to participate.

Patient-Care Partner Relationship:
We will reduce the risk of patient-Care Partner conflicts through several strategies. These include the following:

- We specifically structure the Care Partner's role as assistive to the patient. This is conveyed repeatedly in study contacts. For example, Care Partners are instructed to discuss any concerns with patients in a non-judgmental manner, and to offer choices. They are instructed to encourage the patient to be the main contact for the patient's health care providers whenever possible. Our pilot study experiences suggest that under these arrangements, patients welcome the supporter's instrumental and emotional support.
- Clear and redundant presentation through printed participant guidelines for patients and Care Partners to structure their roles encourage effective communication. This information is repeated in smaller chunks throughout the study timeline, both per schedule as in response to study events.

Patient willingness to share their health information with their Care Partner will be explicitly confirmed in their written consent. We will thoroughly explain to patients the type of information that will be included in Dyad Engagement Coach contacts with Care Partners. To minimize risk of patient regret over sharing health information, we are stipulating that the Care Partner should be someone whom is already regularly involved in the patient's health care. No information will be shared with a Care Partner before it is shared directly with the patient. In addition, only information that is directly related to diabetes management and management of risk of diabetes complications will be included in written or oral communications with Care Partners. Thus, information shared with Care Partners will exclude information on potentially sensitive topics, such as psychiatric care or sexual health. The Dyad Engagement Coach will be thoroughly trained by the PI in protocols for extracting diabetes-relevant data from the medical record, and extraction will be tested prior to intervention start, and monitored by the PI throughout the intervention through a random sample of DEC documents (sampled more frequently at the beginning of the study). Patients and Care Partners will be reminded at every study contact that they can decline participation at any time and that the patient can terminate Care Partner participation in care at any time.

Care Partner Caregiving Burden:
A standard measure for Care Partner burden will be administered to each Care Partner participant at study baseline and endpoint. Prior studies involving chronically ill Veterans and family supporters show that Care Partner burden does not increase, and often decreases, as the result of intervention. We will include reminders in Care Partner contacts that they can call the study toll free number if they are experiencing increased caregiving related stress or burden. We will include written materials on reducing Care Partner stress in the packets for every Care Partner participant.

Patient-Clinician Relationship and Clinical Care:
One of the goals of our intervention is to relieve busy clinicians from some of the day-to-day problem-solving that some patients request and require. Nevertheless, it is critical that patients and Care Partners understand that the intervention is not intended to be a substitute for professional-level formal caregiving, and that they should not attempt to address every problem identified via the assessments without input from patients' clinical team. We will take several measures to ensure timely and appropriate use of formal health services when indicated. First, individual patient assessment calls will include explicit reminders about the importance of contacting their clinician if their health deteriorates. We included an option for patients to use their touchtone pad to access their VA clinician's clinic call center at any time during each call. E-mail messages to Care Partners will emphasize the importance of the patient's health care relationship with their VA clinicians, and supporters will be instructed to encourage the patient to contact clinicians directly rather than having the supporter serve as a communication intermediary. However, supporters will also receive the clinicians' name and phone numbers. The intervention packet will also emphasize that chronic illness care is most effective when patients take an active role in their care, and we will provide concrete guidance regarding effective patient - provider communication.

The study will be described to all provider and clinical staff at staff meetings. Providers will be allowed to opt out of the study, thus making patients assigned to their primary care panel ineligible for the study.

Although risks of worsening symptoms will be minimal, patients included in the pilot intervention trial will receive additional clinical monitoring than would be provided as part of routine clinical care. Therefore, any potential adverse effects of the intervention will likely be detectable through the increased monitoring and contact provided by the intervention. Processes will be in place to train all study staff on how to address patients found to be experiencing worsening diabetes outcomes or psychological distress during study-related contacts. Patients will continue to have access to all usually available health care services.

If patients or Care Partners discuss non-urgent health concerns with the Dyad Engagement Coach, the coach will encourage them to discuss their concerns with each other, and record the concerns for discussion with their provider at their next appointment. If patients or supporters discuss urgent health concerns, the health educator will recommend that they call their health care provider immediately, and, for VA patients, offer to connect them directly by phone to the appropriate primary care team. Health educators will be trained to call 911 for any emergency situations they encounter.

During IVR phone assessments, an automated fax alert to the patient's PACT nurse care manager will be generated if clinically urgent issues are identified (such as >=2 blood sugars <70 or over 300, or >=2 SBP >180 or <90). IVR assessments will not ask about symptoms that constitute medical emergencies (i.e. chest pain, loss of consciousness) but patients will be reminded with each call and in study printed materials to hang up and call 911 if they experience a medical emergency.

Overall Confidentiality:
A number of steps will be taken to ensure participant confidentiality. We will obtain written informed consent from each study participant. As part of that consent, participants will be adequately informed about the small risk of a breach of confidentiality and they will be given the option of opting out of participation. After consenting, patients will be assigned a unique study identification number. Paper copies of all consent forms will be kept in a locked filing cabinet. We will create a secure electronic tracking file that maps the subject's identifying information to the arbitrary study identifier, which will serve as the participant's primary identifier for all analytic files. A hard copy of the cross-walk file will be maintained in a locked file cabinet separately from other study-related documents, and an electronic version of the cross-walk will be maintained in a password-protected

directory separately from other study data and accessible only to the study coordinator. The identifiers and linkable information of all individuals who do not become enrolled will be destroyed. We plan to retain only the screening data for these individuals using a new and unlinkable identifier so that we can characterize non-participants for scientific reasons. We will similarly destroy the data of individuals who express interest but decide not to consent, or are lost to follow-up prior to providing written informed consent. Essentially, no screening data will be linkable by anyone to other research or clinical data unless the patient provides written informed consent, and no additional data will be collected on patients who provide informed consent but are later determined to be ineligible to participate, with the exception of a coded identifier on a "do not contact" list maintained only for the duration of the recruitment phase in order to prevent such individuals from being re-solicited.

All surveys and interviews will be conducted in-person or by phone from a private office with a closed door at a [REDACTED]HS site. To minimize the risk of loss of confidentiality, there will be no personally identifying data on the surveys. Participants will complete the survey in person or by phone with a trained research assistant who will enter their data into a web survey hosted by Qualtrics. Qualtrics is a survey software company that provides secure web-hosted surveys. Qualtrics survey website is password protected and data are temporarily stored securely on servers maintained at Qualtrics. This data will then be saved to a secure, password protected folder behind the VA firewall. Individual accounts are password protected, and respondents' data will only be identified through study ID code. Qualtrics has SAS 70 Certification and meets the rigorous privacy standards imposed on health care records by the Health Insurance Portability and Accountability Act (HIPAA). All Qualtrics accounts are hidden behind passwords and all data is protected with real-time data replication. The Qualtrics system has been used successfully in numerous IRB-regulated and approved studies both at [redacted] VA and the University of Michigan.

In addition, audiotaping of selected coaching sessions is necessary to ensure the fidelity of the coaching sessions. Selected participants will be asked to sign a separate consent to audiotape form. Participants who decline to be taped during coaching sessions will complete the scheduled coaching session without audiotaping. The coach will conduct session audiotaping and study staff will conduct semi-structured assessment interview audiotaping. Each audiofile will be labeled only with the participant's study ID#. Recording devices will be kept physically secure in a locked drawer or locked transport case at all times. Interview transcripts will be stripped of all identifying information prior to destruction of audiotapes.

It will be made clear to participants that no information gathered through study baseline and 12-month assessments will be shared with the other member of the dyad (i.e. patient assessment information will not be shared with the patient's Care Partner and vice versa). Lastly, none of the information provided to research assistants will be shared with participants' clinicians unless the patient appears to be in danger (in cases of suicidality, for example) and Dr. Rosland deems it necessary to contact the participant's physician. Electronic assessment data and analytic files will be maintained on servers that are behind secure VA firewalls and protected in accordance with VA data security requirements. Only approved research personnel will have access to study files. Research data will be presented in aggregate statistics only.

Throughout the study, IRB and HIPAA guidelines will be followed to ensure the privacy and integrity of the information we collect. All study staff will have signed a pledge of confidentiality and are trained annually in secure handling of VA research data according to HIPAA and human subjects guidelines. Any breach of confidentiality will be immediately reported to the PI and to the VA [redacted] Healthcare System Human Subjects Committees (and as required to any other IRBs). In addition, any complaints or concerns expressed to the study staff by participants, providers, or anyone else affected by this study will be immediately reported to the PI and the IRB.

Confidentiality of IVR System and Website:
Multiple procedures will be used to ensure the security of data provided by participants during biweekly telephonic assessments. The computer that performs the automated calls cannot accept incoming calls, thereby limiting the risk of database intrusion or hacking. Immediately upon completion of each assessment call, the collected data will be transferred to a secure data center and deleted from the calling computer. The calling computer has separate hardware and software firewalls, and does not permit e-mail or any other form of incoming internet-based connection. All outbound data communications will be wrapped in a secure shell

(SSH) to prevent eavesdropping. The calling computer will not be connected to any internal network, which limits the consequences of a security breach. "Very strong" passwords will be used (requiring upper case/lower case letters and symbols) to prevent hacking. Assessment reports sent to Care Partners by e-mail will only be sent to Care Partners' password-protected e-mail account (to be confirmed by the supporter at enrollment). E-mail reports will not include patient last names, telephone numbers, or any other identifiers. For urgent health problems, a fax will be automatically generated and sent to the patient's clinician. We will verify and maintain our own list of PACT teamlet fax numbers, maintain logs to track fax deliveries, and include a confidentiality statement on the cover page of all fax communications that instructs unintended recipients to notify study staff.

IVR automated calls and website will be hosted from a server at the University of Michigan Computing Center. We have used the proposed hosting and off-site data storage plan in multiple prior and on-going VA studies with VA patients. The plan has been repeatedly and carefully evaluated by the [redacted] VA IRB and ISO, and it is in full compliance with all VHA regulations regarding data security and the protection of PHI. We are continuing to work with our VISN and VA leadership nationally to find a way to host these projects from VA servers, but to date there has been no plan identified that meets the high-level of 24/7 monitoring, technical support, and data security as the plan we are employing with the UM Computing Center. The UM Computing Center features physical security systems, including a single point of entry person trap and an enterprise-wide access control system. The access control system utilizes electronic identification cards with proximity technology and is continuously monitored by Health System Security Services. The Center was designed with redundancy in all critical systems to make concurrent maintainable operations possible. A state-of-the-art software firewall provides packet filtering, intrusion detection and protection, per user authentication and authorization, and multiprotocol routing. The SSL certificate in use on the website supports both 128-bit and 256-bit encryption, depending on the user's browser. Protection from outages is provided by redundant Uninterruptible Power System (UPS) units. The UPS units are provided with DC power by two redundant battery arrays located in separate rooms. The two 4 megawatt utility power feeds are backed up by emergency power provided by two 2.2 megawatt generators. All electrical systems have a minimum of N+1 redundancy. The system supports multi-level access control, which will allow us to limit access to the system on both the administrative and managerial sides. The VoIP service which makes possible automated calls is provisioned by the University of Michigan's enterprise-wide telecommunications provider, ITCom. SIP trunks, which manage the VoIP traffic, will initially be designed to handle over 50 simultaneous participant calls. The Medical School Information Services Solutions Center will coordinate the integration between applications and the communications infrastructure in collaboration with the VA ISO. Data transmitted to the server via IVR calls will use a website that will be protected by an SSL (Secure Sockets layer) certificate, ensuring that all data transmission between a user's browser and the web server are encrypted and are therefore secure. Additionally, all servers will be placed on a private network which can only be accessed by VPN or by being physically present behind the firewall. The Firewall, Nokia 2450 appliance, runs checkpoint VPN-1/Firewall-1 NGX (certified to EAL-4) providing stateful packet filtering, intrusion detection and protection, per-use authentication and authorization and multiprotocol routing. Alerts and notices are received from Checkpoint on any security vulnerability that may impact the firewall, which does contain IDS functionality, such as DOS protection. A separate IDS appliance exists that continually monitors for attacks 24/7. The workstation computers with server access are configured to require strong passwords in order to log-in, wake from sleep, or unlock screen savers.

The program website is separate from any electronic medical records or other data storage devices, and there will be no access to other participant-level PHI via the website or server. Participants in the research project will sign an informed consent document that details how their IVR data will be transmitted and stored. The website will be used only to store necessary data to initiate the IVR message notification calls to the participant. Information stored in the website's database will be limited to participants' study ID, year of birth, and telephone number, which will be entered through a web interface by study personnel only. All automated call data will be stored as numerical data, and the field names in the underlying database structure will be arbitrary so that the content of the files cannot be interpreted. All system files will be completely backed up in continuous 10 minute intervals to two physically separate secure locations. Database dumps are also performed every 8 hours, encrypted, and stored on an external BlueArc NAS system that is replicated between two sites. The database backups are kept for two weeks and the operating system files are kept for months.

Prior to commencing enrollment, we will establish a Data Transfer Agreements (DTA) with the Regents of the University of Michigan stipulating that sensitive information be transmitted solely over a Secure Sockets Layer (SSL) to encrypt the data first. The DTA will also require that the data on the server be capable of recovery and that copies of the data be stored in multiple secure locations. Lastly, it will specify that staff from MACC and MSIS cannot physically or electronically move the data from their site under any circumstances without our prior approval.

17.4 Potential Benefits

Potential benefits of the proposed research to the subjects and others
This study will evaluate an innovative method for improving informal care support through engaging patients' Care Partners and enhancing the effectiveness of their support. We expect this study to produce an evidence-based protocol and tools that engage VA patients with high-risk diabetes and their Care Partners in PACT to help PACT achieve the best diabetes outcomes. This protocol could then be implemented in PACT encounters with high-risk patients throughout the VA.
Many intervention-assigned participants are likely to experience direct benefits from participation in this study. All intervention-assigned patient participants will receive biweekly automated telephone assessment of their diabetes symptoms and self-care. This may improve the quality of their diabetes care, and their diabetes outcomes. Additionally, patient participants may experience additional benefits to their health and well-being as a result of feedback also being sent to the Care Partner whom they nominate. Coaching on patient activation skills may improve patient communication with their PACT clinicians, and result in improved diabetes clinical care. Because diabetes is prevalent and results in substantial morbidity, health care utilization and costs, we feel the study's potential benefits outweigh the low risk of minimal harm to participants.

Importance of the knowledge to be gained
We expect that this study will contribute to the growing body of knowledge on how VA can most effectively engage family supporters and Care Partners in patients' care to optimize health management and outcomes. By improving informal care support for Veterans with diabetes, this research may indicate new ways to improve their health, health-related quality of life, health behaviors, and reduce diabetes-related distress. Diabetes is prevalent among Veterans and often results in substantial morbidity, distress, disability, and health care costs. We feel the potential knowledge to be gained outweighs the low risk of minimal harm to participants.

18. Data and Safety Monitoring Plan
Institutional Review Board (IRB) approvals will be obtained at the [redacted] VAMC and the University of Michigan. The PI, Dr. Ann-Marie Rosland, will take ultimate responsibility for ensuring the safety of the participants. Regular study team meetings will be used to ensure that all data quality and IRB policies and procedures are being followed. This will include ensuring that (1) all participants understand, agree to, and sign a written consent form before participating; (2) strict adherence is maintained to communication regarding the participants' right to withdraw or refuse to answer questions; (3) staff maintain confidentiality both by protecting hard-copy and electronic data collection forms and also by avoiding all unauthorized conversations about individual patients; (4) consent forms and identifying information are kept separately from study related information about patients' sociodemographics, clinical characteristics, disease self-care, service use, and outcomes; (5) all identifying information is kept locked at all times and sensitive computer files are maintained on a secured VA server; (6) coding for ambiguous responses is handled in a way that is consistent and clear across data collectors and over time; and (7) participants are informed in writing how to contact the study PI, the study coordinator, and the relevant IRB office with any questions or concerns.

Dr. Rosland will be responsible for reporting all AEs that might arise during the course of the study to the [REDACTED]HS IRB. AEs during this study are defined as: "any experience that has taken place during the course of a research project, which, in the opinion of the investigators, was harmful to a subject participating in the research, increased the risks of harm in the research, or had an unfavorable impact on the risk/benefit ratio". AEs may be identified through participant report via the study toll-free number, or research staff viewing in medical records in the course of planned study activities. Specifically, life-threatening AEs will be reported to the [REDACTED]HS IRB by phone within 24 hours of the event with written notification to follow within 24 hours. Non-threatening potentially serious AEs that are causally related to the research (e.g. breach in confidentiality) will

be reported to the [REDACTED]HS IRB in writing within 48 hours. AEs and unanticipated problems that do not meet the definition of serious are to be reported to the study project manager as they are discovered, and will be reported to the [REDACTED]HS IRB in summary at the time of continuing review/project closure. Protocol deviations/violations that are likely to substantially adversely affect 1) the rights, safety, or welfare of a participant; 2) a participant's willingness to continue participation; or 3) the integrity of the research data, including VA information security requirements will all be reported to the [REDACTED]HS IRB within 5 working days of being made aware of the occurrence. All AEs and protocol deviations/violations will be brought to the immediate attention of the PI, regardless of categorization.

19. Inclusion of Women, Minorities and/or Children

Children will not be eligible for this study, as either patients or Care Partners. We will recruit eligible Veterans who give informed consent, regardless of their gender, race, or ethnicity. We expect the study to reflect the racial, ethnic, and gender distribution of the study sites. Representation of racial minority groups and women will be monitored throughout the project and if it appears that they are underrepresented among participants, significant efforts will be made to boost their enrollment. These efforts will include qualitative interviews with women and minorities who are and are not participating to understand barriers to participation and to learn new strategies for increasing representation of those groups.

References

1. Hibbard JH, Greene J. What the evidence shows about patient activation: better health outcomes and care experiences; fewer data on costs. *Health Aff Proj Hope*. 2013;32(2):207–214.
2. Rosland AM, Heisler M, Choi HJ, Silveira MJ, Piette JD. Family influences on self-management among functionally independent adults with diabetes or heart failure: do family members hinder as much as they help? *Chronic Illn*. 2010;6(1):22–33.
3. Silliman RA, Bhatti S, Khan A, et al. The care of older persons with diabetes mellitus: families and primary care physicians. *J Am Geriatr Soc*. 1996;44(11):1314–1321.
4. Rosland AM, Piette JD, Choi H, Heisler M. Family and friend participation in primary care visits of patients with diabetes or heart failure: patient and physician determinants and experiences.*Med Care*. 2011;49(1):37–45.
5. Wolff JL, Roter DL. Family presence in routine medical visits: a meta-analytical review. *Soc Sci Med 1982*. 2011;72(6):823–831.
6. Strom JL, Egede LE. The impact of social support on outcomes in adult patients with type 2 diabetes: a systematic review. *Curr Diab Rep*. 2012;12(6):769–781.
7. Luttik ML, Jaarsma T, Moser D, et al. The importance and impact of social support on outcomes in patients with heart failure: an overview of the literature. *J Cardiovasc Nurs*. 2005;20(3):162–9.
8. Lett HS, Blumenthal JA, Babyak MA, et al. Social support and coronary heart disease: epidemiologic evidence and implications for treatment. *Psychosom Med*. 2005;67(6):869–78.
9. Nicklett EJ, Heisler M, Spencer MS, Rosland AM. Direct Social Support and Long-term Health Among Middle-Aged and Older Adults With Type 2 Diabetes Mellitus. *J Gerontol B Psychol Sci Soc Sci*. 2013;68(6):933–943.
10. Gallant M. The influence of social support on chronic illness self-management: a review and directions for research. *Health Educ Behav*. 2003;30(2):170–195.
11. Hibbard JH, Greene J, Overton V. Patients with lower activation associated with higher costs; delivery systems should know their patients' "scores." *Health Aff Proj Hope*. 2013;32(2):216–222.
12. Hibbard JH, Stockard J, Mahoney ER, et al. Development of the Patient Activation Measure (PAM): conceptualizing and measuring activation in patients and consumers. *Health Serv Res*. 2004;39(4 Pt 1):1005–1026.
13. Carman KL, Dardess P, Maurer M, et al. Patient and family engagement: a framework for understanding the elements and developing interventions and policies. *Health Aff Proj Hope*. 2013;32(2):223–231.
14. Parchman ML, Zeber JE, et al. Participatory decision making, patient activation, medication adherence, and intermediate clinical outcomes in type 2 diabetes: a STARNet study. *Ann Fam Med*. 2010;8(5):410–417.
15. Giardina TD, King BJ, Ignaczak AP, et al. Root cause analysis reports help identify common factors in delayed diagnosis and treatment of outpatients. *Health Aff Proj Hope*. 2013;32(8):1368–1375.
16. Hibbard JH, Cunningham PJ. How engaged are consumers in their health and health care, and why does it matter? *Res Brief*. 2008;(8):1–9.
17. Maeng DD, Martsolf GR, Scanlon DP, et al. Care coordination for the chronically ill: understanding the patient's perspective. *Health Serv Res*. 2012;47(5):1960–1979.
18. Kinnersley P, Edwards A, Hood K, et al. Interventions before consultations to help patients address their information needs by encouraging question asking: systematic review. *BMJ*. 2008;337:a485.
19. Deen D, Lu W-H, Rothstein D, et al. Asking questions: the effect of a brief intervention in community health centers on patient activation. *Patient Educ Couns*. 2011;84(2):257–260.
20. Greenfield S, Kaplan SH, Ware JE Jr, et al. Patients' participation in medical care: effects on blood sugar control and quality of life in diabetes. *J Gen Intern Med*. 1988;3(5):448–457.
21. Kahwati LC, Lance TX, Jones KR, et al. RE-AIM evaluation of the Veterans Health Administration's MOVE! Weight Management Program. *Transl Behav Med*. 2011;1(4):551–560.
22. Connell CM. Psychosocial contexts of diabetes and older adulthood: reciprocal effects. *Diabetes Educ*. 1991;17(5):364–71.
23. Rosland AM, Heisler M, Janevic MR, Connell CM, Langa KM, Kerr, EA, Piette JD. Current and potential support for chronic disease management in the United States: the perspective of family and friends of chronically ill adults. *Fam Syst Health J Collab Fam Healthc*. 2013;31(2):119–131.
24. Piette JD, Rosland AM, Silveira M, et al. The case for involving adult children outside of the household in the self-management support of older adults with chronic illnesses. *Chronic Illn*. 2010;6(1):34–45.
25. Wolff JL, Roter DL. Hidden in plain sight: medical visit companions as a resource for vulnerable older adults. *Arch Intern Med*. 2008;168(13):1409–1415.

26. Janevic MR, Rosland AM, Wiitala W, et al. Providing support to relatives and friends managing both chronic physical illness and depression: the views of a national sample of U.S. adults. *Patient Educ Couns*. 2012;89(1):191–198.
27. Anderson ES, Wojcik JR, Winett RA, et al. Social-cognitive determinants of physical activity: the influence of social support, self-efficacy, outcome expectations, and self-regulation among participants in a church-based health promotion study. *Health Psychol*. 2006;25(4):510–20.
28. Coffman MJ. Effects of tangible social support and depression on diabetes self-efficacy. *J Gerontol Nurs*. 2008;34(4):32–9.
29. DiIorio C, Shafer PO, Letz R, et al. Project EASE: a study to test a psychosocial model of epilepsy medication management. *Epilepsy Behav*. 2004;5(6):926–36.
30. Cotter KA, Sherman AM. Love hurts: the influence of social relations on exercise self-efficacy for older adults with osteoarthritis. *J Aging Phys Act*. 2008;16(4):465–83.
31. Rosland AM, Kieffer E, Israel B, Cofield M, Palmisano G, Sinco B, Spencer M, Heisler M. When is social support important? The association of family support and professional support with specific diabetes self-management behaviors. *J Gen Intern Med*. 2008;23(12):1992–1999.
32. Nicklett EJ, Liang J. Diabetes-Related Support, Regimen Adherence, and Health Decline Among Older Adults. *J Gerontol Ser B-Psychol Sci Soc Sci*. 2009.
33. Clayton JM, Butow PN, Tattersall MHN, et al. Randomized controlled trial of a prompt list to help advanced cancer patients and their caregivers to ask questions about prognosis and end-of-life care. *J Clin Oncol Off J Am Soc Clin Oncol*. 2007;25(6):715–723.
34. Piette JD, Rosland AM, Marinec NS, et al. Engagement with automated patient monitoring and self-management support calls: experience with a thousand chronically ill patients. *Med Care*. 2013;51(3):216–223.
35. Rohrbaugh MJ, Shoham V, Coyne JC, et al. Beyond the "self" in self-efficacy: Spouse confidence predicts patient survival following heart failure. *J Fam Psychol*. 2004;18(1):184–193.
36. Rosland AM, Piette JD. Emerging models for mobilizing family support for chronic disease management: a structured review. *Chronic Illn*. 2010;6(1):7–21.
37. Griffin JM, Meis L, Greer N, et al. *Effectiveness of Family and Caregiver Interventions on Patient Outcomes Among Adults with Cancer or Memory-Related Disorders: A Systematic Review*. Washington (DC): Department of Veterans Affairs; 2013. Available at: http://www.ncbi.nlm.nih.gov/books/NBK148475/. Accessed December 5, 2013.
38. Dunbar SB, Clark PC, Deaton C, et al. Family education and support interventions in heart failure: a pilot study. *Nurs Res*. 2005;54(3):158–66.
39. Burke JA, Earley M, Dixon LD, et al. Patients with diabetes speak: exploring the implications of patients' perspectives for their diabetes appointments. *Health Commun*. 2006;19(2):103–14.
40. VHA Patient Centered Medical Home Model (PACT) Concept Paper. 2011.
41. Kerr EA, Lucatorto MA, Holleman R, et al. Monitoring performance for blood pressure management among patients with diabetes mellitus: too much of a good thing? *Arch Intern Med*. 2012;172(12):938–945.
42. Pentakota SR, Rajan M, Fincke BG, et al. Does diabetes care differ by type of chronic comorbidity?: An evaluation of the Piette and Kerr framework. *Diabetes Care*. 2012;35(6):1285–1292. doi:10.2337/dc11-1569.

FINAL STUDY PROTOCOL

Title: Engaging Veterans and Family Supporters in PACT to Improve Diabetes Management

Funding Source: VA Health Services Research and Development

Principal Investigator: Ann-Marie Rosland, MD MS

Final Protocol Version: September 29, 2016

**This version approved by IRB on 9.8.17**

List of Terms and Abbreviations

[REDACTED]VA-[redacted] VA
ADL – Activities of Daily Living
AE- Adverse Events
BMI – Body Mass Index
BP – Blood pressure
Care Partner (CP)– Unpaid family member or friend who is involved in the patient's health care
CBOC – Community Based Outpatient Clinic
CO-IMPACT - Caring Others Increasing EngageMent in PACT intervention
CPRS – Computerized Patient Record System
DEC – Dyad Engagement Coach
ED – Emergency Department
HbA1C – Glycated hemoglobin (hemoglobin A1c)
HIPAA - Health Insurance Portability and Accountability Act
IVR- Interactive Voice Response
LDL – low-density lipoprotein
PACE - Patient and Caregiver Experiences with Diabetes Study
PACT- Patient Aligned Care Teams
Patient Partner – Patient participants who are paired with a Care Partner
PCP – Primary Care Physician
PHI - Protected Health Information
RCT – Randomized Controlled Trial
SAS - Statistical Analysis Software
SBP – systolic blood pressure
SD - Standard Deviation
UKPDS – United Kingdom Prospective Diabetes Study
VA – Veterans Affairs
[REDACTED]HS - VA [redacted] Healthcare System ([redacted] VA + CBOCs)
VHA – Veterans Health Administration

1. Abstract

BACKGROUND: Veterans with diabetes must control cardiovascular risk factors in order to prevent disabling and life-threatening complications. The VA PACT initiative seeks to provide patients comprehensive support for following diabetes care regimens, but Veterans must effectively engage in and navigate care to obtain the most benefit from PACT. One relatively untapped resource for supporting engagement in PACT is patients' family and friends ("Care Partners").

OBJECTIVES: The *overall objective* of this randomized trial is to test a strategy to strengthen the capacity of supporters to help patients with high-risk diabetes engage in PACT care and successfully enact care plans. The *central hypothesis* is that providing health care engagement tools to both Care Partners and patients will increase patient activation and improve management of diabetes complication risks.

RESEARCH PLAN: This will be a randomized controlled trial evaluating an intervention (Caring Others Increasing EngageMent in PACT, or CO-IMPACT) designed to structure and facilitate Care Partner involvement in PACT so that patients can become more actively engaged in PACT care, and improve their diabetes treatment processes and outcomes.

METHODS: 240 patients with diabetes receiving PACT primary care who 1) are at high risk for diabetes complications due to hyperglycemia OR high blood pressure and 2) have a Care Partner involved in their care will be recruited along with their Care Partner. Patient-supporter dyads will be randomized to the CO-IMPACT intervention or usual PACT care for high-risk diabetes, for 12 months. The CO-IMPACT protocol provides patient-supporter dyads: one coaching session on action planning, communicating with providers, navigation skills and support skills; preparation by phone before patients' primary care visits; after-visit summaries for both patients and Care Partners; and biweekly automated phone calls to prompt action on new patient health concerns. CO-IMPACT builds on medical record-integrated patient activation tools in the PACT toolkit and is designed to be implementable within existing PACT nurse encounters. Primary outcomes for this study include a validated measure of patient activation (Patient Activation Measure-13) and a cardiac event 5-year risk score designed for patients with diabetes (UKPDS Risk Engine). Secondary outcomes include patients' self-efficacy for diabetes self-care; diabetes self-management behaviors including medication adherence; diabetes distress; and glycemic and blood pressure control. Measures among supporters will include supporter activation, use of effective support techniques, distress about patient's diabetes care, and Care Partner burden. We will also measure patient-supporter and patient-provider relationship quality, patient safety (e.g. hypoglycemia), and utilization. We will measure potential moderators of intervention effect, such as patient health literacy level, and facilitators and barriers to wider implementation among participants and staff.

2. Study Personnel

Principal Investigator/Study Chair:

Ann-Marie Rosland, MD, MS
Research Investigator, VA Ann Arbor Healthcare System
Address: NCRC (152), 2800 Plymouth Rd., Bldg 16, 3rd Floor, Ann Arbor, MI 48105
Phone: (734) 845-3504
Email: ann-marie.rosland@va.gov

Co-Investigators:

Michele Heisler, MD, MPA
Research Investigator, VA Ann Arbor Healthcare System
Address: NCRC (152), 2800 Plymouth Rd., Bldg 16, 3rd Floor, Ann Arbor, MI 48105
Email: mheisler@umich.edu

Eve Kerr, MD, MPH
Research Investigator, VA Ann Arbor Healthcare System;
Address: NCRC (152), 2800 Plymouth Rd., Bldg 16, 3rd Floor, Ann Arbor, MI 48105
Email: Eve.Kerr@va.gov

John Piette, PhD, MSc
Research Investigator, VA Ann Arbor Healthcare System
Address: NCRC (152), 2800 Plymouth Rd., Bldg 16, 3rd Floor, Ann Arbor, MI 48105
Email: jpiette@umich.edu

Ranak Trivedi, PhD, MA, MS
Research Investigator, VA Palo Alto Healthcare System
Address: VA Palo Alto Health Care System, Menlo Park Division, 795 Willow Road, Menlo Park, CA 94025
Email: Ranak.Trivedi@va.gov

Study Staff:

Shelley Stoll MPH
Project Manager, Center for Clinical Management Research
Address: NCRC (152), 2800 Plymouth Rd., Bldg 16, 3rd Floor, Ann Arbor, MI 48105
Email: Shelley.Stoll@va.gov

Caroline Keep
Research Associate, Center for Clinical Management Research
Address: NCRC (152), 2800 Plymouth Rd., Bldg 16, 3rd Floor, Ann Arbor, MI 48105
Email: Caroline.Keep@va.gov

Dana Horowitz
Research Associate, Center for Clinical Management Research
Address: NCRC (152), 2800 Plymouth Rd., Bldg 16, 3rd Floor, Ann Arbor, MI 48105
Email: dana.horowitz@va.gov

Jenny Davis MHSA
Data Manager, Center for Clinical Management Research
Address: NCRC (152), 2800 Plymouth Rd., Bldg 16, 3rd Floor, Ann Arbor, MI 48105
Email: Jennifer.Davis@va.gov

3. Rationale

Despite system wide advances in diabetes quality of care, over 30% of VHA patients with diabetes have high blood pressure, hyperglycemia, and hyperlipidemia and thus are at high-risk for disabling diabetes complications. To reduce diabetes complications, these 'high-risk' veterans are advised to follow treatment regimens that are complicated and often difficult to follow. PACT (Patient-Aligned Care Teams) seeks to provide patients comprehensive, team-based support for following diabetes care regimens. PACT's success, however, hinges on its ability to effectively engage patients in care. It is widely appreciated that patients who are more engaged in their health care have better health outcomes.[1] To fully engage in PACT, veterans must effectively communicate with multiple PACT team members and proficiently navigate the health care system. High-risk patients, with more complex care needs, often need more support to engage in what PACT has to offer.

One relatively untapped resource for this support is patients' family and friends. Three out of four adults with diabetes reach out to an unpaid family member or friend (a Care Partner) for ongoing help with diabetes managemen.[2,3] Half regularly bring a Care Partner to their medical appointments.[4,5] Chronically ill patients with Care Partners have better self-management and long-term health outcomes.[6–9] However, while PACT emphasizes the importance of family members as part of the care team, <u>PACT does not have formal mechanisms to involve Care Partners in PACT care</u>. This is unfortunate as these supporters could play a crucial role in helping patients effectively engage in PACT care. Our preliminary work shows that 25-50% of Care Partners already regularly talk with PACT providers on the phone, try to help patients prepare for PACT appointments, and try to help patients carry out plans made at their medical visit. However, studies indicate that Care Partners are currently less effective at influencing patients' medical self-management tasks (e.g., medication adherence or blood glucose monitoring) than healthy lifestyles (e.g., healthy eating).[10] Care Partners tell us they need more information on patient's medical care plans, clear channels for communicating with PACT team members, and information on navigating PACT resources.

4. Research Problem or Question

Our *<u>long-term goal</u>* is to provide VA clinical teams with evidence-based structured approaches to communicating with Care Partners that improve patient health outcomes and satisfaction with care. The *<u>objective</u>* of this randomized trial is to test a strategy to strengthen the capacity of supporters to help patients with high-risk diabetes engage in PACT care and successfully enact care plans. Our *<u>central hypothesis</u>* is that providing health care engagement tools to both Care Partners and patients will increase patient activation and improve management of diabetes complication risks.

5. Specific Aims and Primary Measures

The study will address the following *<u>specific aims</u>*:

4) Determine the effect of the CO-IMPACT intervention on engagement in treatment and health behaviors among patients at high-risk for diabetes complications. *We hypothesize that CO-IMPACT will significantly increase patient activation, as measured by the PAM-13, compared to usual PACT care.*

5) Determine the effect of the CO-IMPACT intervention on health risks among patients at high-risk for diabetes complications**.** *We hypothesize that CO-IMPACT will significantly decrease patients' 5-year cardiovascular event risk, as measured by the UKPDS cardiac risk score (which includes HbA1C,non-fasting lipid levels and blood pressure), compared to usual PACT care.*

6) Evaluate how the characteristics of patients, family supporters, and their relationships mediate and moderate the effects of CO-IMPACT. *We hypothesize that higher levels of family supporter participation in CO-IMPACT will lead to greater improvements in patient activation and patient cardiac risk.*

6. Background

<u>Many VHA Patients With Diabetes Are At High Risk For Diabetes Complications:</u>

Twenty-five percent of VHA patients have diabetes, representing about 1.5 million Veterans. While the quality of diabetes care is high when measured by processes such as HbA1c monitoring and lipid testing, 20-30% of VHA diabetes patients have poor glycemic control (HbA1c >8%), poor blood pressure control (>140/90), or high lipid levels (LDL >130 mg/dl).

Increasing Patient Activation Can Improve Diabetes Management:
"Activated" patients are those who have the "skills and confidence to become less activated in their health and healthcare."[11] Activation includes the ability to share in decision-making with health care providers, monitor and self-manage symptoms, and access care in an appropriate and timely way. Patient activation encompasses several more specific health behavior concepts, including locus of control and self-efficacy for executing self-managing behaviors.[12] The main result of patient activation is patient engagement, or "actions that people take for their health and to benefit from care."[13]

Highly activated patients have better health behaviors (including adherence to medications, regular self-monitoring at home, physical activity, and healthy eating) and health outcomes (including lower BMI, HbA1C, blood pressure, and cholesterol).[1] Increases in activation over time are linked to improvements in similar health behaviors and outcomes.[14] Less activated patients are unengaged in their care, delay care seeking and have poorly coordinated outpatient follow-up, including in the VHA.[15–17] Less activated patients also have higher rates of hospitalizations and ED visits,[1] and higher costs of care.[11]

Intervention studies have shown that coaching or prompting patients to ask questions immediately prior to a medical visit can increase patient activation during and just after the visit.[18,19] Most of these studies have been done in oncology clinics, but one RCT of pre-visit coaching to increase diabetes patients' information gathering led to improved glycemic control and less reported functional limitations compared to control patients.[20] How best to increase global patient activation among patients with chronic illness is still uncertain.

Full Patient Engagement in PACT Diabetes Management Requires High Activation:
The VA Patient Aligned Care Teams (PACT) model for primary care is designed to provide multiple, coordinated mechanisms for supporting patients with complex chronic disease. These include visits with nurse care managers and clinical pharmacists that complement primary care provider (PCP) visits, telephone visits between in-person visits, health psychology programs to support self-management behavior change, group diabetes education, the MOVE! weight management program, and telehealth monitoring. To make the most of this complex array of new services, patients must identify resources that can best meet their needs, make appointments or enroll in programs, actively participate, implement care plan changes, and maintain ongoing communication with clinical teams. Uptake of PACT chronic disease management programs has been slow,[21] and it is uncertain what approach is best to help complex patients obtain the full benefit of PACT chronic disease care. *In our CO-IMPACT (Caring Others Increasing EngageMent in PACT) intervention, we will mobilize patients' family members and friends to help increase patient engagement in PACT care.*

Most VA Patients with Diabetes Have Family Members or Friends Who Are Involved in Their Health Care:
As many as 75% of VA patients with diabetes have a family member or friend who is regularly involved in their diabetes care (a 'Care Partner').[2,3] These supporters assist patients in engaging in activities directly related to successful diabetes management, including medication management and adherence, tracking home glucose and blood pressure measurements, maintaining a healthy eating plan, and being physically active.[2,3,22] Care Partners often help patients make key decisions about their diabetes management, such as how to address medication side effects.[23] Typically, 50-60% of Care Partners are spouses, and most of the rest are family members who do not live with the patient (such as adult children).[2,24,25]

Care Partners Can Affect Chronic Disease Management and Outcomes:
Family and friend support can lead to better glycemic control and lower mortality among patients with diabetes.[6] In other chronic conditions that require significant self-management, such as cardiac disease and heart failure, social support is linked to lower rates of recurrent cardiac events and hospitalizations.[7,8] There is strong evidence that social support acts on chronic disease outcomes largely through improved patient self-management behaviors.[10]

There is little direct evidence that Care Partner engagement can increase overall patient activation, but we have

several reasons to hypothesize that this is the case. There are very strong links between social support and improved patient self-efficacy for self-care, [26–30]a concept closely related to patient activation. Higher social support is linked to activated self-management behaviors, such as increased self-monitoring.[31,32] When supporters accompany patients to medical visits, patients exhibit more activated behavior, including increased participation in decision making with providers.[4,5,25] In one pre-visit preparation intervention delivered to patients with cancer and their visit companions, both patient and companion question asking increased.[33] We found in prior work that patients participating with a Care Partner in an interactive voice response self-management intervention were more engaged in the intervention than those who participated alone.[34,35]

Previous interventions aiming to leverage family support to improve disease management have generally engaged supporters in patients' day-to-day health management through counseling or coaching.[36,37] Such interventions have demonstrated improvements in dietary behavior among heart failure patients,[38] and physical activity among obese patients.[39] However no published interventions or known clinical programs have focused on helping Care Partners boost chronically ill patients' engagement in clinical care and medical self-care (i.e. medication adherence).

Significance of Proposed Research:
Veterans with high-risk diabetes are highly vulnerable to disabling complications and death. These patients often remain at high risk for poor outcomes and frequently use emergency care. Innovative and sustainable approaches that increase engagement of patients' and their supporters in primary care could improve patients' risk factor control, and reduce emergency utilization and morbidity.
The VA has recently expanded its commitment to engaging family caregivers in medical care. The Caregivers and Veterans Health Services Act of 2010 provides Veterans' caregivers with substantial support through several means, including increased training and financial support; a telephone support line; a VA website with caregiving tools and resources, and full-time Caregiving Program Coordinators at each VA facility nationwide.

An overarching goal of the nationwide VA PACT initiative is to engage the patient, and all those helping to care for the patient, in a coordinated, team-based approach.[40] The PACT model specifically includes family members as part of the care team. Structured and implementable approaches to identifying Care Partners and including them in the flow of health care information are needed for PACT to achieve this goal. If successful, this study will produce a scalable protocol that can be used by VA PACT teams across the VHA to engage Veterans' Care Partners and caregivers in diabetes care. The lessons learned in this study can be used to evaluate and enhance Care Partner and caregiver engagement for patients with other complex conditions, or patients in vulnerable situations such as transitions from hospital to home.

7. Preliminary Data

Ann Arbor VA Patients with High-Risk Diabetes Have Involved Care Partners: In our prior studies, including our preliminary VA observational studies of Patient and Caregiver Experiences (PACE) with Veterans with high-risk diabetes and their Care Partners, we found that 40% of out-of-home supporters live within 20 miles of the patient's home, and 78% talk with the patient by phone at least weekly.[24] Prior research, including our own, has shown that chronically ill patients with low health literacy, multiple comorbidities, and comorbid depression involve Care Partners in their care more often.[4,5,26]
Our prior studies also indicate that Care Partners are highly involved in patients' interactions with the health care system. About half of patients with diabetes are regularly accompanied by a supporter into the exam room for primary care visits,[4,5] and 25% have had a supporter talk on the phone with their clinician in the last year.[4] Importantly, (Table 1) we found that Care Partners often help patients prepare questions before visits, assist patients in processing visit information and plans ('debriefing'), and help patients navigate VA services such as pharmacy fills and diabetes class enrollment. *Thus, there is significant potential to increase patient engagement in PACT care by enhancing the effectiveness of these interactions among Care Partners, patients, and PACT team members at key points in medical care.*

Care Partners are Limited By Lack of Patient-Specific and VA-Specific Information, and Structured Support Opportunities: In our national survey of 760 family supporters of patients with chronic disease,[41] and in our 12 VA PACE supporter interviews, supporters reported feeling limited by a lack of patient-specific information, such as changes in medication regimens or test results, as well as a lack of health system-specific information, such as the

roles of PACT teamlet members or available diabetes programs. Supporters also face significant challenges when helping patients prepare for, and debrief after, clinical visits. For example, as shown in Table 1, patients often do not bring written questions for the doctor, and many are not confident they are reporting accurate visit information back to their supporter. Twenty-eight percent of supporters reported that their patient-partner regularly discusses being confused about health care provider instructions.[23] In PACE interviews, many supporters requested printed summaries after patient visits with a clear way to follow-up with questions. Importantly, few patients (9%) in PACE surveys felt that privacy should be a barrier to information sharing between supporters and patients' clinicians. However in interviews, several supporters reported feeling intimidated by perceived complexity in VA primary care clinic structure and privacy rules.
Supporter effectiveness could also be boosted through more structured and action-oriented between-visit discussions with patients. In our national Care Partner survey, we found that supporters discuss health with their patient-partners almost every time they talk, but approximately 30% were unsure what questions to ask or what advice to give about diabetes. Supporters can make the most of these discussions when they have patient-specific information and when they use evidence-based support techniques,[42] such as positive and autonomy-supportive statements, and collaborative action planning and coping.

Pilot of CO-IMPACT Shows Feasibility and Perceived Benefit: In preparation for the current trial, we developed the CO-IMPACT intervention protocol, patient and supporter materials, and assessment instruments, and delivered CO-IMPACT (see Section 8, Table 1) to 19 patient-supporter dyads over a 4-month period. Patient participants were recruited from a [REDACTED]HS registry of patients with high-risk diabetes with similar criteria to section D3. 18/19 patients were men, with a mean age of 66 years (range 47-89). Patients chose a Care Partner to participate with them, who was assessed for eligibility as described in D4. Eighteen of 19 supporters were women; mean age of supporters was 54 years (range 22-71). Most supporters were spouses (N 11) and lived with the patient. The other 8 supporters did not live with the patient (7/8 lived ≤20 miles apart) and included 3 daughters, 1 son, 3 friends, and 1 other family member. All patient-supporter dyads completed an initial session with a Dyad Engagement Coach(DEC). Among the 19 patients, 21 pre-visit preparation phone calls were completed (out of 25 eligible visits), and 25 after-visit summaries were mailed to dyads. Patients completed 82% of attempted weekly automated IVR telephone assessments, with 18/19 patients continuing to complete IVR calls for the entire 4 months. At follow-up, 95% of patients and 89% of supporters said they were satisfied with the program. 84% of patients felt CO-IMPACT helped them more effectively manage diabetes, and 84% of supporters felt the program helped them more effectively support the patient's health care. 100% of patients would recommend the program to another Veteran. In post-intervention interviews, both patients and supporters reported that CO-IMPACT was helpful in promoting patient engagement and changing patient-supporter communication about care: "She [supporter] reminded me [patient] to call my doctor with problems", "He [patient] brought our questions with him to his appointment and asked them to the doctor and nurse", "I [supporter] am more aware of what questions to ask him [patient] and what to say about diabetes". Extensive interviews with participants and their PACT teamlets have informed intervention refinement.

8. Overview of Study Design:

This will be a randomized controlled trial evaluating an intervention (CO-IMPACT) designed to activate dyads of Care Partners and Veterans with diabetes. 240 patients with diabetes receiving PACT primary care who are at high risk for diabetes complications and who have a Care Partner involved in their care will be recruited along with a Care Partner.

The overarching goal of this intervention is to structure and facilitate Care Partner involvement in PACT so that patients can become more actively engaged in PACT care, and improve their diabetes treatment processes and outcomes. CO-IMPACT will address key limitations to supporter effectiveness by providing supporters with information about their patient-partner's health status and treatment plan, ways to effectively identify and engage in PACT-related services, structured pre, post, and between visit information that can improve supporter-patient discussions about diabetes plans, and guidance to supporters on evidence-based communication techniques. Patient-supporter dyads will be identified via a [REDACTED]HS registry of patients with high-risk diabetes and randomized to CO-IMPACT or usual PACT care. Main outcomes will be measured at baseline, six months and 12 months post-enrollment via patient and supporter surveys, and patient laboratory tests, vital signs, and medical records. The below chart is a depiction of the overall study design.

Table 1: Study Design

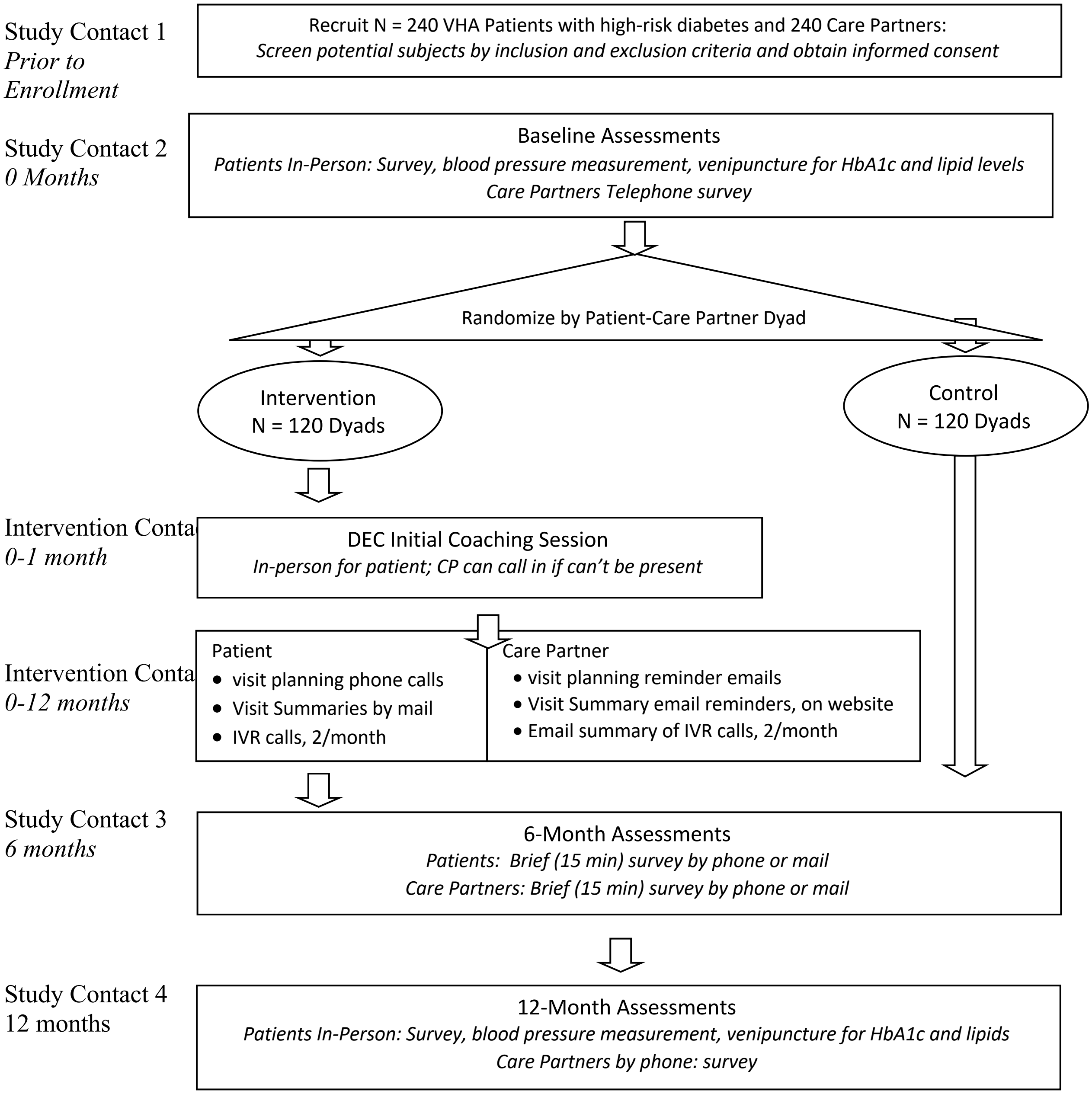

9. Experimental Plan

9.1 Study Inclusion and Exclusion Criteria

9.1.1 Participant Inclusion Criteria

Patient Inclusion criteria:

- Provide signed and dated informed consent form
- Willing to comply with all study procedures and plan to be available for the duration of the study

- Validated though completion of patient study screener:
  - Plan to use Ann Arbor VA Healthcare System (including CBOCs) primary care as their main source of diabetes care over the subsequent 12 months
  - Able to use telephone to respond to twice monthly automated IVR calls
  - Be able to identify an eligible adult family member or friend who is regularly involved in their health management or health care (get involved with medications, managing sugars, coming to appointments, etc) who consents to participate in the study
- Validated through patient medical record:
  - Male or female, age 30-70 years old
  - Have a diagnosis of diabetes, defined as:
  - (1) a diagnosis of diabetes based on encounter diagnoses from 1 inpatient or 2 outpatient encounters (ICD9 code of 250.xx, 357.2x, 362.xx, 366.41, 962.3 or E932.3 OR ICD10 code of E08.xx, E09.xx, E10.xx, E11xx, E13.xx, O24.0xx, or O24.1xx) OR a diabetes medication (at least one >3 month prescription from VA drug classes HS501 (insulin) or HS502, other than metformin),
  - Have an assigned NON-RESIDENT and NON-GeriPACT [REDACTED]HS (all 4 sites) primary care provider
  - Have 2 or more qualifying in-person primary care visits at [REDACTED]HS in the last 12 months:

- Be at high-risk for diabetes complications, defined as:
  - Poor glycemic control, defined as last HbA1C >8 within the last 9 months

    OR

  - Poor blood pressure control, defined as:
    - (If they have >1 BP in last 9 months) Most recent SBP>=150 and mean SBP over 9 months >=150
    - (If only 1 BP in last 9 months) Last SBP in last 6 months >=160 and no other BP measures
    - AND exclude from poor BP control group if last BP diastolic or mean diastolic is <=65
    - Notes on BP data to use:
      - If multiple BPs on one day, use the lowest one.
      - Exclude BPs done on days with these encounters: ED, urgent care, procedure or surgery department encounters; inpatient days.

        Specific Stop codes for BPs to EXCLUDE from the data:
        102 ADMITTING/SCREENING; 110 Interventional Radiology; 130 (ER); 131 (Urgent Care); 158 Brachytherapy; 321 GI Endoscopy

        327 THROUGH 333 Procedures including Cardiac Catheterization; 401 (gen surg); 402 (cardiac surgery); 403 (ENT); 405 (hand surg); 406 (neurosurg); 407 (Opth); 409 (orthopedics)

        410 (plastic surg); 412 (proctology); 413 (thoracic surg); 414 (urology); 415 (dialysis access, vascular surg); 418 (amputation); 419 (anethsesia pre-op); 426 (women's surgery); 427 Anethsesia Special Procedures in operating room suite; 429 (patient care in OR); 430 (cysto room); 431

(chemotherapy); 434 (non-OR anesthesia procedures); 435 (surgical procedure unit)

Care Partner Inclusion Criteria:

- Validated through completion of Care Partner study screener
    - Discusses patient's health issues at least twice monthly
    - At least 21 years of age
    - Fluent in English
    - Expect to have either continuous postal mail service or internet access
    - Live in the United States

Patient Exclusion Criteria:

- Validated through completion of patient study screener
    - Expect to have >1 month gap in [REDACTED]HS care in the 12 months following enrollment (e.g. snowbird travel).
    - Plan to receive the majority of their care for diabetes mainly from a non-PACT provider (either VA specialist or nonVA provider) in the 12 months following enrollment
    - Live in a nursing home OR assisted living
    - Have significant cognitive impairment as measured by more than 2 of 6 possible errors on the Callahan Six-item Screener to Identify Cognitive Impairment
    - Need help with more than 1 of the 6 basic ADLs as measured by the Katz Basic Activities of Daily Living Scale
    - Do not speak English
    - Have a life-limiting severe illness (such as ESRD requiring dialysis, COPD requiring oxygen, cancer undergoing active treatment, receiving palliative/hospice care)
    - Are concurrently enrolled in another research study, at time of enrollment, that could conflict with CO-IMPACT's protocol (e.g. another diabetes management research intervention)
    - Concurrently enrolled in Diabetes TeleHealth (CCHT)
    - Do not have a working phone or are not able to use a telephone to respond to automated IVR calls
    - Currently Pregnant or planning to become pregnant in the next 12 months
  - Validated through patient medical record:
- Have a serious mental illness, dementia, or active substance abuse issue as determined by the following encounter codes in a single inpatient or outpatient encounter in the last two years.

	ICD9 Codes	ICD10 Codes
Schizophrenia/Delusional Disorders/Other Psychoses	295.0x, 295.1x, 295.2x, 295.3x, 295.4x, 295.6x,295.7x,295.8x,295.9x, 297.0x,297.1x,297.2x,297.3x,297.8x,297.9x, 298.0x, 298.1x, 298.2x, 298.3x, 298.4x, 298.8x,298.9x,	F20.xx-F29.xx F06.0x, F06.1x, F06.2x
Bipolar:	296.0x, 296.1x, 296.4x, 296.5x, 296.6x, 296.7x, 296.8x	F30.xx, F31.xx, F44.xx, F45.xx
Substance abuse	303.xx (alcohol dependence syndrome), 304.xx (drug dependence), 305.xx (excluding 305.1 – tobacco use disorder), 291.xx (alcohol induced mental disorders), 292.xx (drug induced mental disorders)	F10.xx, F11.xx, F13.xx, F14.xx, F15.xx, F16.xx, F18.xx, F19.xx
Dementia	'0461x', '0463x', '2900x', '2903x', '2912x', '3310x', '3311x', '29010', '29011', '29012',	F01.xx, F02.xx, F03.xx, G30.xx,

	'29013', '29020',29021', '29040','29041','29042','29043', '29410', '29411', '33111', '33119', '33182'	G31.0x, G31.1x, G31.2x, G31.81, G31.82, G31.83, G31.85
Moderate, severe, and profound intellectual disability		F71.xx, F72.xx, F73.xx

Care Partner Exclusion:

- Validated through completion of Care partner study screener
    - Receive pay for caring for the patient
    - Have significant cognitive impairment as measured by more than 2 of 6 possible errors on the Callahan Six-item Screener to Identify Cognitive Impairment
    - A self-report of a physician diagnosis of dementia, Alzheimer's, schizophrenia, or manic depression
    - Need help with basic ADLs as measured by the Katz Basic Activities of Daily Living Scale (score less than 5)
    - Have a life-limiting severe illness (such as end-stage renal disease requiring dialysis, chronic lung disease requiring oxygen, cancer undergoing active treatment, receiving palliative/hospice care)

9.2 Recruitment

We will recruit 240 pairs, or dyads, of Care Partners and patients receiving care for diabetes, who are also at high-risk for diabetes complications, in the one parent facility and three CBOCs affiliated with the VA Ann Arbor Healthcare System ([REDACTED]AHS).

Prior to commencing recruitment activities in a site (Ann Arbor VA Hospital or one of three CBOCs), study team members will attend a staff meeting of PACT primary care providers at the site during which they describe the study and how their patients may be involved. Time will be given for providers to ask questions, and providers will be given the option to make the patients assigned to their primary care panel ineligible for the study. Following these meetings, providers will receive via email a summary of the study information provided at the staff meeting and the ability to opt their patients out of the study by replying to the email within one week of its receipt.

9.2.1 Patient Recruitment

Following the provider "opt-out" window, potentially eligible patients will be identified using the VA patient database (Corporate Data Warehouse), with the specific criteria noted above. They will be sent an introductory letter informing them about the study and inviting them to learn more about participating. The letter will include a study phone number and language indicating that they can opt out of further contact by calling study staff via a toll-free number or by mailing a form that is printed on the back of the letter in a postage-paid, addressed envelope that is included with the letter. The outside of the envelope will not contain anything that indicates the nature of the research. In the absence of such notification, 7-10 days after the letter is expected to arrive, study staff will call patients to explain the study in more detail, conduct initial screening and if eligible, ask if they wish to participate. During the initial contact call, the study will be described and patients will have ample and repeated opportunities to ask questions.

9.2.2 Care Partner Recruitment

During the initial screening phone call to the patient, willing and eligible patients will be asked to identify a Care Partner to participate in the study with them. Eligible patients will be encouraged to contact the potential Care Partner to explain their interest in the study, and concurrently, the RA will send a letter to the potential Care Partner that includes a study information sheet. After about a week, the RA will call the potential Care

Partner to screen for interest and eligibility. If the individual is interested and eligible, they will be asked for verbal consent following a clear protocol. We anticipate that in some rare cases, the patient will not have the potential Care Partner's mailing address or email address; in such cases, study staff will send the materials to the patient and ask that they deliver them to the prospective Care Partner. After a waiting period determined by the patient, the study staff will call the potential Care Partner.

9.3 Enrollment

9.3.1 Patient Participants

At in-person enrollment, study staff will describe the content of the study in detail, including that patients and Care Partners can decline participation in the study at any time or decline Care Partner participation in the patient's health care at any time. Patients will also be told in detail the type of clinical information that Care Partners may have access to should they be randomized to the intervention group. After any and all patient questions are encouraged and answered, and informed consent and HIPAA is obtained via signature, the RA will conduct the baseline assessment, including survey and BP measurement. The RA will ensure that each patient has a glucometer, glucometer supplies, and home blood pressure cuff, if desired by the patient.

The RA will also enter the order for lab work at the VA clinical laboratory to assess HbA1c and non-fasting lipid panel. Details on baseline assessments can be found in Section 12, Data Collection Procedures.

9.3.2 Care Partner Participants

Oral informed consent via phone will be obtained from Care Partner participants. The Care Partner will have received a written study information sheet, and the RA will follow a script to go over all key points in the consent form, answer any questions, and request oral consent. The RA will document the process, as specified in the script. After informed consent is obtained, he/she will be asked to complete a baseline survey assessment by phone.

9.4 Randomization Procedures

After both baseline assessments are completed for the dyad, the dyad will be randomly assigned, within blocks of dyads that live together versus those that live apart, in equal numbers to the two study conditions. Allocation will be concealed, with the RA randomizing participants to study arms using a computer-generated randomization series.

9.5 Single-Blinding Procedures

Study analysts and the study PIs and Co-Is will be blind to study assignment of the dyads. The Project Manager, RA and DEC will by necessity be aware of which participants are assigned to the intervention group. To ensure study analysts are blinded to study assignment, the DEC and IVR databases tracking intervention participation will be kept separate from study assessment databases until main outcome analyses have been completed.

9.6 Subject Withdrawal

9.6.1 Reasons for Withdrawal

Subjects may choose to stop participating for any reason at any time.

If a subject becomes newly ineligible during the study, according to eligibility/ineligibility criteria listed in section 5 above (i.e. develops a terminal illness), they will be notified that their eligibility has changed and will be withdrawn from the study by study staff. Based on signed HIPAA, study data up to that point will be retained for Intention to Treat analysis purposes.

9.6.2 Handling of Subject Withdrawals or Subject Discontinuation of Study Intervention

Subjects may choose to stop participating at any time. We will record the date and reason for withdrawal. For patient participants who wish to stop participating in the intervention or in primary data collection (surveys, study-related lab measurements), we will ask if they are willing to remain in the study for purposes of secondary data collection only (data available in the subjects' medical record, collected per study protocol).

If a subject stops responding to study contacts (DEC calls, assessment calls, IVR contacts), study staff will contact them directly to determine whether they would like to continue with the study. In the absence of any response, participants will be considered enrolled in the study. Patients who are unable to be reached for their 12 month follow up survey will be considered 'lost to follow up' for analysis purposes.

If a Care Partner becomes ineligible, or elects to withdraw from the study, patient-partners assigned to the intervention group will be offered the option to continue with patient-focused IVR calls and DEC contacts for the remaining duration of the study period. The patient will be instructed to ignore any IVR references to the Care Partner. All patient participants who remain enrolled but whose Care Partners drop out will be contacted for the 12 month assessment and be included in the intervention group in intent-to-treat analyses.

9.7 Procedures to Maintain Study Enrollment

Participants in the intervention group will receive contact at least every two weeks in the form of IVR calls (for patients) and summaries of the IVR calls (for Care Partners). We anticipate that this frequent contact and provision of helpful information, in addition to other intervention contacts with incentives provided at 6 months and 12 months, will encourage sustained enrollment.

Participants in the control group will receive contact at baseline, 6 months, and 12 months, and at each time point, they will receive incentives for completion of assessments. At these points of contact, participants will be thanked for their contributions and reminded of the value of their contributions.

Additionally, at enrollment, patients will be offered a tote bag with the study logo, and with the mailed reminder of the 6 month survey, participants will receive a magnet with the study logo that is a mini dry-erase board and a pen. We anticipate that these actions will maintain study enrollment.

10. Intervention

10.1 Preparation

10.1.1 Training of Dyad Engagement Coach

The Dyad Engagement Coach will be trained in all study protocols, and ability to extract clinical data for initial visit and post-visit summaries and ability to follow DEC contact scripts will be evaluated before the start of the trial.

10.2 Primary Care Staff Orientation

Prior to intervention start, primary care staff (primary care providers, nurses, and clerks) will be oriented to the intervention during regularly scheduled provider & staff meetings. At these meetings we will:

- Describe the study process for screening patients and Care Partners
- Share the protocol and fax template for contacting primary care teams if an urgent clinical issue is detected
- Share clinically related forms used in the intervention: the form completed during the initial session, the clinical visits planning worksheet, and the template for the Visit Summaries.
- Ask providers to notify study staff if they do not wish for their patients to participate in general
- Ask primary care staff to report to study staff any Care Partners that they feel are interfering with patient well-being or clinical care.

At another provider and staff meeting, staff will be given a brief training and reference materials on positive and productive communication with patients' family members.

10.3 Initial Session with the Dyad Engagement Coach (DEC)

This session will take place within 2 weeks of enrollment whenever possible. The patient will meet in-person with the DEC at [REDACTED]AHS. The Care Partner will either be present in person or on speakerphone

during the visit. Care Partners who participate by phone will be pre-mailed printed materials and guided to the intervention website during the session if possible. This visit is anticipated to take about 45 minutes.

10.3.1 Agenda of the Initial Session

- Explain the role of the coach
- Discuss the Care Partner's role /ice breaker
- Discuss patient's diabetes-related health information
- Provide program informational resources: website and binder
- Patient Engagement
  - Communicating with Health Care Providers
  - Action Planning
- Effective Supporter Techniques
- Navigating VA Resources
  - Explain what the PACT team is and how to reach them
  - Point out list of Ann Arbor VA Diabetes-Related Programs
- The CO-IMPACT Program (what's next)
  - Describe automated calls and determine call day and time
  - Weekly talks / action planning
  - Before and after primary care appointments
- Wrap-up

A written summary of this session will be given to patients and Care Partners and an initial session medical record note placed in the patients' medical record for their PACT teamlet to view and co-sign.

10.3.2 Initial DEC Session Materials

After the initial session, patients and Care Partners will be able to review the session's educational content, strategies, and talking points via a study website and a binder of printed material. Both Care Partner and patient will be given login information to access the website, and the patient will receive a binder with same content. If the Care Partner prefers to receive a personal copy of the binder, the Care Partner will also receive a binder.

These materials, both on the website and in the binder, will include:

- General information about diabetes management (similar to that which patients receive in VA diabetes and PACT education)
- Tips for patient-clinician communication and visit preparation (based on VA brochures such as "TEAM UP For Your Care")
- Referred to VA website for care partners.
- Care Partners program guidelines
- General ways Care Partners/family can facilitate diabetes management in day-to-day life and limits of Care Partner roles
- How Care Partners can provide autonomy-supportive encouragement
- Importance of patient/Care Partner engagement between visits and active participation in encounter
- Tips on best Care Partner communication with patient's doctors/nurses
- Steps in effective action planning and setting SMART goals
- Info on PACT teamlet members and their roles and how to access PACT services (based on standard PACT orientation brochures)
- Info on navigating the [REDACTED]VA (e.g. information on obtaining medications and making appointments)
- Info on VA diabetes programs available and on MyHealth*e*Vet (standard brochures available to patients at [REDACTED]VA)
- Steps to take if patient receives care in a non-VA facility
- Logs
  - Glucose and Blood Pressure Home Testing Log

- Medication Log - to record medication regimen, medications taken
- Events Log – to record illnesses, ED visits, hospitalizations
  - Care Partner-Patient Talk Log

- Worksheets
  - Visit planning worksheets for primary care appointments
    - Questions for provider
    - Information to bring from home
    - Information to bring from outside providers
  - SMART Goal/Action Planning Worksheet
    - Current goal(s) and progress towards/barriers against them

10.4 IVR Component

Patients will receive automated IVR assessment calls once every two weeks. The goal of these calls will be to prompt continued progress towards diabetes goals and Care Partner involvement between PACT visits.

Patient calls will consist of statements and queries recorded in a human voice, to which they can respond by selecting a number on their touch-tone pad. During each call, patients will be asked whether they are experiencing any diabetes management concerns for which taking action within the next weeks would be prudent. These topics include more high sugars, low sugars, bothersome medication side effects, or running short on medication supply. After a patient completes an IVR call, the Care Partner will receive an automated summary, via structured email or mailed letter (if Care Partner does not use email), with any identified action issues and whether or not the patient plans to address the issue over the next two weeks. Supporter messages following each completed patient call will include reminders to discuss diabetes care with the patient, using the talking-points and guidelines provided at the initial session, and more detailed information on those issues the patient identified as potentially requiring action. The patient's PACT nurse care manager will receive an automated fax alert when patients identify the following clinically urgent issues:

- a blood sugar level below 70 more than twice in the past two weeks
- a blood sugar level below 80 more than twice in the past two weeks and symptoms of low blood sugar such as sweating or trembling, plus feeling irritable, confused or weak.
- a fasting blood sugar level above 300 more than twice in the past two weeks
- a systolic blood pressure less than 90 more than once in the last two weeks
- a systolic blood pressure less than 100 more than once in the last two weeks and symptoms of low blood pressure such as feeling dizzy, confused or weak
- a systolic blood pressure over 170 at least once in the last two weeks

10.5 Patient-Care Partner Regular Discussions

Participant guidelines specify several parameters for Care Partner-patient discussions about diabetes care. These are to:

a) talk approximately once per week about diabetes management for at least 10 minutes per occasion to review recent assessments and trends
b) use supportive comments and avoid criticism
c) collaboratively approach problem solving
d) review progress and barriers to achieving past diabetes management plans
e) discuss any recent or upcoming primary care appointments.
f) the Care Partner will encourage the patient to contact his or her clinical team in appropriate situations.

10.6 Visit Preparation

After the initial coaching session, the study team will monitor VISTA appointment files for enrolled patients to identify upcoming PACT visits. A qualifying visit will be an in-person visit to a PACT PCP, nurse, or clinical

pharmacist. Approximately one week before each qualifying visit, the Dyad Engagement Coach (DEC) will conduct a visit planning session with the patient via telephone. During that call, the DEC will use a visit planning worksheet (included in the binder) to help the patient identify any diabetes risk-related questions or concerns they would like to address during their visit, as well as diabetes-related information, such as home monitoring logs, they will bring to the visit. The DEC will help patients role-play asking the one or two questions most important to them. Patients will be free to add non-diabetes related questions or information to their visit planning worksheet, but these will not be specifically elicited by the DEC. If the enrolled Care Partner is present with the patient at the time of the call and can join using speakerphone or another phone on the same line, they will be invited to participate in the call. The DEC will document this call in CPRS and add the teamlet LPN as co-signer. Thus, the teamlet LPN will not need to conduct their usual visit reminder phone call for this patient.

Care Partners will receive an email notifying them that their patient-partner has an upcoming primary care visit. It will contain a website link to the visit planning worksheet, and the message will encourage Care Partners to use the worksheet with their patient-partner and add questions and concerns for the patient's visit. Care Partners who do not have access to email will be mailed a letter with similar content and a referral to the same worksheet in their binder.

10.7 After Visit Summary

We will be using a slightly modified version of the AviTracks medication reconciliation program currently used in clinical care in [REDACTED]HS that will generate and print a patient-friendly Visit Summary from Vista/CPRS. Within three days of a completed, qualifying PACT visit, the Dyad Engagement Coach will mail to the patient their visit summary. Three business days following the mailing (to allow time for the Visit Summary to be delivered to the patient), the Dyad Engagement Coach will post the Visit Summary to the study's secure website. The posting will trigger an immediate email to the Care Partner that notifies them that the summary is available on the website. If the Care Partner does not use email, the Visit Summary will be mailed to them.

10.8 Fidelity

A predetermined sequence (the first 10, then 10% of the remaining) of visit planning calls will be recorded for review by study investigators to assess intervention fidelity, quality of interactions, and to provide feedback to coaches. DEC-created documents will also be reviewed. Patient appointment and IVR call records will be monitored regularly by study staff for level of missed contact opportunities.

Audiorecording of these calls is not required for the intervention, and participants who decline to be recorded will still be able to fully participate in calls. These files will be not be labeled with participant IDs and will be moved to the HSR&D data repository only accessible by the HSR&D data manager and will be destroyed when the new records control schedule is published.

11. Control Condition

Patients assigned to the control condition will receive usual PACT care for diabetes at [REDACTED]HS facilities that are at an advanced stage of PACT implementation. PACT care for diabetes is expected to follow VA/DoD diabetes management guidelines. These patients are then eligible for PACT services at the teamlet's discretion.

Study staff will also provide control group patients with access to the study website pages that contain educational information on general diabetes management; patients will receive the same educational information in hard copy, and Care Partners may also receive the same binder if they prefer. Study staff will ensure that patients have home glucometers and blood pressure monitors, if desired by the patient. Patients in the control condition will not be precluded from involving Care Partners in medical visits or VA health programs.

12. Data Collection Procedures

12.1 Patient

Baseline:

Immediately after in-person informed consent is obtained by study staff, the study staff will administer an assessment consisting of self-reported survey items, blood pressure measurement, and a lab order so that patients provide venous samples for Hemoglobin A1c (HbA1c) tests and non-fasting Lipid Panels through the VA lab. The RA will read each survey question and record the patient's answer on the paper survey. The patient will complete the survey in a private room while their Care Partner, if present, will be asked to wait outside of the room. Patients will be informed that they can ask questions of the RA if they have trouble with any part of the survey. If patients have had an HbA1c performed at a [REDACTED]HS facility as part of routine care 4 or fewer weeks prior, or if they had a lipid panel performed 4 or fewer weeks before, that test result will be used for the study assessment and an additional study-associated venipuncture will not be performed. Prior to the enrollment meeting, RAs will go into CPRS to determine if the labs were conducted within the four week window. Patients do not need to pay any fees for the bloodwork. We anticipate this assessment will take about 45 minutes to complete, exclusive of time required to complete bloodwork at the VA Laboratory. Patient participants will receive a $50 gift certificate upon completing the baseline assessment.

6 months:

At 6 months after baseline assessment, the patient will complete a short survey of self-reported items including the PAM-13, how their Care Partner is involved in their health care, and diabetes self-management adherence. Study staff will conduct a medical record review of patient participants to obtain HbA1c, lipids, blood pressure, and smoking status at this time point. The patient will complete the survey by one of two options:

1. a hard copy of the survey is mailed to participant, completed, and mailed back in a postage-paid envelope
2. the survey is administered via telephone interview by study staff

The patient will asked their preferred option at the end of the baseline assessment. If the patient does not complete the mailed version within two weeks, study staff will call to remind the patient and give them the option of scheduling a telephone interview instead. We anticipate this assessment will take about 15 minutes to complete. Patient participants will receive a $15 gift certificate upon completing the 6-month assessment.

12 Months:

At 12 months after the baseline assessment, study staff will measure the patient's blood pressure and administer a survey of self-reported items using the same methods described above for the baseline assessment. In addition, study staff will ask a few open-ended questions on the participant's experience of and impressions of the intervention, or control materials. HbA1c tests and non-fasting Lipid Panels will be obtained through the VA lab. If patients have had an HbA1c performed at a [REDACTED]HS facility as part of routine care 2 weeks before, or if they had a lipid panel performed within 4 weeks before the assessment date, that test result will be used for the study assessment and an additional study-associated venipuncture will not be performed. We anticipate this assessment will take about 45 minutes to complete, exclusive of time required to complete bloodwork at the VA Laboratory. Patient participants will receive a $50 gift certificate upon completing the 12-month follow-up assessment.

Patient pharmacy (medication fills) and encounter data will be collected from the following time periods:

- 12 months prior to baseline for calculating baseline measures
- from baseline to 24-month post-baseline to calculate follow-up measures.

12.2 Care Partner

Baseline:

A baseline assessment consisting of self-reported survey items will be conducted over the phone by study staff after Care Partner consent. Assessment should take a little less than 45 minutes to complete. Care Partner participants will receive a $20 gift card to following completion of the baseline assessment.

6 months:

An assessment consisting of self-reported survey items for Care Partner participants will be conducted. The Care Partner will complete the survey by one of two options:

1. a hard copy of the survey is mailed to participant, completed, and mailed back in a postage-paid envelope
2. the survey is administered via telephone interview by study staff

The participant will be asked their preferred option at the end of the baseline assessment. If the patient does not complete the mailed version within two weeks, study staff will call to remind the patient and give them the option of scheduling a telephone interview instead. We anticipate this assessment will take about 15 minutes to complete. Care Partner participants will receive a $15 gift certificate upon completing the 6-month assessment.

12 months:

At 12 months after the baseline assessment, the Care Partner will complete an assessment consisting of self-reported survey items that is very similar to the baseline assessment. In addition, study staff will ask a few open-ended questions on the participant's experience of and impressions of the intervention, or control materials. Study staff will administer the full assessment over the phone. We anticipate this assessment will take about 45 minutes to complete. Care Partner participants will receive a $20 gift certificate upon completing the 12-month follow-up assessment.

12.3 Dyads

Sixteen Dyads will be purposively sampled for more extensive semi-structured qualitative interviews at 12-15 months after baseline (see section 13.6 below)

12.4 Intervention Processes

The CO-IMPACT automated IVR system will capture patient participants' responses to questions about symptoms, medication adherence, and home glucose and blood pressure readings. The system will automatically track dates and times of all assessment attempts and whether they are completed. The CO-IMPACT website will automatically collect data on access to various parts of the website. Data from coach initial session and visit planning call session logs will be also be captured (e.g., length of session, attempts made to schedule session, etc.).

12.5 DEC Experiences and Feedback

After all 240 patients have completed the study period, study staff will conduct a semi-structured interview with the DECs.

13. Study Measures

13.1 Patient Outcomes

Health Behaviors and Behavioral Determinants: The study's main outcome measure will be the Patient Activation Measure-13 (PAM-13). The PAM-13 has been widely used to measure patient activation in longitudinal studies, and in clinical trials as a primary outcome measure, and scores have been responsive to intervention. The PAM-13 is reliable (Cronbach alpha 0.87), and improvement in PAM-13 scores has been linked to improvement in self-management behavior. A 4-6 point change in the PAM is considered clinically significant. We will also measure patient activation in medical visits with the Perceived Efficacy in Patient-

Physician Interactions (PEPPI-5). Items include “I am confident in my ability…to get a doctor to answer all of my questions” and “to get a doctor to take my chief health concern seriously”. The PEPPI-5 has been validated against other self-efficacy and patient satisfaction scales, and is reliable (Cronbach alpha 0.92).

Health Risks: To address the effect of CO-IMPACT on patient health risks, our main measure will be the 5-year UKPDS Risk Engine. This score estimates the risk of a coronary heart disease (CHD) event (fatal or non-fatal MI, or sudden death) specifically among people with diabetes. The score components include factors we hypothesize could be improved by the intervention, including HbA1C, systolic blood pressure (SBP), total cholesterol/HDL cholesterol ratio, and smoking status. The score also includes age, sex, race/ethnicity, and length of time since diabetes diagnosis. The UKPDS Risk Engine has been validated in multiple populations. Using a cardiac risk score to measure risk factor changes offers the advantage of quantifying the cumulative impact of changes in multiple risk factors, and translating changes in physiologic parameters to a risk estimate that is meaningful to patients and policy makers. For similar reasons, cardiac risk scores, including the UKPDS Risk Engine and the Framingham Risk Score, have been successfully used as outcomes in multiple clinical trials.[43–47] The UKPDS Risk Engine has been validated in multiple populations.[48] A 1-2% change in risk is considered clinically significant at a population level. In preparation for this study, we measured UKPDS risk among 434 [REDACTED]HS patients randomly selected from a high-risk diabetes registry with similar inclusion criteria to this study. Mean 5-year UKPDS risk in this population was 18%, SD 12%. We then simulated the changes in risk that would result from changes in individual score components. We found that an average 0.5% decrease in HbA1c over 1 year led to an average 1.3% decrease in UKPDS risk over that of the same population with no change in HbA1c. Similarly, a 10mmHg decrease in SBP led to a 1.3% risk decrease, and a 30% decrease in total cholesterol among those with total cholesterol >160mg/dL (to simulate new adherence to a statin) led to a 3% risk decrease.

HbA1C, lipid levels, blood pressure, and smoking status will be analyzed independently as secondary health outcomes. We will measure, via survey, patients' frequency of hypoglycemia, and diabetes distress. Patients' use of VA urgent care will be extracted from the EMR for the period 12 months prior to intervention start and during the 12 month study period, supplemented by patient report of non-VA urgent care.

Table 2: Details on Selected Patient Measures

Construct	Source	Instrument(s)	BL	6M	12M
**Health Behaviors and Determinants**					
Activation	Survey	PAM-13	X	X	X
Activation in Health Encounters	Survey	PEPPI-5	X	X	X
Diabetes Self-Efficacy	Survey	Stanford Chronic Disease Self-Efficacy Scale[41]	X	X	X
Diabetes Self-Management Behavior (self-monitoring, healthy eating, physical activity)	Survey	Summary of Diabetes Self-Care Activities[49]	X	X	X
Diabetes Medication Adherence	EMR x12 months	Cumulative Medication Gaps <20%[50]	X		X
**Health Outcomes**					
5-Year Cardiac Event Risk		UKPDS 5 year cardiac risk score	X		X
Glycemic Control	Venous Sample	HbA1C	X		X
Blood Pressure	Direct measure	Systolic Blood Pressure, Mean Arterial Pressure	X		X
Non-fasting Lipid Levels	Venous Sample	Total Cholesterol/HDL	X		X
Smoking Status	Survey		X	X	X
Diabetes Distress	Survey	Problem Areas in Diabetes Scale[51]	X		X
**Patient-Supporter Relationship and Support Quality**					
Patient-Supporter Relationship Quality	Survey	Relationship Rating Form – Respect Subscale[52]	X		X
Patient Satisfaction with Diabetes Social Support	Survey	Diabetes Care Profile – Support Subscale[53]	X	X	X

Supporter use of Autonomy Supportive Communication	Survey	Important Other Climate Questionnaire[54]	X		X
Patient-Partner Closeness	Survey	Subjective Closeness Index[55]	X		X
**Patient-Provider Relationship**					
Patient-provider trust	Survey	Primary Care Assessment Survey-Trust Subscale[56]	X		X
Patient-provider shared decision making	Survey	Provider Participatory Decision-Making Style[57]	X		X
**Potential Moderators**					
Time with Diabetes	Survey		X		
Patient Comorbidities	EMR x12 months	Charlson Comorbidity Index[58]	X		
Health Literacy	Survey	Brief Health Literacy Screen[59]	X		
Current PTSD symptoms	Survey	Primary Care-PTSD Screen for DSM5[60]	X		
Depression and Anxiety	Survey	Patient Health Questionnaire-4[61,62]	X		

Patient-Supporter Relationship and Support Quality: We will measure overall relationship quality for both patients and supporters (see Tables 2 and 3). Patient satisfaction with overall quality of diabetes support received and supporter use of autonomy-supportive communication will be assessed via patient survey. Supporters and patients will be surveyed about concerns about health privacy breaches. The Subjective Closeness Index (SCI)[46,47] will be used to assess patient and Care Partners perceived closeness to each other. We are interested in examining the effect of the intervention on patient-Care Partner closeness. The SCI is comprised of two items and has demonstrated convergent validity with other measures of inter-personal closeness.[47] Further, prior research indicates that SCI measures interpersonal closeness irrespective of gender and relationship type (e.g., romantic relationship vs. friendships).[46]

Patient-Provider Relationship and *Patient Satisfaction with VA Health Care:* We will measure patient-provider communication, trust, and level of shared decision-making via patient survey (Table 2). We will measure patient satisfaction with PACT care using a question from the VA Consumer Assessment of Healthcare Providers and Systems (CAHPS)-PCMH, and patient satisfaction with PACT engagement of Care Partners using questions developed in our pilot.

13.2 Care Partner Outcomes

We will measure changes in Care Partner roles (e.g. help track patient medication use at home) via surveys at baseline, 6 and 12 months. Care Partners' self-efficacy for helping patients with diabetes, supporter distress about the patient's diabetes, and supporter distress about patient hypoglycemia, will be measured with adaptations from similar validated patient measures. These supporter-adapted measures were used in our pilot intervention assessment. In this study, we will calculate psychometric properties of these measures, and associations with validated supporter measures, among our 240 Care Partners. Caregiving burden will be assessed with the reliable and validated Multidimensional Caregiver Strain Index. See Table 3.

13.3 Patient and Supporter Moderators of Effect

Theoretical patient moderators of intervention effects include (also see Table 2): sociodemographics (sex, age, education), baseline diabetes medication regimen, distance from VA site, comorbidities, health literacy level, and co-morbid depressive symptoms. Additional moderators include: whether the patient and supporter live together, whether the supporter has diabetes, supporter depressive symptoms, baseline patient-supporter and patient-physician relationship quality, and whether Care Partners attend patient visits in person.

The Primary Care PTSD screen for DSM5 (PC-PTSD-5)[62] will be used to identify Veterans with probable PTSD at baseline. PTSD is relatively common (estimated prevalence: 11.5-32.6%) among VA primary care patients.[65] Existing research suggests that adults with PTSD have lower levels of physical activity,[66] poorer dietary behaviors,[67,68] and are at elevated risk for weight gain and cardiometabolic conditions[69,70]. Some evidence suggests that Veterans with PTSD do not benefit from health behavior programs (e.g., VA MOVE!) to the same extent as Veteran without PTSD.[71,72] Accordingly, we would like to compare the interventions effects of the current study among Veterans with and without probable PTSD at baseline. The PC-PTSD-5 is comprised of six "yes" or "no" items and was designed to screen for PTSD within the primary care patient populations and has been validated among a large sample of Veteran primary care patients.[60] The PC-PTSD-5 has a very high level of diagnostic accuracy for identifying primary care patients PTSD.[60] Scores ≥3 have a high level of sensitivity and specificity (0.85) for identifying primary care patients PTSD.[60] VA/DoD PTSD Management Guidelines recommend screening all VA primary care patients, using the PC-PTSD-5 or other brief instrument, on an annual basis.[72] Consequently, study participants (i.e., VA primary care patients) are likely familiar with the PC-PTSD-5. We are interested in assessing participants' current PTSD status which, unfortunately, cannot be ascertained using prior PC-PTSD-5 screen results with participants' electronic medical records. Results from the initial validation study indicate that Veterans feel comfortable completing the PC-PTSD-5 and find the measures easy to understand.[60]

Table 3: Details on Selected Supporter Measures

Construct	Source	Instrument(s)	BL	6 M	12M
**Behaviors and Determinants**					
Supporter Self-Efficacy for Helping Patient With DM Care	Survey	Adapted Stanford Chronic Disease Self-Efficacy Scale [41]	X	X	X
**Health and Relationship Outcomes**					
Caregiver Burden	Survey	Caregiver Strain Index[63]	X		X
Supporter Distress About Patient's Diabetes	Survey	Adapted Problem Areas in Diabetes Scale[51]<br>Adapted Fear of Hypoglycemia – Worry Subscale[64]	X<br>X	X<br>X	X<br>X
Patient-Supporter Relationship Quality	Survey	Relationship Rating Form – Respect Subscale[52]	X		X
Patient-Partner Closeness	Survey	Subjective Closeness Index[55]	X		X
**Potential Moderators**					
Depression and Anxiety	Survey	Patient Health Questionnaire-4 [61,62]	X		

The PHQ-4[62]which is an ultra-brief screen for depression and anxiety. The PHQ-4 is comprised of a two-item depression scree (i.e., PHQ-2) and a two-item anxiety screen (i.e., GAD-2). PHQ-4 total scores can be used to measure of general psychological distress.[61] Scores greater ≥6 screen positive for significant psychological distress and will be referred to Primary Care Mental Health.

13.4 Provider Behavior and Impact on PACT Teamlet

A theoretical mediator of intervention effect, medication intensification, will be measured via EMR similarly to methods used by Dr. Kerr previously. [73] In interviews with PACT clinical staff, we will assess whether clinicians perceived changes in: effectiveness or efficiency of patient-clinician communication; any unintended consequences on privacy or clinician comfort; clinician awareness of supporter roles at home; and relationships with patient supporters.

13.5 Intervention and Control Processes

We will record the frequency of each type of DEC contact with intervention-assigned participants, and time spent in preparation and execution of each contact. We will automatically capture outcomes of all IVR call attempts, and number of page visits and downloads from the study website. For participants in both arms we will capture via the EMR the number of completed PACT PCP, nurse, and clinical pharmacist encounters, occurring in-person or by phone. We will ask participants via survey whether they received after-visit summaries after PACT in-person visits. We will tally consults entered by PACT teamlets to diabetes risk related programs, and patient (via EMR) and supporter (via survey) rate of attendance. Finally, we will ask all patients and supporters about the frequency of general discussions about diabetes, clinical visit preparation discussions, and post-visit debriefing.

13.6 Facilitators and Barriers to Future Implementation

We will ask eligible patients and Care Partners who decline participation to provide consent for a brief survey including reasons for not enrolling. We will conduct semi-structured interviews of selected participants and clinicians to evaluate facilitators and barriers to intervention implementation. Eight dyads will be purposely sampled from those with high vs. low engagement in the intervention (as measured by rate of pre-visit and IVR call completions), and eight from those with high vs. low level of improvement in cardiac risk score.

14. Statistical Analysis Plan (Final)

14.1 Overall Approach

We will follow international guidelines for analysis and reporting of clinical trials. We will examine baseline data for prognostically important differences across the two study groups, such as patients' age, race, comorbidities, and baseline use of services. Although we do not anticipate any imbalances, any baseline differences between experimental arms will be included as covariates in analyses comparing outcomes. Missing data will be imputed for non-outcome measures, using multiple imputation methods. If we find baseline variables to be associated with the loss to follow-up, we will include those baseline variables as covariates in models evaluating the intervention effect.

14.2 Unit of Analysis and Sample Size Calculation

Our main aims are to evaluate effects at the patient level. Our sample size calculations are based on our primary outcome of patient activation, measured by the PAM-13. Assuming that PAM-13 was highly correlated between baseline and 1 year ($r = .70$), we calculated our sample size to provide a minimum of 80% power to detect a between group difference in PAM-13 change of 4.0, with a standard deviation of change of 13, and a two-tailed alpha of 0.05. To achieve 80% power, a minimum of 102 patients is needed in each group, for a total sample size of 204. To allow for 15% attrition, we will enroll 120 patients in each group, for a total of 240 patients.

This sample size will also be sufficient for detecting clinically-significant differences in the secondary outcome of change in 5-year UKPDS cardiac risk score. Assuming the underlying correlation between UKPDS at baseline and 1 year later is .90, our sample size of 102 per group will provide more than 80% power for detecting between group differences in predicted cardiac risk of 2.0% (SD=12), which is considered clinically significant on a population level.. Based on estimates from our pilot study and other observational research, a 2.0% or greater change in intervention patients is achievable in the CO-IMPACT intervention.

14.3 Primary and Secondary Outcomes (Aims 1 and 2)

Preliminary analyses. We will construct trajectory plots of all three PAM-13 measurements to understand general trends over the study period. Although baseline differences between groups are not expected due to randomization, this will be validated for key sociodemographic and health characteristics of participants via a series of independent samples *t*-tests and chi-square analyses. Missing data will be examined for randomness. We expect that key variables such as the scaled PAM-13 will be normally distributed, but highly skewed measures will be transformed if needed.

PAM-13 will be administered by a research assistant in person or over the phone at baseline and 12-months. In addition, 6 months following the start of the study, PAM-13 will be given to all participants via internet or mail survey. The raw PAM-13 score will be transformed according to a formula established by the creators of the PAM and its licensee Insignia Health. Transformed PAM-13 scores range from 0-100 where higher values indicate greater degree of activation. Due to the different modes of administration, there is the potential for notable differences in reliability and disproportionate missing data between the 6 month collection and the other collection time points. Consequently, PAM-13 measured at 6 months will be used only in supplemental analyses to assess the possible curvilinear trajectory of patient activation as well as the dependence of this trajectory on treatment group.

We will first evaluate bivariate associations between the study group condition (by intention to treat principles) and outcomes using two-sided, two-sample t–tests for continuous measures and Pearson's chi-square tests for categorical measures. We expect that key outcomes such as the PAM-13 will be normally distributed, but measures will be transformed if needed. We will then use multivariable regression models, taking into account baseline score of the outcome, to identify main effects.

Main analyses: All main analyses will be conducted using Intention to Treat principles. Main analyses for both Aims 1 and 2 will be performed using hierarchical linear models (HLM) with scaled PAM scores (at baseline and 1 year) and UKPDS scores (baseline and 1 year) respectively as the outcomes. HLM, or mixed models, incorporates both fixed and random effects. A fixed effect indicates that only specific levels of a variable are studied and inference is made only to the measured levels, consequently no variance parameter is estimated for these effects. In this study fixed effects include treatment group, time, and whether the Care Partner and participant lived together. All are dichotomous predictors: time is coded as 0 = baseline and 1 = the 1 year point; treatment group is indicated by 0 = control (PACT only) and 1 = treatment (PACT + CO-IMPACT); living situation is 0 = living apart and 1 = living together. Random effects on the other hand imply that not all levels of a variable are represented but indeed exist. In this study where a longitudinal model is used, a random variable representing differences in mean outcome scores is represented by a random component (u_{0j}) that captures information about the outcome not explained by the fixed effects (time, treatment, etc.).

Basic model. The fundamental model for Aims 1 and 2 is represented by the respective outcome at time point (*i*) for person (*j*) as a function of time, treatment group, the interaction between them, and a person-level effect indicating whether the Care Partner lives with the participant (1) or lives apart (0):

eq. 1:

$$score_{ij} = \gamma_{00} + \gamma_{10}(time)_{ij} + \gamma_{20}(treatment)_j + \gamma_{11}\left[(time)_{ij}(treatment)_j\right] + \gamma_{30}(lives\ with\ CP)_j + u_{0j} + r_{ij}$$

where:

$$u_{oj} \sim N(0, \tau_{00})$$
$$r_{ij} \sim N(0, \sigma^2)$$

Aim-Specific Models

Aim 1 primary analysis. In this model the fixed effects are as follows: $\hat{\gamma}_{00}$ is the mean of PAM-13 scores at baseline for the control group who were not living with their Care Partner; the significance test assesses if this mean is 0 (*cf.* eq. 1). The change in PAM-13 scores across time is described via the $\hat{\gamma}_{10}$ and $\hat{\gamma}_{11}$ parameter

estimates; if there is a significant interaction of time and treatment, the change in PAM scores differs *depending on* treatment group. The change in PAM score for the control group is revealed in the $\hat{\gamma}_{10}$estimate; how much PAM differs across time for the treatment group will be captured in the $\hat{\gamma}_{11}$estimate. Similarly, the treatment effect is modified by the interaction term -- $\hat{\gamma}_{20}$ captures the mean difference in PAM scores for the groups at baseline, while the mean difference at the final observation period must be interpreted through both $\hat{\gamma}_{20}$ and $\hat{\gamma}_{11}$. In other words, the differential change over time based on treatment group is indicated by the $\hat{\gamma}_{11}$ effect; here, a significant test reveals that the change in PAM over time is different depending on treatment group. This is the primary effect of interest for Aim 1. Further, participants will be randomly assigned to treatment condition within the stratum of whether the Care Partner lives with the patient or lives apart; this effect is represented by $\hat{\gamma}_{30}$ which tests for the mean difference in PAM scores between these groups after adjusting scores for treatment, time, and the time × treatment interaction.

The random component includes u_{0j} which represents the how much a person's average PAM score differs from the rest of the sample after controlling for the other effects in the model. Stated another way, the variance component of this effect ($\hat{\tau}_{00}$) captures the average deviation between participants' PAM scores and the baseline mean for the control group after controlling for treatment, time, and living status effects. If this is different from 0 then it supports the idea that something is driving PAM scores beyond time, treatment, and living situation. As more fixed effects are included in the model this parameter estimate approaches 0. If $\hat{\tau}_{00}$ is not statistically different from 0, the result is a fixed (not random or mixed) effects model and the remaining variability in PAM scores is from truly random, idiosyncratic sources (r_{ij}). This other random effect (r_{ij}) captures the variation in PAM scores at a particular time point for a particular person that is not explained by the other effects.

Aim 1 auxiliary analyses. As a supplementary analysis, we will analyze differences in PAM score by interacting with four baseline PAM strata, as a priori defined by the PAM scale developer.

As another supplemental analysis, the curvilinear trajectory of PAM-13 over time will be tested with growth curve modeling using the PAM-13 score at 6 months in addition to PAM-13 at baseline and 1-year. Further, random effects models will be used to examine differences in this trajectory based on treatment group can be examined. This is represented as:

$$PAM_{ij} = \gamma_{00} + \gamma_{10}(time)_{ij} + \gamma_{20}(time^2)_{ij} + \gamma_{30}(treatment)_j + \gamma_{11}\left[(time)_{ij}(treatment)_j\right] + \gamma_{40}(lives\ with\ CP)_j + u_{0j} + r_{ij}$$

Aim 2 primary analysis. Consistent with the statistical model for Aim 1, the fixed effects for Aim 2 is represented by 5-year UKPDS score at time point (*i*) for person (*j*) as a function of time, treatment group, the interaction between them, and living situation of the Care Partner. Specifically, $\hat{\gamma}_{00}$ is the mean cardiac risk at baseline for the control group who live apart from their care partner. The overall change in risk across time for all people is seen in $\hat{\gamma}_{10}$ – the test of this parameter asks whether risk changes across time after adjustment for treatment influences and CP living status. The mean difference in risk scores between treatment and control groups, regardless of time, is measured by $\hat{\gamma}_{20}$; namely, this is how much the average risk score for treatment group deviates from the control group so that a significant $\hat{\gamma}_{20}$ indicates a significant mean difference in PAM scores as a function of treatment group. The differential change over time based on treatment group is indicated by the $\hat{\gamma}_{11}$ effect; here, a significant test reveals that the change over time in cardiac risk is different depending on treatment group. Mean difference in UKPDS score between those who live with their Care Partners and those who don't after adjusting scores for treatment, time, and the time × treatment interaction is measured via $\hat{\gamma}_{30}$.

The random component includes u_{0j} which represents the how much a person's average risk score differs from the rest of the sample after controlling for the other effects in the model; the variance component of this effect ($\hat{\tau}_{00}$) captures the average deviation between participants' risk scores and the grand mean. If this is different from 0 then it supports the idea that something is driving risk scores beyond time, treatment group, and living situation.

Aim 2 auxiliary analyses. The same fixed effects of the fundamental model will be retained, but the outcomes will be selected individual components of the UKPDS analyzed independently as secondary health outcomes: HbA1c, systolic blood pressure (SBP), total cholesterol/HDL cholesterol ratio, and smoking status.

14.4 Mediators and Moderators of Intervention Effect (Aim 3)

We will use multivariable regression models to examine potential mediators and moderators of intervention effects. We will introduce potential mediators to models linking intervention condition to outcomes, examining changes in the magnitude of the relationship between the intervention and the outcomes before and after the covariates are introduced. We will also use the Preacher and Hayes bootstrapping method to examine potential mediators to determine whether the mediation effect is significant. This is a non-parametric method that can be used when the outcome violates assumptions of normality. Potential mediators are specified in our theoretical model (Figure 1), and include an index of Care Partner engagement in the intervention, composed of measures of supporter participation in intervention sessions, and reported use of pre-visit preparation and debriefing tools. Analyses of potential moderators (as in section 13.3) will use standard approaches to evaluate interactions between these covariates and the intervention, which will include plotting regression lines for high and low values of the moderator variable using Stata routines[74] Independent variables and moderators will be centered before testing interactions, so that multicollinearity between first order and higher-order terms will be minimized.

14.5 Qualitative Analysis

We will conduct a thematic analysis of interview transcripts using the "Editing Analysis Style,"[75] which contains both deductive and inductive elements. Following this approach, Drs. Rosland and Heisler will independently read interview transcripts, break down responses into individual segments that express a single idea or theme (e.g., ways participants found pre-visit calls useful or not useful) and label these phrases with appropriate codes. An iterative process will be used to compare results until agreement is reached on the codes and their definitions, after which we will apply the codes to the remaining transcripts. Emerging themes will be compared across patients and compared to patterns in survey responses.

14.6 Process Evaluation

We will use the RE-AIM framework[76] to guide this analysis. To analyze the potential reach of the intervention, we will calculate the proportion of patients with diabetes who meet inclusion criteria and compare characteristics of eligible and non-eligible dyads. Effectiveness will be measured via our main outcomes and differences in outcomes among key patient groups. We will evaluate adoption by examining the characteristics of patients and supporters who decline enrollment and their reasons for declining. We will also examine retention/dropout from the study and reasons, length/frequency of DEC sessions, % of potential DEC sessions completed, and IVR call adherence (% attempted calls completed, # weeks adherent to calls). We will analyze facilitators and barriers to implementation among dyad and staff interview themes using the Consolidated Framework for Implementation Research (CFIR). The CFIR's five major domains are the intervention, inner and outer setting, the individuals involved and the implementation process.

15. Study Oversight, Quality Control

Study investigators and staff will be responsible for study oversight and maintaining the highest standards of intervention delivery throughout the study period. The principal investigator, Dr. Rosland, will maintain appropriate oversight of this research protocol and study staff, including recruitment, selection of study participants, study conduct, and delegation of research responsibilities. Bi-weekly meetings will be conducted throughout the study period to review all study activities. All study investigators will review study materials and protocols prior to the start of the intervention in order to provide input on best practices for managing patient safety and privacy. Additionally, the intervention will undergo annual and continuing review through the Ann Arbor IRB and comply with all yearly consent form audits as well as 3 year full regulatory audits. To remain prepared for regulatory audits, the project will maintain a regulatory binder which meets all regulatory requirements and is kept up to date throughout the study period.

16. Timeline

	Year 1				Year 2				Year 3				Year 4			
	Q1	Q2	Q3	Q4	Q1	Q2	Q3	Q4	Q1	Q2	Q3	Q4	Q1	Q2	Q3	Q4
	JJA	SON	DJF	MAM	JJA	SON	DJF	MAM	JJA	SON	DJF	MAM	JJA	SON	DJF	MAM
IRB Review																
Hire/Train Research Assistants																
Hire/Train Dyad Engagement Coach																
Refine and Finalize Study and Intervention Materials																
Participant Recruitment																
Participant Enrollment/Assessment																
12 Month Participant Assessment																
Qualitative Data Collection																
Data Analysis																
Write Reports/Manuscripts																
Dissemination Planning																

17. Ethics/Protection of Human Subjects

17.1 Risks to Subjects

17.1.1 Patient and Care Partner Participants

Physical risks: Patient participants will undergo venipuncture for HbA1C and lipid panels two times over 12 months. If patients have had an HbA1C performed at a [REDACTED]HS facility as part of routine care within 4 weeks prior their baseline study assessment date or 2 weeks before their 12-month study assessment date, or if they had a lipid panel performed within 4 weeks before an assessment date, that test result will be used for the study assessment and an additional study-associated venipuncture will not be performed. However, we estimate that at least 80% of patient participants will undergo a venipuncture that would not be required by routine clinical care. Risks of this minor procedure include brief pain, bruising, and minor bleeding. This study does not involve pharmacotherapy. Care Partner participants will not undergo venipuncture or blood pressure measurement.

Psychological risks: It is possible that some participants may find that being interviewed or audio-recorded is stressful. However, almost all the survey questions (and all of the questions that are related to sensitive issues such as depressive symptoms) have been used in multiple prior studies conducted by our team, and our participants have not reported that the questions increase their burden or anxiety.

Patients in the intervention group will receive biweekly automated tele-monitoring concerning their health. Although it is possible that automated calls would become burdensome or annoying, our preliminary studies suggest otherwise. This probability assessment is based upon the high rates of retention and call completion,

very high user satisfaction, and the fact that most patients desire that the automated calls become part of their standard care.

It is possible that participation in the CO-IMPACT intervention may cause participant discomfort, strain patient-supporter relationships, strain patient-clinician relationships, or increase Care Partner burden. However, the intent of the intervention is to better support patients and family members, decrease their diabetes and caregiving related stress, and improve the quality of diabetes-related communication between the patient and the supporter and between the patient and their clinicians. There was no indication in the CO-IMPACT pilot study, or in multiple previous studies of patient-supporter IVR interventions, that burden was increased or relationships were strained.

There is a small risk that patients will regret sharing certain medical or personal information with their Care Partner. However, we are stipulating that the supporter should be someone who is already regularly involved in the patient's health care. In our pilot studies with [REDACTED]HS patients with high-risk diabetes, there was minimal concern about sharing health information with a close family member or friend who was already involved in the patient's health care.

In the baseline and 12-month assessments, both patients and Care Partners will be assessed for depression using the Patient Health Questionnaire (PHQ-4).[61,62] Those who score 6 or above will be referred to a primary care doctor. If the participant is a Care Partner and does not have a primary care provider, they will be given contact information for mental health providers or referral services that are located near the participant's home. In the baseline for patients, patients will be assessed for PTSD using the Primary Care PTSD Screen for DSM5 (PC-PTSD-5)[60], and those who endorse a prior traumatic event and at least 3 of the 6 symptoms in the last month will be referred to their primary care provider, who can refer the patient to Primary Care Mental Health or the PTSD Clinic Team, according to VA clinical guidelines.[72] Project staff will also be trained to follow standard protocols if they detect a patient is a high suicide risk, including a warm handoff to the VA Suicide Prevention Hotline.

Social and Legal: These risks include potential breach of confidentiality, inadvertent release of sensitive information, and the risk of participation due to potential coercion.

17.2 Adequacy of protections against risks

Recruitment and Informed Consent

We will request a HIPAA informed consent waiver to perform searches to identify potential participants based on: "38 CFR 16.116 (d) (2): This research presents no more than minimal risk of harm to the subjects, the waiver will not adversely affect the rights and welfare of the subjects, and this research could not be practicably carried out without a waiver." This search protocol involves no more than minimal risk and use of secondary data will not adversely affect patients' rights. The research could not be carried out without the waiver because without access to the electronic medical record data, we will have no way of identifying patients with high-risk diabetes that might benefit from participating in the trial. We have obtained waivers successfully for similar screening and recruitment approaches in several previous and ongoing studies. We will seek informed consent from any potential participants prior to baseline assessment and randomization.

Identified patients will be sent an introductory letter informing them about the study and inviting them to participate. The letter will include a study phone number and language indicating that they can opt out of further contact by calling study staff via a toll-free number or by mailing a form that is printed on the back of the letter in a postage-paid, addressed envelope that is included with the letter. The outside of the envelope will not contain anything that indicates the nature of the research. In the absence of such notification, 7-10 days after the letter is expected to arrive, study staff will call patients to explain the study in more detail, conduct initial screening and if eligible, ask if they wish to learn more about participating. During the initial contact call, the study will be described and patients will have ample and repeated opportunities to ask questions.

Patients who are deemed eligible and continue to express interest in the study after screening will be invited to an in-person informed consent and enrollment visit at the [REDACTED]VA. At that time, study staff will describe the content of the study in detail, including that patients and Care Partners can decline participation in the study at any time or decline Care Partner participation in the patient's health care at any time. Patients will also be told in detail the type of clinical information that Care Partners may have access to should they be randomized to the intervention group. After any and all patient questions are encouraged and answered, patients will be invited to sign an informed consent form and HIPAA form consenting to release their PHI to study staff. If randomized to the intervention, patients will be invited to sign a standard [REDACTED]VA Release of Information form to give permission for VA staff to share personal health information with their Care Partner.

During the initial screening phone call to the patient, willing and eligible patients will be asked to identify a Care Partner to participate in the study with them. Eligible patients will be encouraged to contact the potential Care Partner to explain their interest in the study, and concurrently, the RA will send a letter to the potential Care Partner that briefly introduces the study. After about a week, the RA will call the potential Care Partner to screen for interest and eligibility. If the individual is interested and eligible, they will be asked for verbal consent following a clear protocol. We anticipate that in some rare cases, the patient will not have the potential Care Partner's mailing address or email address; in such cases, study staff will send the materials to the patient and ask that they deliver them to the prospective Care Partner. After a waiting period determined by the patient, the study staff will call the potential Care Partner. We received IRB approval to verbally consent Care Partners in the CO-IMPACT pilot and other previous studies involving Veterans' Care Partners based on the following: 1) in the control arm, Care Partners will undergo only limited assessment, 2) in the intervention arm, Care Partner involvement will be limited to receiving information about patient's diabetes status and care plans, and guidelines to discussing diabetes care with patients. We have used this same process in prior studies and have found that it is an efficient and effective way to recruit large samples of Veterans and their Care Partners.

Because our outcome evaluation is guided by the RE-AIM framework, it is important for us to assess the intervention's "reach" among potentially eligible Patients or Care Partners. Thus, Patients and Care Partners who decline to participate will be asked to volunteer to answer 3-4 questions over the phone about their sociodemographics (age, race/ethnicity, distance from the VA) and reasons for not participating.

17.3 Protection against Risk

17.3.1 Patient and Care Partner Participants

Physical Risks:

To minimize risk, venipuncture will be performed by [REDACTED]HS trained laboratory phlebotomists in [REDACTED]HS facilities. Patient participants who have recently had the relevant laboratory test done as part of routine care will not be asked to repeat it for the study assessment. If patients have had an HbA1c performed at a [REDACTED]HS facility as part of routine care within 4 weeks prior their baseline study assessment date, or 2 weeks before their 12-month study assessment date, or if they had a lipid panel performed within 4 weeks before an assessment date, that test result will be used for the study assessment and an additional study-associated venipuncture will not be performed.

Blood work ordered at baseline and 12-month follow-up will be ordered under the Principal Investigator's name, a practicing primary care physician at [REDACTED]VA. She will review all results and alert the patient's assigned primary care physician if there are any unexpected results.

The RAs will be trained by the PI, a practicing primary care physician, on blood pressure measurement using an automatic cuff. If either number of the patient's blood pressure is <90/50 or >180/110, the RA will escort the patient to the (on-site) VA urgent care provider. If either number of the patient's blood pressure is >140/90 but <180/110, the RA will recommend that the patient call his or her primary care doctor, and the RA will also contact the patient's team the same day by adding a note in the patient's chart and adding their PACT RN as a cosigner.

Psychological Risks:

Assessment Burden: Patients and Care Partners will be informed as part of their informed consent process and immediately prior to each interview that they can refuse to answer any questions in the assessments, or drop out of the study at any time.

In the baseline assessments, both patients and Care Partners will be assessed for depression using the Patient Health Questionnaire (PHQ-4). Those who score 6 or above will be referred to a primary care doctor. If the participant is a Care Partner and does not have a primary care provider, they will be given contact information for mental health providers or referral services that are located near the participant's home. Project staff will also be trained to follow standard protocols if they detect a patient is a high suicide risk, including a warm handoff to the VA Suicide Prevention Hotline.

Patient-Care Partner Relationship:

We will reduce the risk of patient-Care Partner conflicts through several strategies. These include the following:

We specifically structure the Care Partner's role as assistive to the patient. This is conveyed repeatedly in study contacts. For example, Care Partners are instructed to discuss any concerns with patients in a non-judgmental manner, and to offer choices. They are instructed to encourage the patient to be the main contact for the patient's health care providers whenever possible. Our pilot study experiences suggest that under these arrangements, patients welcome the supporter's instrumental and emotional support. Clear and redundant presentation through printed participant guidelines for patients and Care Partners to structure their roles encourage effective communication. This information is repeated in smaller chunks throughout the study timeline.

Patient willingness to share their health information with their Care Partner will be explicitly confirmed in their written consent. We will thoroughly explain to patients the type of information that will be included in Dyad Engagement Coach contacts with Care Partners. To minimize risk of patient regret over sharing health information, we are stipulating that the Care Partner should be someone whom is already regularly involved in the patient's health care. The study team will not share information with a Care Partner before it is shared directly with the patient. Information that is directly related to diabetes management and management of risk of diabetes complications will be emphasized in communications between the study team and Care Partners. However, we will inform patient participants that standard VA clinic printouts of medication lists or appointments may contain information related to potentially sensitive topics, such as psychiatric care or sexual health. The Dyad Engagement Coach will be thoroughly trained by the PI in protocols for extracting diabetes-relevant data from the medical record, and extraction will be tested prior to intervention start, and monitored by the PI throughout the intervention through a random sample of DEC documents (sampled more frequently at the beginning of the study). Patients and Care Partners will be reminded at every study contact that they can decline participation at any time and that the patient can terminate Care Partner participation in care at any time.

Care Partner Caregiving Burden:
A standard measure to assess Care Partner burden will be administered to each Care Partner participant at study baseline and endpoint. Prior studies involving chronically ill Veterans and family supporters, including the pilot study of this intervention, show that Care Partner burden does not increase, and often decreases, as the result of intervention. We will include on our study website a link to the National VA Caregiver's Program page on reducing caregiver stress.

Patient-Clinician Relationship and Clinical Care:
One of the goals of our intervention is to relieve busy clinicians from some of the day-to-day problem-solving that some patients request and require. Nevertheless, it is critical that patients and Care Partners understand that the intervention is not intended to be a substitute for professional-level formal caregiving, and that they should not attempt to address every problem identified via the assessments without input from the patient's clinical team. We will employ several strategies to ensure timely and appropriate use of formal health services when indicated:

- individual patient assessment calls will include explicit reminders about the importance of contacting their clinician if their health deteriorates.
- We included an option for patients to hear the number for their VA clinician's clinic call center at the end of each call.
- Content for Care Partners will emphasize the importance of the patient's health care relationship with their VA clinicians, and supporters will be instructed to encourage the patient to contact clinicians directly rather than having the supporter serve as a communication intermediary. However, supporters will also receive the clinicians' name and phone numbers.
- The binder and website will also emphasize that chronic illness care is most effective when patients take an active role in their care, and we will provide concrete guidance regarding effective patient - provider communication.

The study will be described to all provider and clinical staff at staff meetings. Providers will be allowed to opt out of the study, thus making patients assigned to their primary care panel ineligible for the study.

Although risks of worsening symptoms will be minimal, patients will receive additional clinical monitoring than would be provided as part of routine clinical care. Therefore, any potential adverse effects of the intervention will likely be detectable through the increased monitoring and contact provided by the intervention. All study staff will be thoroughly trained on how to address patients found to be experiencing worsening diabetes outcomes or psychological distress during study-related contacts. Patients will continue to have access to all usually available health care services.

If patients or Care Partners discuss *non-urgent* health concerns with the Dyad Engagement Coach, the coach will encourage them to discuss their concerns with their patient partner and record the concerns for discussion with their provider at their next appointment. If patients or supporters discuss *urgent* health concerns, the health educator will recommend that they call their health care provider immediately, and, for VA patients, offer to connect them directly by phone to the appropriate primary care team, if during usual business hours. Dyad Engagement Coach will be trained to call 911 for any emergency situations they encounter.

During IVR phone assessments, an automated fax alert to the patient's PACT nurse care manager will be generated if clinically urgent issues are identified. IVR assessments will not ask about symptoms that constitute medical emergencies (i.e. chest pain, loss of consciousness), but patients will be reminded in relevant calls and in study printed materials to hang up and call 911 if they experience a medical emergency.

Coercion:
Staff training will be employed to minimize risk of potential coercion.

Social and Economic Risks: Multiple, stringent measures to protect confidentiality and prevent inadvertent release of sensitive information will be implemented.

We will obtain written informed consent from each study participant. As part of that consent, participants will be adequately informed about the small risk of a breach of confidentiality and they will be given the option of opting out of participation. Throughout the study, IRB and HIPAA guidelines will be followed to ensure the privacy and integrity of the information we collect. All study staff will have signed a pledge of confidentiality and are trained annually in secure handling of VA research data according to HIPAA and human subjects guidelines. Any breach of confidentiality will be immediately reported to the PI and to the VA Ann Arbor Healthcare System Human Subjects Committees (and as required to any other IRBs). In addition, any complaints or concerns expressed to the study staff by participants, providers, or anyone else affected by this study will be immediately reported to the PI and the IRB.

For individuals who decide to not be screened for the study, express interest but decide not to consent, or are lost to follow-up prior to providing written informed consent, we will retain only the screening data for these individuals using an unlinkable identifier so that we can characterize non-participants for scientific reasons. Essentially, no screening data will be linkable to other research or clinical data unless the participant provides written informed

consent, and no additional data will be collected on participants who provide informed consent but are later determined to be ineligible to participate, with the exception of a coded identifier on a “do not contact” list maintained only for the duration of the recruitment phase in order to prevent such individuals from being re-solicited.

To protect against confidentiality breach, we will follow confidentiality procedures throughout the study and afterwards.-The database linking participants’ unique study identification numbers with their Personally Identifying information will be kept in a study-specific, access-restricted folder on the OI&T server behind the VA firewall. HSR&D will maintain all research records containing VA sensitive information (VASI) in accordance with 36 CFR 1228, Subpart D, until instructions on when to destroy them are approved by the National Archives and Records Administration and are published in VHA's Records Control Schedule (RCS 10-1). Until that time, records will be kept on the HSR&D drive on the OI&T server behind the VA firewall and will be destroyed according to the new Records Control Schedule once it is published.

Some Dyad Engagement Coach documentation might become part of patients' medical records, and therefore subject to current clinical data confidentiality regulations. No participant-level data, including identifiers and individual data points, will be published. PII of those participants who do not enroll will be moved to an access restricted folder on the Ann Arbor VA OI&T network.

Confidentiality of the Intervention Website
The public website hosted at Amazon Web Services (AWS) GovCloud will be used by patient participants, Care Partners and study staff to access pre-visit worksheets, after-visit summaries, reports, and health education resources. The only personalized information available on the website will be PDFs of Visit Summaries--short summaries of any diabetes-related test results and what happened during a patient’s clinical visit to their provider at the VA. These visit summaries do not contain any personally identifying information about the patient or care partner. Information that is related to diabetes management and management of the risk of diabetes complications will be emphasized in the Visit Summary. However information shared with Care Partners may include medication or appointments lists that pertain to potentially sensitive topics such as psychiatric care or sexual health.

The program website is separate from any electronic medical records or other data storage devices, and there will be no access to other patient-level PHI via the website or server. All patient data will be de-identified. Patients in the research project will sign an informed consent document which details how their data will be transmitted and stored.

Server Software
InterVision Media (IVM) will manage and support the software infrastructure.

Datacenter Infrastructure

The server will be hosted at the Amazon Web Services (AWS) GovCloud

AWS GovCloud (US) is an isolated AWS region designed to host sensitive data and regulated workloads in the cloud, helping customers support their U.S. government compliance requirements, including the International Traffic in Arms Regulations (ITAR) and Federal Risk and Authorization Management Program (FedRAMP). AWS GovCloud (US) is operated by employees who are vetted "U.S. Persons" and root account holders of AWS accounts must confirm they are U.S. Persons before being granted access credentials to the region.

AWS’ data centers are state of the art, utilizing innovative architectural and engineering approaches. AWS has many years of experience in designing, constructing, and operating large-scale data centers. This experience has been applied to the AWS platform and infrastructure. AWS data centers are housed in nondescript facilities. Physical access is strictly controlled both at the perimeter and at building ingress points by professional security staff utilizing video surveillance, intrusion detection systems, and other electronic means. Authorized staff must pass two-factor authentication a minimum of two times to access data center floors. All visitors and contractors are required to present identification and are signed in and continually escorted by authorized staff.

Data Transmission Security

In this project, a public website is used by CarePartners and study staff. AWS GovCloud (US) is configured with multiple virtualized hosting environments. A firewall isolates each environment from all others. All data transmissions are protected by an SSL (Secure Sockets Layer) certificate using AES 256-bit encryption.

Data storage security and backup
All data is encrypted at rest by the application—using an AES-256 cipher—before it is saved to the storage partition. Furthermore, partition encryption is provided at the OS level using a FIPS 140-2 kernel-level disk volume encryption method. All system data is encrypted and backed up nightly to a separate server within the isolated network.

Firewall
An Internet firewall provides monitors and controls incoming and outgoing network traffic, providing protection from intrusions, attacks and other unauthorized access.

Confidentiality of the IVR System
The IVR system, an automated phone system, is separate from any electronic medical records or other data storage devices, and separate from the Intervention Website described above, and there will be no access to other outside patient-level PHI via the website or server. Patients in the research project will sign an informed consent document which details how their data will be transmitted and stored. The portal hosted at the Amazon Web Services (AWS) GovCloud will be used to manage the IVR system, including patient registration, scheduling, monitoring, and reporting. Access to the portal is password protected, using unique 'strong' passwords. Data stored on the site will be limited to patients' first and last names, year of birth, telephone numbers, and Care Partners' email address, which will be entered through a web interface by study personnel only. All patient responses to IVR call questions will be stored as numerical data, further limiting the patient-level data on the system.

Server Software
InterVision Media (IVM) will manage and support the software infrastructure.

Datacenter Infrastructure

> The server will be hosted at AWS GovCloud (US). AWS GovCloud (US) is an isolated AWS region designed to host sensitive data and regulated workloads in the cloud, helping customers support their U.S. government compliance requirements, including the International Traffic in Arms Regulations (ITAR) and Federal Risk and Authorization Management Program (FedRAMP). AWS GovCloud (US) is operated by employees who are vetted "U.S. Persons" and root account holders of AWS accounts must confirm they are U.S. Persons before being granted access credentials to the region.
>
> AWS' data centers are state of the art, utilizing innovative architectural and engineering approaches. AWS has many years of experience in designing, constructing, and operating large-scale data centers. This experience has been applied to the AWS platform and infrastructure. AWS data centers are housed in nondescript facilities. Physical access is strictly controlled both at the perimeter and at building ingress points by professional security staff utilizing video surveillance, intrusion detection systems, and other electronic means. Authorized staff must pass two-factor authentication a minimum of two times to access data center floors. All visitors and contractors are required to present identification and are signed in and continually escorted by authorized staff.

Data Transmission Security

In this project, a private portal will facilitate patient IVR calls and is accessed only by the study staff. No public access is permitted to the private website portal. It can only be accessed after an authorized user first establishes a FIPS 140-2 compliant Virtual Private Network (VPN) connection. All subsequent data transmissions, including all data collected by the IVR system, are further protected by an SSL (Secure Sockets Layer) certificate using AES 256-bit encryption.

Telecommunications
IVM's telecommunications provider, NexVortex, provisions and supports the SIP trunks that carry all VoIP traffic. NexVortex's Service Assurance Manager (SAM) allows monitoring, analysis, and response to IP network changes in real time. The system provides both outgoing as well as toll-free incoming phone calls, and supports cell phones and fax machines both domestically and internationally.

Data storage security and backup
All data is encrypted at rest by the application—using an AES-256 cipher—before it is saved to the storage partition. Furthermore, partition encryption is provided at the OS level using a FIPS 140-2 kernel-level disk volume encryption method. All system data is encrypted and backed up nightly to a separate server within the isolated network.

Firewall
An Internet firewall monitors and controls incoming and outgoing network traffic, providing protection from intrusions, attacks and other unauthorized access. Connections between authorized VA personnel and AWS GovCloud (US) are available only via the firewall's VPN, configured with a validated FIPS 140-2 crytographic module.

Confidentiality of Assessment Data
It will be made clear to participants that no information gathered through study baseline, 6-month and 12-month assessments will be shared with the other member of the dyad (i.e. patient assessment information will not be shared with the patient's Care Partner and vice versa). None of the information provided to research assistants will be shared with participants' clinicians unless the patient appears to be in danger (in cases of suicidality, for example) and Dr. Rosland deems it necessary to contact the participant's physician.

Assessment data collected face-to-face or by mail will be collected by paper survey and then entered into a study assessment database on the VA restricted sever. When assessments take place by phone interview, the study staff will enter the data directly into a study assessment database on the VA restricted sever. Assessment data will be linked to the participant's study ID number but not to PII. Data captured on paper will be stored in secure, locked cabinets in the research office space.

After data collection is complete, de-identified data from the patient participants (not the Care Partner participants) will be shared with Insignia, the company that holds the rights to the Patient Activation Measure (PAM). (Insignia normally requires an annual fee $7,500 for a license, which is not within our study's budget. However, Insignia provides a reduced or waived fee for organizations that are willing to share deidentified data.) This de-identified data includes, but is not limited to the same dataset in which the PAM is used. Jenny Davis, HSR&D's data manager, will de-identify the dataset, write the data on an encrypted CD rom disc, and send to Insignia. Its contents will be only accessible by the intended recipient.

Confidentiality of Audio Recordings for Quality Assurance
Audio-recording of selected coaching sessions is necessary to ensure the fidelity of the coaching sessions. Participants, including Care Partners, will be asked to provide to verbal consent to the audiorecording immediately prior to the session. They will explain to participants that the recordings are made for quality assurance purposes, notified that they may participate in the study even if they do not wish to be audio recorded, and that the audio recording will be stopped at any time they wish. They will then state their consent to being recorded, as they are recorded. Participants who decline to be audio-recorded will complete the scheduled coaching session as usual without audio-recording. Each digital audio file will be labeled only with the participant's study ID#. Recording devices will be kept physically secure in a locked drawer or locked transport case at all times, and once audio files are uploaded to the secure servers behind the VA firewalls, they will be deleted from the DVRs.

17.4 Potential Benefits

Potential benefits of the proposed research to the subjects and others
This study will evaluate an innovative method for improving informal care support through engaging patients' Care Partners and enhancing the effectiveness of their support. We expect this study to produce an evidence-based protocol and tools that engage VA patients with high-risk diabetes and their Care Partners in PACT to help PACT achieve the best diabetes outcomes. This protocol could then be implemented in PACT encounters with high-risk patients throughout the VA.
Many intervention-assigned participants are likely to experience direct benefits from participation in this study:

- All intervention-assigned patient participants will receive twice-monthly automated telephone assessment of their diabetes symptoms and self-care. This may improve the quality of their diabetes care, and their diabetes outcomes.
- Patient participants may experience additional benefits to their health and well-being as a result of feedback also being sent to the Care Partner whom they nominate.
- Coaching on patient activation skills may improve patient communication with their PACT clinicians, and result in improved diabetes clinical care.
- Control-assigned participants will receive physician-approved information on diabetes management.

We expect that this study will contribute to the growing body of knowledge on how VA can most effectively engage family supporters and Care Partners in patients' care to optimize health management and outcomes. By improving informal care support for Veterans with diabetes, this research may indicate new ways to improve their health, health-related quality of life, health behaviors, and reduce diabetes-related distress. Diabetes is prevalent among Veterans and often results in substantial morbidity, distress, disability, and health care costs. We feel the potential knowledge to be gained outweighs the low risk of minimal harm to participants.

Because diabetes is prevalent among Veterans and results in substantial morbidity, health care utilization and costs, we feel the study's potential benefits outweigh the low risk of minimal harm to participants.

18. Data and Safety Monitoring Plan

Institutional Review Board (IRB) approvals will be obtained at the Ann Arbor VAMC. The PI, Dr. Ann-Marie Rosland, will take ultimate responsibility for ensuring the safety of the participants. Regular study team meetings will be used to ensure that all study protocols and IRB policies and procedures are being followed. This will include ensuring that;

1. All participants understand, agree to, and sign a written consent form before participating
2. Strict adherence is maintained to communication regarding the participants' right to withdraw or refuse to answer questions
3. Staff maintain confidentiality both by protecting hard-copy and electronic data collection forms and also by avoiding all unauthorized conversations about individual patients
4. Consent forms and identifying information are kept separately from study related information about patients' sociodemographics, clinical characteristics, disease self-care, service use, and outcomes
5. All hard copies containing identifying information is kept locked at all times, and sensitive computer files are maintained on a secured VA server with access limited to approved study staff
6. Coding for ambiguous responses is handled in a way that is consistent and clear across data collectors and over time
7. Participants are informed in writing how to contact the study PI, the study coordinator, and the relevant IRB office with any questions or concerns.

All AEs, problems, and protocol deviations/violations will be brought to the immediate attention of Dr. Rosland. Dr. Rosland will be responsible for reporting all serious adverse events (SAEs), adverse events (AEs), and serious problems, as defined in the policy entitled *VA IRB policy, analysis, and reporting form for serious adverse events, serious problems, protocol deviations, and other reportable events to participants and others in human subjects research* (last updated 9.8.16). Such events or problems may be identified through participant report via the study toll-free number, or research staff viewing in medical records in the course of planned study activities. Specifically, death and life-threatening SAEs will be reported to the [REDACTED]HS IRB by phone

immediately following the discovery of the event, with a completed *Serious Adverse Event, Serious Problem, Protocol Deviation, and Other Reportable Event Investigator Reporting Form VA Ann Arbor Healthcare System Subcommittee on Human Studies (151)* form submitted within 5 business days following the discovery of the event, problem or information that involves VA research. Other SAEs, AEs, and serious problems that are possibly related to research and unanticipated will be reported to the [REDACTED]HS IRB within 5 business days of the discovery via the form named above. This includes suspension or termination of research activities. Protocol deviations will be reported to the VA IRB within 30 days, in memo format.

Reports of non-serious unanticipated events, problems and protocol deviations will be submitted in tabular form at each annual continuation review, and other events reported immediately, within 5 days, and within 30 days will be included. If any uncertainty exists regarding reporting, the PI will consult with the IRB coordinator.

19. Inclusion of Women, Minorities and/or Children

Children will not be eligible for this study, as either patients or Care Partners. We will recruit eligible patients and Care Partners who give informed consent, regardless of their gender, race, or ethnicity. We expect the study to reflect the racial, ethnic, and gender distribution of the study sites. Representation of racial minority groups and women will be monitored throughout the project, and if it appears that they are underrepresented among participants, significant efforts will be made to boost their enrollment. These efforts will include qualitative interviews with women and minorities who are and are not participating to understand barriers to participation and to learn new strategies for increasing representation of those groups.

References

1. Hibbard JH, Greene J. What the evidence shows about patient activation: better health outcomes and care experiences; fewer data on costs. *Health Aff Proj Hope* 2013;32:207–214.
2. Rosland A-M, Heisler M, Choi H-J, et al. Family influences on self-management among functionally independent adults with diabetes or heart failure: do family members hinder as much as they help? *Chronic Illn* 2010;6:22–33.
3. Silliman RA, Bhatti S, Khan A, et al. The care of older persons with diabetes mellitus: families and primary care physicians. *J Am Geriatr Soc* 1996;44:1314–1321.
4. Rosland A-M, Piette JD, Choi H, Heisler M. Family and friend participation in primary care visits of patients with diabetes or heart failure: patient and physician determinants and experiences. *Med Care* 2011;49:37–45.
5. Wolff JL, Roter DL. Family presence in routine medical visits: A meta-analytical review. *Soc Sci Med* 2011;72:823–31.
6. Strom JL, Egede LE. The impact of social support on outcomes in adult patients with type 2 diabetes: a systematic review. *Curr Diab Rep* 2012;12:769–781.
7. Luttik ML, Jaarsma T, Moser D, et al. The importance and impact of social support on outcomes in patients with heart failure: an overview of the literature. *J Cardiovasc Nurs* 2005;20:162–9.
8. Lett HS, Blumenthal JA, Babyak MA, et al. Social support and coronary heart disease: epidemiologic evidence and implications for treatment. *Psychosom Med* 2005;67:869–78.
9. Nicklett EJ, Heisler MEM, Spencer MS, Rosland A-M. Direct Social Support and Long-term Health Among Middle-Aged and Older Adults With Type 2 Diabetes Mellitus. *J Gerontol B Psychol Sci Soc Sci* 2013;68:933–943.
10. Gallant M. The influence of social support on chronic illness self-management: a review and directions for research. *Health Educ Behav* 2003;30:170–195.
11. Hibbard JH, Greene J, Overton V. Patients with lower activation associated with higher costs; delivery systems should know their patients' "scores." *Health Aff Proj Hope* 2013;32:216–222.
12. Hibbard JH, Stockard J, Mahoney ER, Tusler M. Development of the Patient Activation Measure (PAM): conceptualizing and measuring activation in patients and consumers. *Health Serv Res* 2004;39:1005–1026.
13. Carman KL, Dardess P, Maurer M, et al. Patient and family engagement: a framework for understanding the elements and developing interventions and policies. *Health Aff Proj Hope* 2013;32:223–231.
14. Parchman ML, Zeber JE, Palmer RF. Participatory decision making, patient activation, medication adherence, and intermediate clinical outcomes in type 2 diabetes: a STARNet study. *Ann Fam Med* 2010;8:410–417.

15. Giardina TD, King BJ, Ignaczak AP, et al. Root cause analysis reports help identify common factors in delayed diagnosis and treatment of outpatients. *Health Aff Proj Hope* 2013;32:1368–1375.
16. Hibbard JH, Cunningham PJ. How engaged are consumers in their health and health care, and why does it matter? *Res Brief* 2008:1–9.
17. Maeng DD, Martsolf GR, Scanlon DP, Christianson JB. Care coordination for the chronically ill: understanding the patient's perspective. *Health Serv Res* 2012;47:1960–1979.
18. Kinnersley P, Edwards A, Hood K, et al. Interventions before consultations to help patients address their information needs by encouraging question asking: systematic review. *BMJ* 2008;337:a485.
19. Deen D, Lu W-H, Rothstein D, et al. Asking questions: the effect of a brief intervention in community health centers on patient activation. *Patient Educ Couns* 2011;84:257–260.
20. Greenfield S, Kaplan SH, Ware JE Jr, et al. Patients' participation in medical care: effects on blood sugar control and quality of life in diabetes. *J Gen Intern Med* 1988;3:448–457.
21. Kahwati LC, Lance TX, Jones KR, Kinsinger LS. RE-AIM evaluation of the Veterans Health Administration's MOVE! Weight Management Program. *Transl Behav Med* 2011;1:551–560.
22. Connell CM. Psychosocial contexts of diabetes and older adulthood: reciprocal effects. *Diabetes Educ* 1991;17:364–71.
23. Rosland A-M, Heisler M, Janevic MR, et al. Current and potential support for chronic disease management in the United States: the perspective of family and friends of chronically ill adults. *Fam Syst Health J Collab Fam Healthc* 2013;31:119–131.
24. Piette JD, Rosland AM, Silveira M, et al. The case for involving adult children outside of the household in the self-management support of older adults with chronic illnesses. *Chronic Illn* 2010;6:34–45.
25. Wolff JL, Roter DL. Hidden in plain sight: medical visit companions as a resource for vulnerable older adults. *Arch Intern Med* 2008;168:1409–1415.
26. Janevic MR, Rosland A-M, Wiitala W, et al. Providing support to relatives and friends managing both chronic physical illness and depression: the views of a national sample of U.S. adults. *Patient Educ Couns* 2012;89:191–198.
27. Anderson ES, Wojcik JR, Winett RA, Williams DM. Social-cognitive determinants of physical activity: the influence of social support, self-efficacy, outcome expectations, and self-regulation among participants in a church-based health promotion study. *Health Psychol* 2006;25:510–20.
28. Coffman MJ. Effects of tangible social support and depression on diabetes self-efficacy. *J Gerontol Nurs* 2008;34:32–9.
29. DiIorio C, Shafer PO, Letz R, et al. Project EASE: a study to test a psychosocial model of epilepsy medication management. *Epilepsy Behav* 2004;5:926–36.
30. Cotter KA, Sherman AM. Love hurts: the influence of social relations on exercise self-efficacy for older adults with osteoarthritis. *J Aging Phys Act* 2008;16:465–83.
31. Rosland A-M, Kieffer E, Israel B, et al. When is social support important? The association of family support and professional support with specific diabetes self-management behaviors. *J Gen Intern Med* 2008;23:1992–1999.
32. Nicklett EJ, Liang J. Diabetes-Related Support, Regimen Adherence, and Health Decline Among Older Adults. *J Gerontol Ser B-Psychol Sci Soc Sci* 2009.
33. Clayton JM, Butow PN, Tattersall MHN, et al. Randomized controlled trial of a prompt list to help advanced cancer patients and their caregivers to ask questions about prognosis and end-of-life care. *J Clin Oncol Off J Am Soc Clin Oncol* 2007;25:715–723.
34. Piette JD, Rosland A-M, Marinec NS, et al. Engagement with automated patient monitoring and self-management support calls: experience with a thousand chronically ill patients. *Med Care* 2013;51:216–223.
35. Rohrbaugh MJ, Shoham V, Coyne JC, et al. Beyond the "self" in self-efficacy: Spouse confidence predicts patient survival following heart failure. *J Fam Psychol JFP J Div Fam Psychol Am Psychol Assoc Div 43* 2004;18:184–193.
36. Rosland A-M, Piette JD. Emerging models for mobilizing family support for chronic disease management: a structured review. *Chronic Illn* 2010;6:7–21.
37. Griffin JM, Meis L, Greer N, et al. Effectiveness of Family and Caregiver Interventions on Patient Outcomes Among Adults with Cancer or Memory-Related Disorders: A Systematic Review. Washington (DC): Department of Veterans Affairs 2013. Available at: http://www.ncbi.nlm.nih.gov/books/NBK148475/. Accessed December 5, 2013.
38. Dunbar SB, Clark PC, Deaton C, et al. Family education and support interventions in heart failure: a pilot study. *Nurs Res* 2005;54:158–66.

39. Burke JA, Earley M, Dixon LD, et al. Patients with diabetes speak: exploring the implications of patients' perspectives for their diabetes appointments. *Health Commun* 2006;19:103–14.
40. VHA Patient Centered Medical Home Model (PACT) Concept Paper. 2011.
41. Lorig K, Stewart A, Ritter P, et al. Outcomes Measures for Health Education and other Health Care Interventions. Thousand Oaks, CA: Sage Publications 1996.
42. Rosland A-M, Heisler M, Piette JD. The impact of family behaviors and communication patterns on chronic illness outcomes: a systematic review. *J Behav Med* 2012;35:221–239.
43. Ladhani NN, Majumdar SR, Johnson JA, et al. Adding pharmacists to primary care teams reduces predicted long-term risk of cardiovascular events in type 2 diabetic patients without established cardiovascular disease: results from a randomized trial. *Diabet Med J Br Diabet Assoc* 2012;29:1433–1439.
44. Webb DR, Khunti K, Gray LJ, et al. Intensive multifactorial intervention improves modelled coronary heart disease risk in screen-detected Type 2 diabetes mellitus: a cluster randomized controlled trial. *Diabet Med J Br Diabet Assoc* 2012;29:531–540.
45. Turner BJ, Hollenbeak CS, Liang Y, et al. A randomized trial of peer coach and office staff support to reduce coronary heart disease risk in African-Americans with uncontrolled hypertension. *J Gen Intern Med* 2012;27:1258–1264.
46. Benner JS, Erhardt L, Flammer M, et al. A novel programme to evaluate and communicate 10-year risk of CHD reduces predicted risk and improves patients' modifiable risk factor profile. *Int J Clin Pract* 2008;62:1484–1498.
47. Edelman D, Oddone EZ, Liebowitz RS, et al. A multidimensional integrative medicine intervention to improve cardiovascular risk. *J Gen Intern Med* 2006;21:728–734.
48. van Dieren S, Beulens JWJ, Kengne AP, et al. Prediction models for the risk of cardiovascular disease in patients with type 2 diabetes: a systematic review. *Heart Br Card Soc* 2012;98:360–369.
49. Toobert DJ, Hampson SE, Glasgow RE. The summary of diabetes self-care activities measure: results from 7 studies and a revised scale. *Diabetes Care* 2000;23:943–950.
50. Steiner JF, Prochazka AV. The assessment of refill compliance using pharmacy records: methods, validity, and applications. *J Clin Epidemiol* 1997;50:105–116.
51. McGuire BE, Morrison TG, Hermanns N, et al. Short-form measures of diabetes-related emotional distress: the Problem Areas in Diabetes Scale (PAID)-5 and PAID-1. *Diabetologia* 2010;53:66–69.
52. Davis K, Latty-Mann H. Love Styles and Relationship Quality: A Contribution to Validation. *J Soc Pers Relatsh* 1987;4:409–428.
53. Fitzgerald JT, Davis WK, Connell CM, et al. Development and validation of the Diabetes Care Profile. *Eval Health Prof* 1996;19:208–30.
54. Williams G, Lynch M, Ryan R, et al. Validation of the important other climate questionnaire: Assessing autonomy support for health-related change. *Fam Syst Health* 2006;24:179–194.
55. Bersheid E, Snyder M, Omoto A. The Relationship Closeness Inventory: Assessing the closeness of interpersonal relationships. *J Pers Soc Psychol* 1989;57:792.
56. Safran DG, Kosinski M, Tarlov AR, et al. The Primary Care Assessment Survey: tests of data quality and measurement performance. *Med Care* 1998;36:728–739.
57. Kaplan SH, Greenfield S, Gandek B, et al. Characteristics of physicians with participatory decision-making styles. *Ann Intern Med* 1996;124:497–504.
58. Charlson M, Szatrowski TP, Peterson J, Gold J. Validation of a combined comorbidity index. *J Clin Epidemiol* 1994;47:1245–1251.
59. Chew LD, Griffin JM, Partin MR, et al. Validation of screening questions for limited health literacy in a large VA outpatient population.[see comment]. *J Gen Intern Med* 2008;23:561–6.
60. Prins A, Bovin M, Smolenski D, et al. The primary care PTSD screen for DSM-5 (PC-PTSD-5): development and evaluation within a veteran primary care sample. *J Gen Intern Med* 2016;31:1206–1211.
61. Lowe B, Wahl I, Rose M, et al. A 4-item measure of depression and anxiety: Validation and standardization of the Patient Health Questionnaire-4 (PHQ-4) in the general population. *J Affect Disord* 2010;122:86–95.
62. Kroenke K, Spitzer R, Williams J, Lowe B. An ultra-brief screening scale for anxiety and depression: The PHQ–4. *Psychosomatics* 2009;50:613–621.
63. Robinson BC. Validation of a Caregiver Strain Index. *J Gerontol* 1983;38:344–348.
64. Cox DJ, Irvine A, Gonder-Frederick L, et al. Fear of hypoglycemia: quantification, validation, and utilization. *Diabetes Care* 1987;10:617–621.
65. Slone L. Prevalence f PTSD in primary care settings. *PTSD Res Q* 2016;17.

66. Hoerster K, Jakupcak M, Hanson R, et al. PTSD and depression symptoms are associated with binge eating among US Iraq and Afghanistan veterans. *Eat Behav* 2015;17:115–118.
67. Talbot L, Maguen S, Epel E, Neylan T. Post traumatic stress disorder is associated with emotional eating. *J Trauma Stress* 2013;26:521–525.
68. Kubzansky L, Bordelois P, Jun H, et al. The weight of traumatic stress: a prospective study of posttraumatic stress disorder symptoms and weight status in women. *JAMA Psychiatry* 2017;71:44–51.
69. Wolf E, Bovin M, Green J, et al. Longitudinal associations between post-traumatic stress disorder and metabolic syndrome severity. *Psychol Med* 2016;46:2215–2226.
70. Klingaman E, Hoerstrer K, Aakre J, et al. Veterans with PTSD report more weight loss barriers than veterans with no mental health disorders. *Gen Hosp Psychiartry* 2016;39:1–7.
71. Hoerster D, Lai Z, Goodrich D, et al. Weight loss after participation in a national VA weight management program among veterans with or without PTSD. *Psychiatry Serv* 2014;65:1385–1388.
72. The Management of Post-Traumatic Stress Working Group. VA/DoD Clinical Practice Guideline for Management of Post-Traumatic Stress 2010.
73. Kerr EA, Lucatorto MA, Holleman R, et al. Monitoring performance for blood pressure management among patients with diabetes mellitus: too much of a good thing? *Arch Intern Med* 2012;172:938–945.
74. Stata Statistical Software: Release 13. College Station, TX: StataCorp LP 2013.
75. Miller W, Crabtree B. Clinical research: a multimethod typology and qualitative roadmap. *Sage Publ* 1999.
76. Glasgow RE, Orleans CT, Wagner EH. Does the chronic care model serve also as a template for improving prevention? *Milbank Q* 2001;79:579–612, iv–v.
