# Supplementary material for: Effectiveness of a Health Coaching Intervention for Patient-Family Dyads to Improve Outcomes Among Adults With Diabetes: A Randomized Clinical Trial
Source: JAMA Netw Open. 2022 Nov 14;5(11):e2237960. doi: 10.1001/jamanetworkopen.2022.37960 (PMC9664266; doi:10.1001/jamanetworkopen.2022.37960)
Supplement: Supplement 2. — eTable 1. Key Study Measure Details eReferences eTable 2. Predictors of Enrollment Among the 1119 Patients With Diabetes Sent a Recruitment Letter eTable 3. Unadjusted Changes in Patient Outcomes by Study Arm, Baseline to 12 Months eTable 4. Intervention Effect on Baseline–to–12-Month Changes in Patient Outcomes From Multivariable Linear Regression, Full Model Results eTable 5. Patient and Supporter Ratings of Experience With CO-IMPACT Intervention Components eTable 6. Qualitative Themes Among CO-IMPACT Intervention Arm Patient and Supporter Participant Comments eFigure. CO-IMPACT IVR Topic Flow Chart eAppendix. CO-IMPACT Intervention Materials Toolkit Location [file jamanetwopen-e2237960-s002.pdf]

Supplementary Online Content

Rosland AM, Piette JD, Trivedi R, et al. Effectiveness of a health coaching intervention for patient-family dyads to improve outcomes among adults with diabetes: a randomized clinical trial. *JAMA Netw Open*. 2022;5(11):e2237960. doi:10.1001/jamanetworkopen.2022.37960

eTable 1. Key Study Measure Details

eReferences

eTable 2. Predictors of Enrollment Among the 1119 Patients With Diabetes Sent a Recruitment Letter

eTable 3. Unadjusted Changes in Patient Outcomes by Study Arm, Baseline to 12 Months

eTable 4. Intervention Effect on Baseline–to–12-Month Changes in Patient Outcomes From Multivariable Linear Regression, Full Model Results

eTable 5. Patient and Supporter Ratings of Experience With CO-IMPACT Intervention Components

eTable 6. Qualitative Themes Among CO-IMPACT Intervention Arm Patient and Supporter Participant Comments

eFigure. CO-IMPACT IVR Topic Flow Chart

eAppendix. CO-IMPACT Intervention Materials Toolkit Location

This supplementary material has been provided by the authors to give readers additional information about their work.

© 2022 Rosland AM et al. *JAMA Network Open*.

eTable 1. Key Study Measure Details

Outcome	Measure	Details
Pre-Specified Primary Patient Outcomes		
12-month change in patient activation	Patient Activation Measure (PAM)-13[1]	13-item survey with score range of 0 to 100; higher scores indicate greater activation
12-month change in diabetes-specific cardiac event risk	United Kingdom Prospective Diabetes Study (UKPDS) 5-year Risk Engine[2]	8-component measure with score range of 0 to 100; lower scores indicate lower predicted risk; components include HbA1c, SBP, lipid levels, age, sex, race/ethnicity, smoking status and years since diabetes diagnosis
Pre-Specified Secondary Patient Outcomes (all analyzed as 12-month change from baseline)		
Glycemic control	HbA1c, %	Venous sample in clinic laboratory
Blood pressure control	SBP, mmHg	Omron electronic automated oscillometric BP monitor, average of 2 clinic measures taken at least 5 minutes apart
Cholesterol control	Total cholesterol/HDL ratio	Venous sample in clinic laboratory
Smoking status	Global Adult Tobacco Survey (GATS)[3]	2-items: (a) current smoking status; (b) whether the respondent attempted to quit
Self-management behaviors<br>• Healthy eating<br>• Physical activity<br>• Blood sugar home testing<br>• Blood pressure home testing[a]<br>• Check feet<br>• Medication adherence	Summary of Diabetes Self Care Activities (SDSCA)[4]	14-items with individual item score range of 0 to 7 days of adhering to the healthy behavior in the past week; higher scores indicate more frequent adherence to behavior
Diabetes distress	Problem Areas in Diabetes Scale (PAID-5)[5]	5-item survey with score range of 0 to 20; higher scores indicate greater distress
Self-efficacy for diabetes management	Stanford Self-Efficacy for Diabetes Scale[6]	5-item survey with score range of 0 to 10; higher scores indicate greater self-efficacy
Efficacy in Healthcare Participation	Perceived Efficacy in Patient-Physician Interactions Scale (PEPPI-5)[7]	5-item survey with score range of 5 to 25; higher scores indicate greater participation in patient-healthcare professional interactions
Satisfaction with VA support for enrolled supporter	Original survey item	Over the last year, I have been satisfied with the VA's support of [Supporter]'s role in my health care,<br>Response options: Strongly Disagree (1), Disagree (2), Neither (3), Agree (4), Strongly Agree (5)
Other Participant Measures (Non-Outcomes)		
Health Literacy	Brief Health Literacy Screener[8]	3-items rated 0-4 each. Those whose answer rated 3 or 4 for any of the three items were designated as 'low health literacy', using cut-points demonstrated to have optimal specificity and sensitivity for identifying low health literacy.

HbA1c, hemoglobin A1c; SBP, systolic blood pressure; HDL, high-density lipoprotein

© 2022 Rosland AM et al. *JAMA Network Open*.

eReferences

[1]Hibbard JH, Stockard J, Mahoney ER, Tusler M. Development of the Patient Activation Measure (PAM): conceptualizing and measuring activation in patients and consumers. Health Serv Res. 2004;39(4 Pt 1):1005-1026. doi:10.1111/j.1475-6773.2004.00269.x

[2]Stevens RJ, Kothari V, Adler AI, Stratton IM, United Kingdom Prospective Diabetes Study (UKPDS) Group. The UKPDS risk engine: a model for the risk of coronary heart disease in Type II diabetes (UKPDS 56). Clin Sci Lond Engl 1979. 2001;101(6):671-679.

[3]Global Adult Tobacco Survey Collaborative Group. Tobacco Questions for Surveys: A subset of key questions from the Global Adult Tobacco Survey (GATS). Published online 2011.

[4]Toobert DJ, Hampson SE, Glasgow RE. The Summary of Diabetes Self-Care Activities Measure: results from 7 studies and a revised scale. Diabetes Care. 2000;23(7):943-950.

[5]McGuire BE, Morrison TG, Hermanns N, et al. Short-form measures of diabetes-related emotional distress: the Problem Areas in Diabetes Scale (PAID)-5 and PAID-1. Diabetologia. 2010;53(1):66-69. doi:10.1007/s00125-009-1559-5

[6]Lorig K, Stewart A, Ritter P, Gonazalez V, Laurent D, Lynch J. Outcomes Measures for Health Education and Other Health Care Interventions. Sage Publications; 1996.

[7]Maly RC, Frank JC, Marshall GN, Robin M, Reuben DB. Perceived Efficacy in Patient–Physician Interactions (PEPPI): Validation of an instrument in older persons. J Am Geriatr Soc. 1998;46(7):889-894.

[8]Chew LD, Grill JP, Snyder A, Bradley KA, Nugent SM, Baines AD. Validation of Screening Questions For Limited Health Literacy In A Large VA Outpatient Population. J Gen Intern Med 2008; 23: 561–566.

© 2022 Rosland AM et al. *JAMA Network Open*.

eTable 2. Predictors of Enrollment Among the 1119 Patients With Diabetes Sent a Recruitment Letter

	Eligible, Enrolled (N=239)	Sent Letter, Not Enrolled (N=880)	p value[a]
Age (SD)	60.15 (8.97)	62.89 (7.41)	<0.001
Female	8 (3.35%)	37 (4.20%)	
Male	231 (96.65%)	843 (95.80%)	0.550
Qualification category			
HbA1c >8%	166 (69.46%)	561 (63.75%)	0.009
SBP >150mmHg[b]	51 (21.34%)	267 (30.34%)	
Meet both of the above HbA1c and SBP criteria	22 (9.21%)	52 (5.91%)	

HbA1c, hemoglobin A1c; SBP, systolic blood pressure; SD, standard deviation
[a]p value determined via t-test for continuous measures, or chi-square test for categorical measures
[b]Most recent SBP > 150 mmHg and mean SBP over last 9 months >150mmHg

© 2022 Rosland AM et al. *JAMA Network Open*.

eTable 3. Unadjusted Changes in Patient Outcomes[a] by Study Arm, Baseline to 12 Months

	CO-IMPACT Intervention Mean (SD) N=116 unless noted	Standard Care Mean (SD) N=113 unless noted
**Pre-Specified Primary Outcomes**		
Patient Activation Measure-13 (range 0-100)	+2.94 (9.86) points	+1.78 (10.84) points
UKPDS 5-Year Cardiac Event Risk	+0.14 (6.78) % (N=110)	-0.73 (6.51) % (N=110)
**Pre-Specified Secondary Outcomes**		
HbA1c%	-0.05 (1.38) (N=114)	-0.28 (1.43)
Systolic Blood Pressure, mmHg	-6.41 (18.39) (N=110)	-2.46 (18.58) (N=110)
Total Cholesterol/HDL Ratio	-0.12 (0.99) (N=114)	-0.21 (1.01)
Diabetes Distress (PAID), range 0-20	-0.25 (4.06) (N=114)	-0.62 (4.60) (N=111)
Diabetes Self-Efficacy, range 0-10	+0.26 (1.38) (N=115	+0.23 (1.48)
Efficacy in Healthcare Participation (PEPPI), range 5-25	+0.23 (3.41) (N=115)	+0.33 (4.01)
Self-Management Adherence (SDSCA), range 0-7 days in last week		
Healthy Eating	+1.12 (2.30) days	+0.50 (2.54) days
Physical Activity	+0.09 (2.46) (N=115)	+0.46 (2.13)
Blood Sugar Home Testing	+0.17 (1.75) (N=101)	+0.19 (1.80) (N=94)
Blood Pressure Home Testing	+0.51 (2.52) (N=75)	+0.48 (1.88) (N=62)
Check Feet	+0.72 (2.06) (N=115)	+0.72 (2.26)
Medication Adherence (oral meds)	+0.14 (1.17) (N=99)	+0.23 (1.30) (N=96)
Medication Adherence (Insulin)	+0.14 (1.34) (N=73)	+0.48 (2.08) (N = 61)
Satisfaction with Healthcare System Engagement of Support Person, range 1-5	+0.52 (0.91) (N=107)	+0.33 (1.08) (N=111)

[a]See eTable1 for additional details on measurement approach, measure wording/scales, scoring, and interpretation.
UKPDS, UK Prospective Diabetes Study; HbA1c, hemoglobin A1c; HDL, high-density lipoprotein; PAID, Problem Areas in Diabetes Scale; PEPPI, Perceived Efficacy in Patient-Physician Interactions Questionnaire; SDSCA, Summary of Diabetes Self-Care Activities Measure

© 2022 Rosland AM et al. *JAMA Network Open*.

eTable 4. Intervention Effect on Baseline–to–12-Month Changes in Patient Outcomes From Multivariable Linear Regression, Full Model Results

Patient Outcome	Adjusted Coefficient (95% CI)	Model N
**Patient Activation Measure (PAM)-13**		
Intervention Group (vs Standard Care)	+2.60 (+0.02, +5.18)[a]	229
>Intervention (CO-IMPACT) Arm only	+3.65 (+1.86, +5.45)[a]	
>Standard Care Arm only	+1.05 (-0.77, +2.87)	
Patient-Supporter live together (vs not)	+0.44 (-2.33, +3.21)	
Insulin at baseline (vs no)	-4.41 (-7.02, -1.81)[a]	
PAM baseline >40 (vs <40)	-1.06 (-5.38, +3.25)	
Baseline value of outcome	-0.26 (-0.44, -0.07)[a]	
**UKPDS 5-year Cardiac Event Risk**		
Intervention Group (vs Standard Care)	+1.01 (-0.74, +2.77)	220
>Intervention (CO-IMPACT) Arm only	+0.002 (-0.01, +0.014)	
>Standard Care Arm only	-0.008 (-0.02, +0.004)	
Patient-Supporter live together (vs not)	+0.52 (-1.37, +2.41)	
Insulin at baseline (vs no)	-0.46 (-2.24, +1.33)	
PAM baseline >40 (vs <40)	+0.21 (-1.55, +1.97)	
Baseline value of outcome	-17.65 (-26.98, -8.33)[a]	
**HbA1c%**		
Intervention Group (vs Standard Care)	+0.17 (-0.17, +0.51)	227
Patient-Supporter live together (vs not)	-0.18 (-0.55, +0.20)	
Insulin at baseline (vs no)	+0.18 (-0.17, +0.54)	
PAM baseline >40 (vs <40)	+0.10 (-0.25, +0.44)	
Baseline value of outcome	-0.34 (-0.46, -0.24)[a]	
**Systolic Blood Pressure, mmHg**		
Intervention Group (vs Standard Care)	-2.82 (-7.00, +1.35)	220
Patient-Supporter live together (vs not)	+1.55 (-2.93, +6.04)	
Insulin at baseline (vs no)	+1.20 (-3.01, +5.42)	
PAM baseline >40 (vs <40)	-2.43 (-6.61, +1.74)	
Baseline value of outcome	-0.55 (-0.66, -0.44)[a]	
**Total Cholesterol/HDL Ratio**		
Intervention Group (vs Standard Care)	+0.15 (-0.09, +0.40)	227
Patient-Supporter live together (vs not)	+0.06 (-0.20, +0.32)	
Insulin at baseline (vs no)	-0.17 (-0.41, +0.07)	
PAM baseline >40 (vs <40)	+0.08 (-0.16, +0.33)	
Baseline value of outcome	-0.30 (-0.39, -0.22)[a]	
**Diabetes Distress (PAID)**		
Intervention Group (vs Standard Care)	+0.12 (-0.95, +1.19)	225
Patient-Supporter live together (vs not)	+0.98 (-0.18, +2.14)	
Insulin at baseline (vs no)	+0.95 (-0.15, +2.05)	
PAM baseline >40 (vs <40)	-0.58 (-1.67, +0.50)	
Baseline value of outcome	-0.36 (-0.46, -0.25)[a]	
**Diabetes Self-Efficacy**		
Intervention Group (vs Standard Care)	+0.40 (+0.09, +0.71)[a]	228
Patient-Supporter live together (vs not)	-0.22 (-0.55, +0.12)	
Insulin at baseline (vs no)	-0.26 (-0.57, +0.05)	

© 2022 Rosland AM et al. *JAMA Network Open*.

Patient Outcome	Adjusted Coefficient (95% CI)	Model N
PAM baseline >40 (vs <40)	+0.22 (-0.11, +0.55)	
Baseline value of outcome	-0.56 (-0.66, -0.46)[a]	
**Efficacy in Healthcare Participation (PEPPI)**		
Intervention Group (vs Standard Care)	+0.11 (-0.71, +0.93)	228
Patient-Supporter live together (vs not)	-0.61 (-1.50, +0.28)	
Insulin at baseline (vs no)	-0.56 (-1.38, +0.27)	
PAM baseline >40 (vs <40)	+1.00 (+0.11, +1.90)	
Baseline value of outcome	-0.52 (-0.63, -0.42)[a]	
**Healthy Eating (SDSCA)**		
Intervention Group (vs Standard Care)	+0.71 (+0.20, +1.22)[a]	229
Patient-Supporter live together (vs not)	-0.01 (-0.57, +0.54)	
Insulin at baseline (vs no)	-0.26 (-0.77, +0.26)	
PAM baseline >40 (vs <40)	+0.11 (-0.40, +0.63)	
Baseline value of outcome	-0.63 (-0.73, -0.52)[a]	
**Physical Activity (SDSCA)**		
Intervention Group (vs Standard Care)	-0.04 (-0.56, +0.47)	228
Patient-Supporter live together (vs not)	+0.09 (-0.46, +0.64)	
Insulin at baseline (vs no)	-0.21 (-0.72, +0.31)	
PAM baseline >40 (vs <40)	+0.46 (-0.05, +0.97)	
Baseline value of outcome	-0.59 (-0.71, -0.48)[a]	
**Blood Sugar Home Testing (SDSCA)**		
Intervention Group (vs Standard Care)	+0.23 (-0.22, +0.68)	195
Patient-Supporter live together (vs not)	-0.49 (-0.95, -0.02)[a]	
Insulin at baseline (vs no)	+1.02 (+0.46, +1.58)[a]	
PAM baseline >40 (vs <40)	+0.25 (-0.19, +0.70)	
Baseline value of outcome	-0.50 (-0.62, -0.37)[a]	
**Blood Pressure Home Testing (SDSCA)**		
Intervention Group (vs Standard Care)	+0.47 (-0.25, +1.19)	137
Patient-Supporter live together (vs not)	+0.40 (-0.33, +1.13)	
Insulin at baseline (vs no)	+0.27 (-0.42, +0.97)	
PAM baseline > 40 (vs <40)	-0.24 (-0.94, +0.47)	
Baseline value of outcome	-0.41 (-0.55, -0.27)[a]	
**Check Feet (SDSCA)**		
Intervention Group (vs Standard Care)	+0.26 (-0.22, +0.75)	228
Patient-Supporter live together (vs not)	-0.10 (-0.63, +0.42)	
Insulin at baseline (vs no)	+0.18 (-0.32, +0.68)	
PAM baseline > 40 (vs <40)	-0.02 (-0.51, +0.48)	
Baseline value of outcome	-0.52 (-0.63, -0.41)[a]	
**Medication Adherence (oral medications) (SDSCA)**		
Intervention Group (vs Standard Care)	+0.10 (-0.23, +0.42)	195
Patient-Supporter live together (vs not)	+0.29 (-0.05, +0.64)	
Insulin at baseline (vs no)	-0.22 (-0.54, +0.11)	
PAM baseline > 40 (vs <40)	-0.02 (-0.35, +0.31)	
Baseline value of outcome	-0.38 (-0.50, -0.26)[a]	
**Medication Adherence (Insulin) (SDSCA)**		
Intervention Group (vs Standard Care)	-0.07 (-0.41, +0.26)	134

© 2022 Rosland AM et al. *JAMA Network Open*.

Patient Outcome	Adjusted Coefficient (95% CI)	Model N
Patient-Supporter live together (vs not)	+0.21 (-0.15, +0.56)	
Insulin at baseline (vs no)	[N/A all in model take insulin]	
PAM baseline > 40 (vs <40)	-0.19 (-0.52, +0.15)	
Baseline value of outcome	-0.92 (-1.03, -0.82)[a]	
**Satisfaction with Healthcare System Engagement of Supporter**		
Intervention Group (vs Standard Care)	+0.28 (+0.07, +0.49)[a]	218
Patient-Supporter live together (vs not)	-0.22 (-0.45, +0.01)	
Insulin at baseline (vs no)	+0.07 (-0.14, +0.28)	
PAM baseline > 40 (vs <40)	+0.11 (-0.11, +0.32)	
Baseline value of outcome	-0.68 (-0.78, -0.57)[a]	

[a]Statistical significance $p<0.05$

CI, confidence interval; UKPDS, United Kingdom Prospective Diabetes Study; HbA1c, hemoglobin A1c; PAID, Problem Areas in Diabetes; PEPPI, Perceived Efficacy in Patient-Physician Interactions; PAM, Patient Activation Measure; SDSCA, Summary of Diabetes Self-Care Activities

© 2022 Rosland AM et al. *JAMA Network Open*.

eTable 5. Patient and Supporter Ratings of Experience With CO-IMPACT Intervention Components

At the end of the 12-month intervention period, participants who were assigned to the CO-IMPACT intervention arm were asked to rate their experience with each intervention component. Patients were asked to rate the extent to which the CO-IMPACT components helped them to manage their diabetes. Supporters were asked to rate the extent to which the CO-IMPACT components helped them support their patient partner with diabetes:

Patients: "Did [CO-IMPACT component] help you manage your diabetes?"
Supporters: "Did [CO-IMPACT component] help you support your Patient Partner with their diabetes"

The number of participants with complete data on each item ranges from 104 (for supporters) to 111 (for patients). "N/A" indicates the item was not asked.

**Health coaching session**				
	**Patients (n=111)**		**Supporters (n=104)**	
	**No.**	**%**	**No.**	**%**
Yes, it helped a great deal	44	39.6	42	38.5
Yes, it helped somewhat	52	46.9	51	46.8
No, it really didn't seem to make a difference	15	13.5	10	9.2
No, it seemed to make things worse	0	0	1	0.9
Refused	0	0	0	0
I did not participate in the coaching session	n/a	n/a	1	0.9

**Educational information in CO-IMPACT handbook or website**				
	**Patients (n=110)**		**Supporters (n=105)**	
	**No.**	**%**	**No.**	**%**
Yes, it helped a great deal	29	26.4	25	22.9
Yes, it helped somewhat	62	56.4	48	44.0
No, it really didn't seem to make a difference	19	17.3	22	20.2
No, it seemed to make things worse	0	0	0	0
I didn't receive educational information or access to website	0	0	10	9.2

**IVR automated calls – patients only**		
	**Patients (n=111)**	
	**No.**	**%**
Yes, they helped a great deal	32	28.8
Yes, they helped somewhat	51	45.9
No, they really didn't seem to make a difference	26	23.4
No, they seemed to make things worse	1	0.9
I didn't participate in the automated calls	1	0.9

© 2022 Rosland AM et al. *JAMA Network Open*.

**Emails to supporter summarizing patients' IVR (automated Interactive Voice Response) call responses**				
	**Patients (n=108)**		**Supporters (n=105)**	
	**No.**	**%**	**No.**	**%**
Yes, they helped a great deal	13	12.0	25	22.9
Yes, they helped somewhat	33	30.6	38	34.9
No, they really didn't seem to make a difference	29	26.9	22	20.2
No, they seemed to make things worse	1	0.9	2	1.8
I don't know	32	29.6	0	0
I didn't receive any summaries from the automated calls	n/a	n/a	18	16.5

**Visit prep calls from health coach – patients only**		
	**Patients (n=109)**	
	**No.**	**%**
Yes, they helped a great deal	29	26.1
Yes, they helped somewhat	47	42.3
No, it really didn't seem to make a difference	23	20.7
No, they seemed to make things worse	0	0
I didn't receive any calls to prepare for a visit	12	10.8

**Email reminders to work with patient partner to prepare for visit to primary care provider – supporters only**		
	**Supporters (n=105)**	
	**No.**	**%**
Yes, they helped a great deal	18	16.5
Yes, they helped somewhat	27	24.8
No, it really didn't seem to make a difference	24	22.0
No, they seemed to make things worse	0	0
I didn't receive any email reminders to work with the patient to prepare for their visit to their primary care provider	36	33.0

**Visit summaries**				
	**Patients (n=111)**		**Supporters (n=104)**	
	**No.**	**%**	**No.**	**%**
Yes, they helped a great deal	39	35.1	26	23.9
Yes, they helped somewhat	40	36.0	31	28.4
No, it really didn't seem to make a difference	11	9.9	13	11.9
No, they seemed to make things worse	1	0.9	0	0
I didn't receive any summaries of visits to patient's primary care provider	20	18.0	34	31.2

© 2022 Rosland AM et al. *JAMA Network Open*.

Thinking about all the parts we just reviewed, has the CO-IMPACT program, overall, helped you:

Patients: **manage your diabetes?**
Supporters: **support your Patient Partner with their diabetes**

	Patients (n=111)		Supporters (n=104)	
	No.	%	No.	%
Yes, it helped a great deal	51	46.0	43	39.5
Yes, it helped somewhat	53	47.8	47	43.1
No, it really didn't seem to make a difference	7	6.3	13	11.9
No, it seemed to make things worse	0	0	0	0
Refused	0	0	1	0.9

If the CO-IMPACT program were available at the VA, would you recommend it to another Veteran with diabetes? – patients only

	Patients (n=111)	
	No.	%
Definitely	77	69.3
Probably	31	27.9
Probably not	3	2.7
Definitely not	0	0
Refused	0	0

If this program were available to other people like you supporting family or friends with diabetes, would you recommend this program to them? – supporters only

	Patients (n=105)	
	No.	%
Definitely	70	64.2
Probably	28	25.7
Probably not	6	5.5
Definitely not	0	0
Refused	1	0.9

How well do you and [Patient Partner] work together to manage diabetes now, compared to 12 months ago? – supporters only (n=105)

	Supporters (n=105)	
	No.	%
Much better than a year ago	38	34.9
Somewhat better	32	29.4
A little better	17	15.6
No change	14	12.8
A little worse	2	1.8
Somewhat worse	0	0
Much worse than a year ago	2	1.8

© 2022 Rosland AM et al. *JAMA Network Open*.

eTable 6. Qualitative Themes Among CO-IMPACT Intervention Arm Patient and Supporter Participant Comments

Study RAs used structured interviews at the end of the 12-month intervention period to assess patient and supporter experiences with each of the intervention components, as well as whether and how they work together differently on managing the patient's diabetes.

The interview questions had a two-part structure: a closed-ended inquiry followed by an open-ended probe for more detailed information.

Example:

The first component is the health coaching session you and your Care Partner had with your coach at the beginning of the program. During this session, you received the CO-IMPACT handbook and went over your health numbers, and topics such as getting the most out of your healthcare, action planning, and ways to work together with your Care Partner. Did this coaching session help you manage your diabetes?

- Yes, it helped a great deal
- Yes, it helped somewhat
- No, it really didn't seem to make a difference
- No, it seemed to make things worse
- Refused

IF YES: Please tell more a little about how it helped ___________________

IF NO DIFFERENCE or WORSE: What would have made it (more) helpful? ________________

Responses to the open-ended questions were transcribed verbatim, then reviewed by three study investigators and categorized via consensus using structured themes, as described in the table below.

**Theme**<br>**Definition**	**Illustrative Comments**	**Intervention Component Referring To**	**Quote Source**
**Goal Setting**<br>Comment indicates that the patient and/or supporter began or increased making diabetes-related goals or plans during the CO-IMPACT intervention	*It got me to realize there were things I needed to do I had never thought of such as writing goals.*	Health Coaching Session	Patient
	It helped me because it reminded me to set my goals and keep them.	IVR Calls	Patient
	I have goals now so this helps me stay on track	IVR Calls	Patient
	It helps you sit down and plan a new direction and try to reach the goal.	Visit Summary	Patient
	It gave you ideas of what you could do to make the next two weeks better, coming up with a plan and trying to follow the plan, one thing, verses a handful of things.	IVR Calls	Patient
	It helped me stay on track and on my goals.	IVR Calls	Patient
	Setting the goal was a good idea, because he tends not to want to set goals.	Health Coaching Session	Supporter
	We were able to take stuff and make plans, specifically wrote things down to help.	Health Coaching Session	Supporter
	There were specifics we could talk about and make SMART goals to improve.	IVR Calls	Supporter

© 2022 Rosland AM et al. *JAMA Network Open*.

**Theme**<br><br>**Definition**	**Illustrative Comments**	**Intervention Component Referring To**	**Quote Source**
**Patient Activation**<br><br>Comment indicates that the patient started to engage in new diabetes management behaviors; increased the frequency or quality of existing behaviors; has taken more of an interest in their diabetes management; and/or has gained a new perspective on diabetes during the CO-IMPACT intervention	*It helped us look at it from a different perspective and put more emphasis on getting the answers we needed and doing what we needed to do to solve some of the issues we needed to and realize the only people that could really do it was us.*	Health Coaching Session	Patient
	But there are little things in the workbook that tweaked what I do and it made me look into stuff.	Handbook	Patient
	I started to write things down and think in advance. It is easy to be lazy; it is NOT easy to be healthy.	Visit Prep Calls	Patient
	It made me more aggressive toward what I eat and exercise; it changed my life.	Health Coaching Session	Patient
	It kept me on my toes, I had to make sure I was doing the things that was needed to be done.	IVR Calls	Patient
	A regular reminder that I am in charge of my health	IVR Calls	Patient
	I used the logs as well. I created a statistical program which would signal limits of what I had going on, it generates confidence intervals, doing what I am doing projected ahead it would show where the limit of where blood sugar or blood pressure could go.	Handbook	Patient
	There was a time it [IVR recorded message] was gargled [sic] and his responses were wrong and incorrect things were passed on to his primary care doctor. Which wasn't always a bad thing. He would reach out and talk to his doctor and say it was inaccurate.	Supporter IVR Summaries	Supporter
	It was great to see him keep track of things better!	Health Coaching Session	Supporter
**Supporter Learned <u>how</u> to Help the Patient**<br><br>Comment indicates that the supporter learned new or better ways to help the patient manage their diabetes during the CO-IMPACT intervention	*[Supporter] has learned a great deal, she seems to understand that what happens with me, anything I take can affect my numbers.*	Health Coaching Session	Patient
	I think [supporter] got involved with my numbers, it helps your partner help (not push you) but start take to care of it.	Visit Summaries	Patient
	And [Supporter] would come in, she would ask something I might not have written down.	Visit Prep Calls	Patient
	[Supporter] would get the feedback and then circle back to me and say things like "what’s the problem?" or "how can I help?”	Supporter IVR Summaries	Patient
	I was able to see what causes diabetes and how to help him get his A1c down	Handbook	Supporter

© 2022 Rosland AM et al. *JAMA Network Open*.

Theme<br>Definition	Illustrative Comments	Intervention Component Referring To	Quote Source
	It gave instructions on what to do for him and his issues he reports	Supporter IVR Summaries	Supporter
	I found out more information, things I didn't even know, so I could read up on it and be better prepared to help.	Supporter IVR Summaries	Supporter
	It would prompt me to talk to him to see if there was anything bothering him for him to write down to take to his doctor.	Supporter Reminder Email	Supporter
	She would get the information and then relay it to me to make better plans to help me.	Supporter IVR Summaries	Supporter
	It would prompt me to talk to him to see if there was anything bothering him for him to write down to take to his doctor.	Supporter Reminder Email	Supporter
	It is something to bring me up to speed and let me know where I can help him make progress.	Visit Summaries	Supporter
**Dyad Talking More About the Patient's Diabetes**<br>Comment indicates that patient and supporter are discussing the patient's diabetes management more frequently or more effectively during the CO-IMPACT intervention	*It's just being able to talk to [supporter] back and forth, not something we did before.*	Health Coaching Session	Patient
	Sometimes we talked, she'll ask me how I am feeling and what my numbers are and she always makes it a point of getting healthy stuff to eat. She did this some of the time before this study, but is doing it more.	Health Coaching Session	Patient
	If we didn't have the calls, we would not have talked about it we talk more now than ever.	Supporter IVR Summaries	Patient
	It was really good information and a great way to start conversation with him!	Handbook	Supporter
	It helped me learn how to talk to him about different things.	Supporter IVR Summaries	Supporter
	It made us able to talk about it for once. It helped me know what questions to ask.	Health Coaching Session	Supporter
	It makes us talk about it more.	Handbook	Supporter
	Just that [IVR summary] was a way to communicate, I knew there was a problem, if he didn't say anything, we could talk about it and it made helpful suggestions of what to do.	Supporter IVR Summaries	Supporter
**Increased Patient-Supporter Teamwork**<br>Comment indicates that	*She knew what my appointment was, and saw what my sugar levels and cholesterol levels, were, so we could work together on eating better.*	Visit summaries	Patient

© 2022 Rosland AM et al. *JAMA Network Open*.

Theme<br>Definition	Illustrative Comments	Intervention Component Referring To	Quote Source
patient and supporter increased the amount they work together on managing the patient's diabetes during the CO-IMPACT intervention	*It put [Supporter] and myself on same plane on managing diabetes, making sure we have a well-rounded diet and taking my metformin at a specific time every day.*	Supporter IVR Summaries	Patient
	It brought both of us to understand the importance of what was going on and how to handle things correctly. We talked about it together.	Visit Summaries	Patient
	It also helped me establish teamwork with my [Supporter] and make plans when I have problems such as with my feet.	Health Coaching Session	Patient
	We would both look at them together, she would explain what I didn't understand since she's a nurse and would remind me where I'm at, for example if the A1c was high, so I started exercising as a result of the last A1C since it had gone up.	Visit Summaries	Patient
	Just brings it all to the forefront, we are on the same sheet and it was fantastic, opening the keys to conversation if you are going to work as a team.	Visit Summaries	Patient
	We are trying to be on more of a schedule and better time frame for us, since it's important with sugars.	Handbook	Supporter
	It helped us be more communicative and more aware of how the two of us could work together to improve his diabetes".	Health Coaching Session	Supporter
	[M]ade us think more about working together more than we were.	Health Coaching Session	Supporter
**Dyad Did Not Discuss IVR Summary Emails**<br>Comment indicates that the patient and supporter had little or no discussion of the IVR summary emails<br><br>*Subtheme: Patient and supporter only discussed IVR summaries when they reported a problem.	*I don't know if she received them or not because we did not talk about it.*	Supporter IVR Summaries	Patient
	She would get them, we would talk about them once in a while, but truth be told, we are so busy.	Supporter IVR Summaries	Patient
	She never brought them up to me. I didn't realize she was getting them.	Supporter IVR Summaries	Patient
	She would mention the calls but we have a lot going on so we didn't discuss much.	Supporter IVR Summaries	Patient
	*These summaries included her [Supporter] in my care. She would only mention/bring up the "bad" ones.**	Supporter IVR Summaries	Patient
	*[S]he did mention once that I missed a call but that was the only time.**	Supporter IVR Summaries	Patient
	When there was something derogatory [sic], he would bring it up in conversation, otherwise he	Supporter IVR	Patient

© 2022 Rosland AM et al. *JAMA Network Open*.

Theme<br>Definition	Illustrative Comments	Intervention Component Referring To	Quote Source
	*didn't bring it up.**	Summaries	
	His health problems were always the same, same problems over and over. I did read them. Never discussed them, just read it.	Supporter IVR Summaries	Supporter
	I just skimmed the summaries. No we didn't talk about it.	Supporter IVR Summaries	Supporter
	I received the emails, but didn't read them. Just busy with other things. Never discussed it that I got them.	Supporter IVR Summaries	Supporter
**Suggestions for Improvement**<br>Comment includes suggestions for what would make the component more helpful.<br><br>**Subtheme: Desire for more direct interaction between human coach and patient or between intervention and Supporter	*The biggest helper might have been having meal plans and specific suggestions on meal plans and choices.*	Health Coaching Session	Patient
	It would be nicer if there was more info and if they were explained in more detail.	Patient Visit Summaries	Patient
	If there were more practical examples or scenarios stuff.	Health Coaching Session	Supporter
	*The responses weren't very personalized. Maybe have humans follow up to unanswered questions.***	IVR Calls	Patient
	*It was nothing personal, hard to answer a computer, it would be helpful to have a live person and have different questions.***	IVR Calls	Patient
	*A real person instead of a robot would make it better.***	IVR Calls	Patient
	I think more written information, so I could sit down with [Patient] and go over things.	Health Coaching Session	Supporter
	*I feel like there should be calls to the care partner as well. I may see him doing stuff differently and I'd like to get feedback too.***	IVR Calls	Supporter
	If he had expressed concerns those would have been more helpful but he did not.	Supporter IVR Summaries	Supporter

© 2022 Rosland AM et al. *JAMA Network Open*.

eFigure. CO-IMPACT IVR Topic Flow Chart

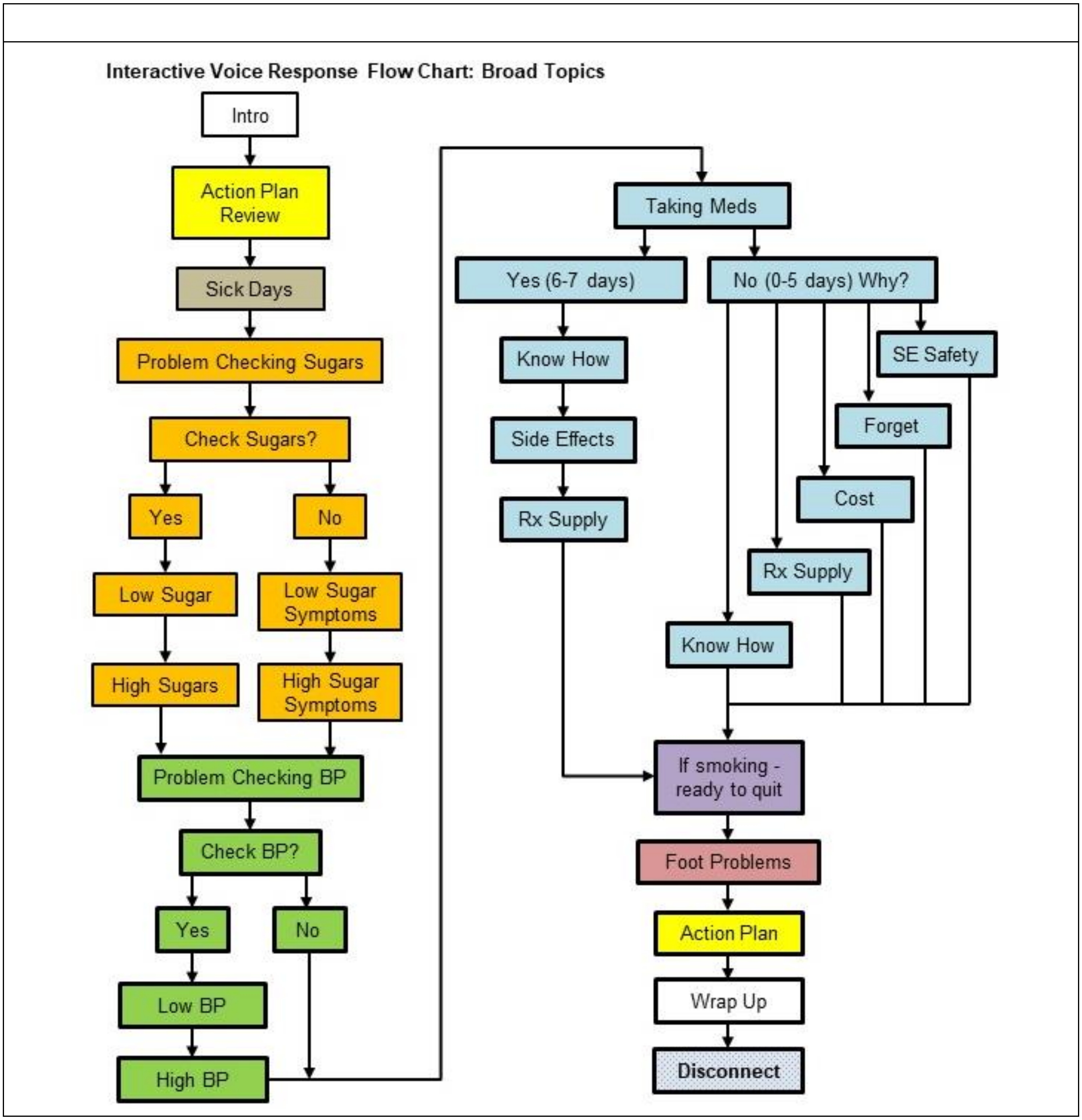

© 2022 Rosland AM et al. *JAMA Network Open*.

eAppendix. CO-IMPACT Intervention Materials Toolkit Location

Effects of Engaging Family Supporters of Adult Patients with Diabetes on Patient Activation, Self-Management, and Clinical Outcomes: A Randomized Clinical Trial

Rosland AM, Piette JD, Trivedi R, Lee AA, Stoll S, Youk A, Obrosky DS, Deverts D, Kerr EA, Heisler ME

With Dissemination Extension support from VA HSR&D, selected CO-IMPACT intervention materials have been converted to Healthcare Professional and Patient/Care Partner Toolkits which can be accessed here: https://www.complexcaring.pitt.edu/co-impact-toolkit

Online CO-IMPACT materials are licensed under Creative Commons Attribution NonCommercial NoDerivs (CC-BY-NC-ND). To view a copy of this license, visit https://creativecommons.org/licenses/by-nc-nd/4.0/.

Additional information on original study materials can be found as online supplements to the trial protocol paper (https://doi.org/10.1186/s13063-018-2785-2).
