# Supplementary material for: Effectiveness of a Health Coaching Intervention for Patient-Family Dyads to Improve Outcomes Among Adults With Diabetes: A Randomized Clinical Trial
Source: JAMA Netw Open. 2022 Nov 14;5(11):e2237960. doi: 10.1001/jamanetworkopen.2022.37960 (PMC9664266; doi:10.1001/jamanetworkopen.2022.37960)
Supplement: Supplement 3. — Data Sharing Statement [file jamanetwopen-e2237960-s003.pdf]

Data Sharing Statement

Rosland. Effectiveness of a Health Coaching Intervention for Patient-Family Dyads to Improve Outcomes Among Adults With Diabetes. *JAMA Netw Open*. Published November 14, 2022. doi:10.1001/jamanetworkopen.2022.37960

Data
Data available: Yes
Data types: Deidentified participant data
How to access data: Final datasets underlying publications resulting from this research will be shared upon request. Members of the scientific community who would like a de-identified copy of the final datasets (i.e., datasets underlying any publication) from this study can request a copy by contacting the PI. De-identified data may only be provided after requestors and data providers sign a standard federal agency Data Use Agreement
When available: With publication

Supporting Documents
Document types: Statistical/analytic code, Informed consent form
How to access documents: Contact study contact (study PI Ann-Marie.rosland@va.gov)
When available: With publication

Additional Information
Who can access the data: Final datasets underlying publications resulting from this research will be shared upon request. Members of the scientific community who would like a de-identified copy of the final datasets (i.e., datasets underlying any publication) from this study can request a copy by contacting the PI. De-identified data may only be provided after requestors and data providers sign a standard federal agency Data Use Agreement
Types of analyses: Analyses included in the published manuscript.
Mechanisms of data availability: With study investigator approval and with a signed data access agreement as above.
